# Synthesis, Isolation, and Characterization of Two Cationic Organobismuth(II) Pincer Complexes Relevant in Radical Redox Chemistry

**DOI:** 10.1021/jacs.2c12564

**Published:** 2023-02-28

**Authors:** Xiuxiu Yang, Edward J. Reijerse, Nils Nöthling, Daniel J. SantaLucia, Markus Leutzsch, Alexander Schnegg, Josep Cornella

**Affiliations:** † Max-Planck-Institut für Kohlenforschung, Kaiser-Wilhelm-Platz 1, 45470, Mülheim an der Ruhr, Germany; ‡ Max Planck Institute for Chemical Energy Conversion, Stiftstrasse 34-36, 45470, Mülheim an der Ruhr, Germany

## Abstract

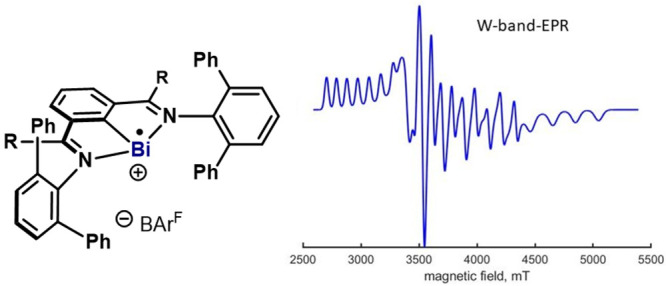

Herein, we report the synthesis, isolation, and characterization of two cationic organobismuth(II) compounds bearing N,C,N pincer frameworks, which model crucial intermediates in bismuth radical processes. X-ray crystallography uncovered a monomeric Bi(II) structure, while SQUID magnetometry in combination with NMR and EPR spectroscopy provides evidence for a paramagnetic *S* = 1/2 state. High-resolution multifrequency EPR at the X-, Q-, and W-band enable the precise assignment of the full *g*- and ^209^Bi *A*-tensors. Experimental data and DFT calculations reveal both complexes are metal-centered radicals with little delocalization onto the ligands.

Characterization of intermediates in chemical processes is of paramount importance to understand and improve their performance. In particular, isolation of paramagnetic complexes involved in single electron transfer (SET) redox events is an arduous task, especially for main group elements, due to their high reactivity.^1^ Bismuth redox catalysis has recently emerged as a versatile platform based on a single p-block element, for which both two-^2^ and one-electron (SET) redox cycles are within reach.^2h,3^ The latter represents a unique reactivity paradigm for a main group element, opening up unusual pathways for organic synthesis. Recently, we have shown that N,C,N bismuth pincer complexes display SET redox reactivity either through one-electron oxidation or homolysis of Bi(III)–X bonds, leading to putative organobismuth(II) intermediates (Figure 1A).^3a,3b^ While the organic radical (X) is readily observed in EPR at room temperature, the Bi(II) species so far escape detection due to their fast relaxation behavior and large magnetic interaction energies. At cryogenic temperature, the dissociation equilibrium shifts to the diamagnetic parent state, thus precluding EPR characterization of the Bi(II) species.

**Figure 1:**
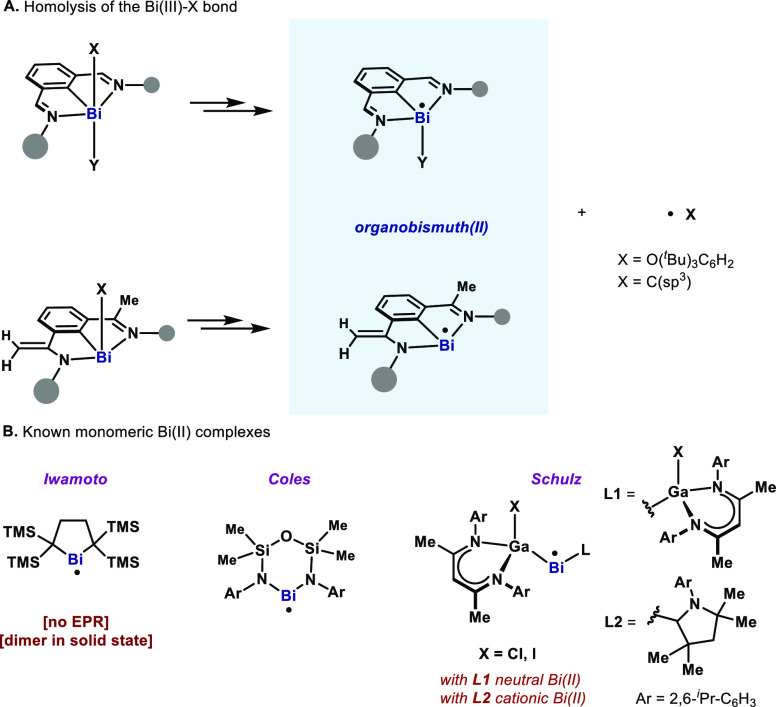
(A) N,C,N organobismuth(II) complexes formed after Bi(III)–X homolysis; (B) state-of-the-art of known Bi(II) complexes.

Bi(II) species have been previously postulated as reactive intermediates,^3a−3c,4^ but their identification and characterization remained challenging. Well-defined, isolated, and characterized Bi(II) complexes are rare, and only a few papers report EPR spectra at low temperature (Figure 1B).^5^ Due to the extremely large ^209^Bi hyperfine interaction (*I* = 9/2, GHz range),^6^ a multifrequency EPR (MF-EPR) approach is required to extract the desired spin Hamiltonian (SH) parameters. In this work, we present the synthesis and isolation of two cationic tridentate N,C,N organobismuth(II) pincer complexes (**3** and **4**). The complexes have been characterized by NMR, MF-EPR, and UV–vis spectroscopy as well as single-crystal X-ray diffraction, HRMS, and SQUID magnetometry. The interpretation of EPR-derived *g*- and hyperfine (*A*) tensors provides decisive details of the electronic and atomic structure. Density functional theory (DFT) calculations facilitated interpretations of the spectroscopic data.

Compounds **3** and **4** were obtained via oxidation of organobismuth(I) precursors **1** and **2** with ferrocenium BAr^F^ (1.0 equiv) (Scheme 1). **3** and **4** were isolated in 92% and 85% yields as purple-brown and orange powders, respectively. The solids are highly sensitive to air but could be stored in the glovebox at −35 °C for several weeks. The ^1^H NMR spectra of **3** and **4** at room temperature show broad peaks from +25.6 to +3.7 ppm and +24.2 to +5.3 ppm, respectively. Most of these peaks shifted to higher field when increasing the temperature (−60 °C to +25 °C), thus pointing to paramagnetic behavior (see SI). The cyclic voltammetry (CV) of **1** and **2** shows a reversible redox wave at −0.42 and −0.64 V (vs Fc/Fc^+^), respectively.

**Scheme 1:**
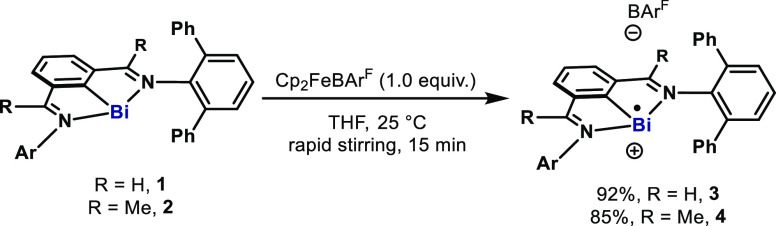
Syntheses of Cationic Bi(II) Complexes

Single-crystal XRD revealed **3** and **4** as monomeric cations (Figure 2). Similarly to precursors **1** and **2**, they contain three-coordinate Bi centers.^2d^ The bismuth atoms reside in the plane formed by N1, C1, and N2 of the ligand. The Bi–Bi distances are 7.26 and 10.74 Å for **3** and **4**, respectively, which are much longer than the sum of the van der Waals radii (∑_vdw_(Bi, Bi) = 4.14 Å).^7^ In **3** and **4**, the Bi–C bonds (2.177(3) and 2.1809(19) Å) are slightly longer than the ones in **1** and **2** (2.1487(19) and 2.1503(18) Å) (Table 1). Interestingly, the Bi–N1 bond in **3** (2.423(3) Å) is shorter than the Bi–N1 distance in **1** (2.4601(15) Å); yet the Bi–N2 distances are similar in both structures. The shorter Bi–N1 bond in **3** compared to **1** is ascribed to the more cationic character of the bismuth. While in the crystal structure of **4** there is one 1,2-difluorobenzene molecule (solvent) close to the bismuth center, both Bi–N bonds are longer than those in **2** (see SI). The distances of N1–C7 and N2–C8 confirm that they are double bonds in **3** and **4**, indicating that both tridentate ligands remain monoanionic. UV–vis spectra of **3** and **4** showed strong bands at 330 nm and broad absorption from 400 to 580 nm (**3**), as well as 330 nm, 360 nm, and broad absorption from 400 to 500 nm (**4**) (see SI).

**Table 1:** Bond Distances (Å) in the Crystal Structure of **1**, **2**, **3**, and **4**

bond	**1**	**3**	**2**	**4**
Bi–C1	2.1487(19)	2.177(3)	2.1503(18)	2.1809(19)
Bi–N1	2.4601(15)	2.423(3)	2.4552(16)	2.4569(18)
Bi–N2	2.5066(15)	2.505(3)	2.4621(15)	2.4786(17)
N1–C7	1.300(2)	1.280(5)	1.305(2)	1.290(3)
N2–C8	1.301(3)	1.284(4)	1.301(2)	1.291(3)

**Figure 2:**
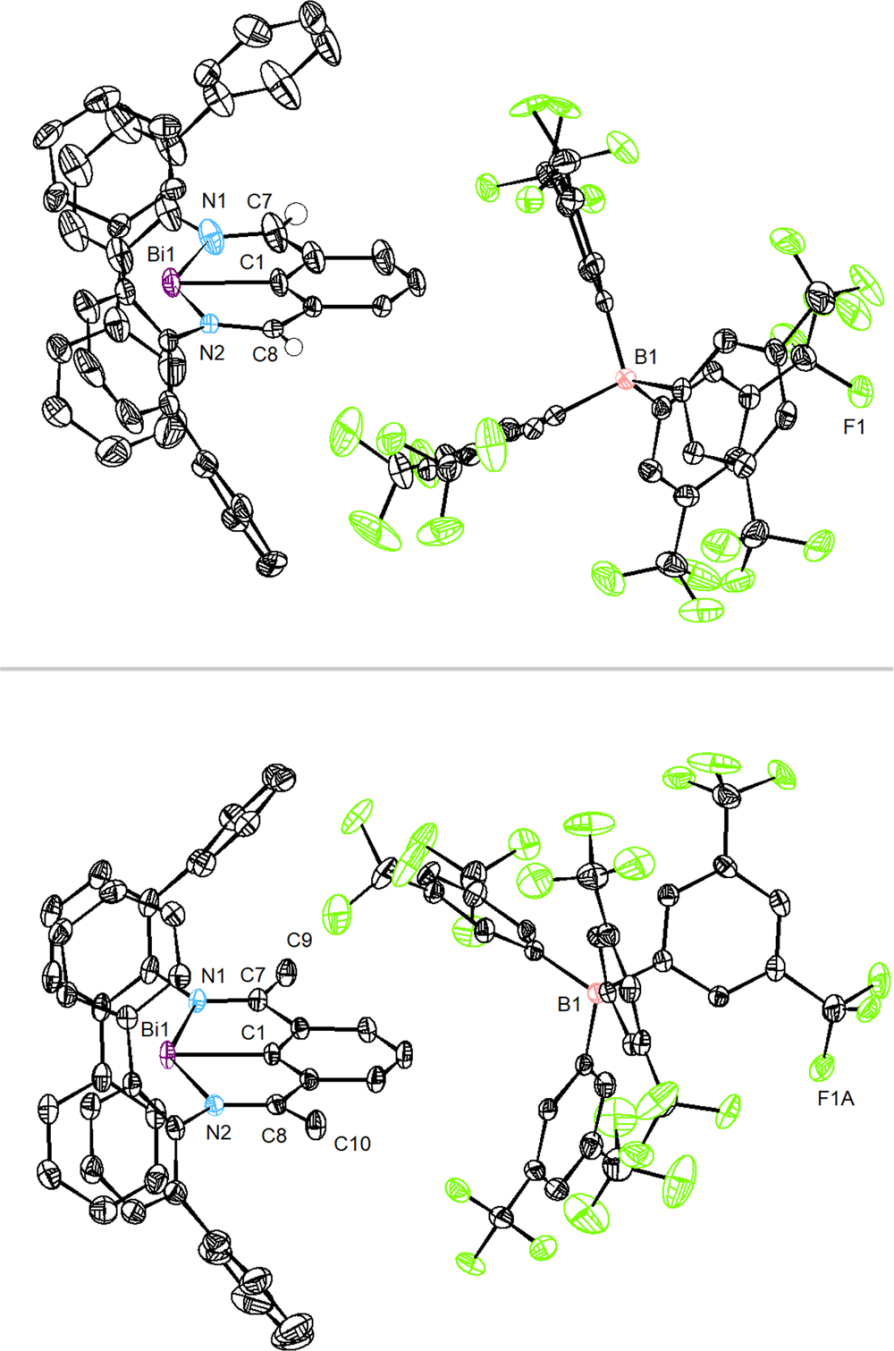
Solid-state structure of **3** (top) and **4** (bottom), using 50% probability ellipsoids. Solvents, hydrogen atoms (except the ones on C7 and C8), and disordered parts have been omitted for clarity.

Figure 3 depicts EPR spectra of **3** and **4** at the X-band (9.6 GHz/0–1.4 T, left), Q-band (34 GHz/0–1.4 T, middle), and W-band (94 GHz/0–6 T, right). For both compounds, the X-band spectra exhibit two main transitions at ∼200 and ∼750 mT. At Q-band several peaks are observed, which can be tentatively assigned to a subset of the at least 10 transitions expected for a *I* = 9/2 nucleus. However, due to the limited field range of the Q-band magnet, only part of the spectrum can be covered (see Figure S15). This limitation is lifted at the W-band, where the multiline spectrum, characteristic for an *S* = 1/2 system with anisotropic *g*- and *A*-tensors, is obtained. For a fully resolved spectrum 10 lines at each of the three *g*-values would be expected. However, since some of the lines at *g*_*x*_ and *g*_*y*_ as well as *g*_*y*_ and *g*_*z*_ overlap, less than 30 lines can be identified. To extract the *g*- and *A*-values, a simultaneous fit of the X-, Q-, and W-band spectrum with the following SH was performed:
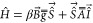
1

**Figure 3:**
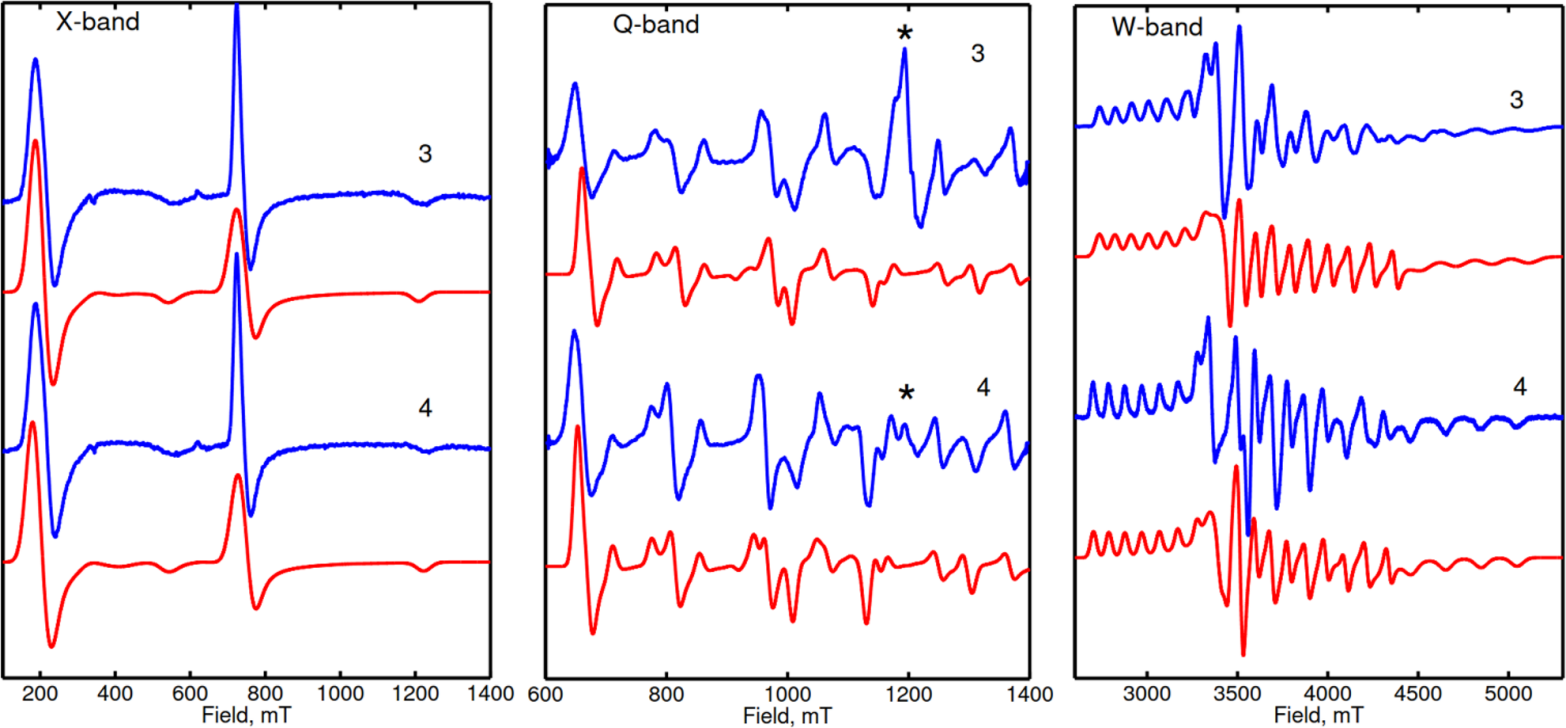
EPR at the X-, Q-, and W-band of **3** and **4** (blue) with simulations (red) according to the SH parameters from Table 2. The marked (*) lines at the Q-band originate from a *g* ∼ 2 impurity.

In the simulations, the *g*- (*g̿*) and *A*-tensors (*A̿*) are the magnetic interaction tensors characteristic of the Bi(II) *S* = 1/2 center (eq 1). These tensors were assumed to be collinear. Simulations obtained with *g*- and *A*-values derived from fits to the experimental spectra (Table 2) are plotted as red lines in Figure 3. In addition, plots of the spin energy levels vs the external magnetic field (Breit–Rabi diagram) have been calculated for the X-band (Figure 4A–C), Q-band, and W-band (Figure S14) to rationalize the EPR transitions.

**Figure 4:**
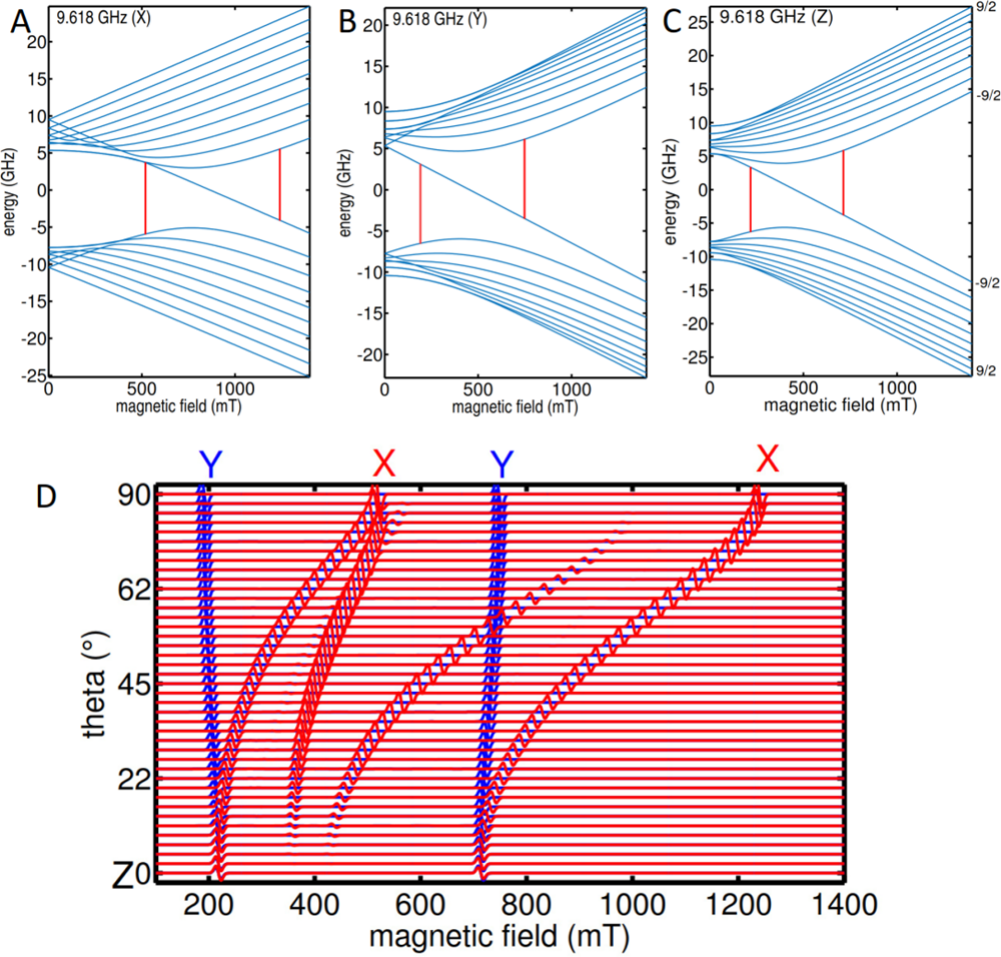
(A to C) Magnetic field dependent spin energy levels (Breit–Rabi diagrams) along the *x*-, *y*-, and *z*-axis of the *g*-tensor. X-band EPR transitions are plotted as vertical red lines. (D) Corresponding roadmap representing the angular dependence of EPR resonances (theta: angle between the magnetic field and the *z*-axis of the *g*-tensor). Energy levels and EPR spectra have been obtained with SH parameters for **4**.

**Table 2:** Principal Values of the Bi(II) *g*- and *A*-Tensors (in MHz) as Well as the Bi(II) Spin Populations (*P*) According to Spectral Simulation and DFT Calculations

	*g*_*x*_	*g*_*y*_	*g*_*z*_	*A*_*x*_	*A*_*y*_	*A*_*z*_	*P*(8)
**3**	1.569	1.709	2.094	4106^a^	2432^a^	3003^a^	0.86
**3-DFT**	1.538	1.994	2.159	–8618	–2902	–6788	0.87
**4**	1.589	1.719	2.119	4106^a^	2377^a^	3003^a^	0.88
**4-DFT**	1.579	1.994	2.195	–8701	–2972	–6916	0.89

a EPR experiments are insensitive to the sign of *A*_*x,y,z*_.

At zero field, the spin energy levels are determined by the *A*-tensor, which, in the coupled representation, gives rise to a total spin *F* = 10/2 manifold (upper levels) as well as a total spin *F* = 8/2 manifold (lower levels). In the range from 0 to 1400 mT, the energy level configuration changes from the low-field picture via the intermediate-field to the high-field picture, where the *S* = 1/2 and *I* = 9/2 spins are decoupled, giving rise to two *I* = 9/2 multiplets. The X-band transitions occur in the intermediate field situation where the spin eigenstates are strongly mixed. The lowest energy level of the *F* = 10/2 multiplet crosses over to join the lower *I* = 9/2 multiplet going from the low- to high-field pictures. The two X-band transitions share this crossing energy level (Figure 4D). These transitions are relatively isotropic in the YZ-plane, giving rise to the dominant spectral features at 200 and 700 mT. In the XZ plane, there is strong anisotropy causing the X-features to be rather weak. At the Q- and W-band, the transitions occur in the high-field regime and can be readily interpreted (Figure S13).

DFT was employed to calculate *g*- and *A*-values (Table 2, Table S1). Calculated *g-*values and collinear orientations of *g-* and *A-*tensors reproduce the experimental findings well, using state-of-the-art DFT methods with different functionals. In addition, the spin population at the Bi atom (*P*) obtained directly from the calculated molecular orbitals matches the values from experiment.^5a,9^ However, as expected for Bi(II)^5a^ and other heavy metal complexes,^10^ the ^209^Bi hyperfine couplings are overestimated.

DFT indicated that the spin density of the unpaired electron is mainly located on the Bi atom, with minimal delocalization onto the ligands (see SI). Yet, some spin density can be observed at the nitrogens N1 and N2, giving rise to nitrogen electron spin–echo envelope modulations (ESEEM) (Figure S16). However, these transitions are ill resolved, resulting in an unclear assignment of the nitrogen *A*-values.

Effective magnetic moments (μ_eff_) were determined to be 1.80 ± 0.06 μ_B_ by Evans’ method at 298 K for both **3** and **4**.^11^ These values are in good agreement with the theoretical value (1.73 μ_B_) for a single unpaired electron.^12^ This assignment was further corroborated by SQUID magnetometry. The magnetic susceptibilities (Figure 5) of both compounds **3** and **4** are similar, with a steady linear increase in χ_P_*T* from low to high *T* over most of the temperature range. For compound **3**, χ_P_*T* at 300 K is 0.482 cm^3^ K mol^–1^ (μ_eff_ = 1.96 μ_B_), consistent with the value obtained at 298 K via Evans’ method NMR (1.80 μ_B_). The data for **4** exhibit an outlier at 300 K; the value for χ_P_*T* at 271 K is 0.367 cm^3^ K mol^–1^ (μ_eff_ = 1.71 μ_B_), and extrapolation of the model to the data suggests that χ_P_*T* approaches ∼0.375 cm^3^ K mol^–1^ (μ_eff_ = 1.73 μ_B_) at room temperature, also consistent with the value obtained at 298 K via Evans’ method NMR (1.80 μ_B_). These values are consistent with a single unpaired electron *S* = 1/2 system, for which the expected value for χ_P_*T* is 0.376 cm^3^ K mol^–1^ (μ_eff_ = 1.73 μ_B_) for *g* = 2. Both the magnetometry and variable-temperature NMR data show that there is substantial temperature-independent paramagnetism (TIP)^13^ present in both compounds, which is shown by the steady linear increase in χ_P_*T* from low to high temperatures. Thus, it is the low-temperature limits of χ_P_*T* that should be considered.

**Figure 5:**
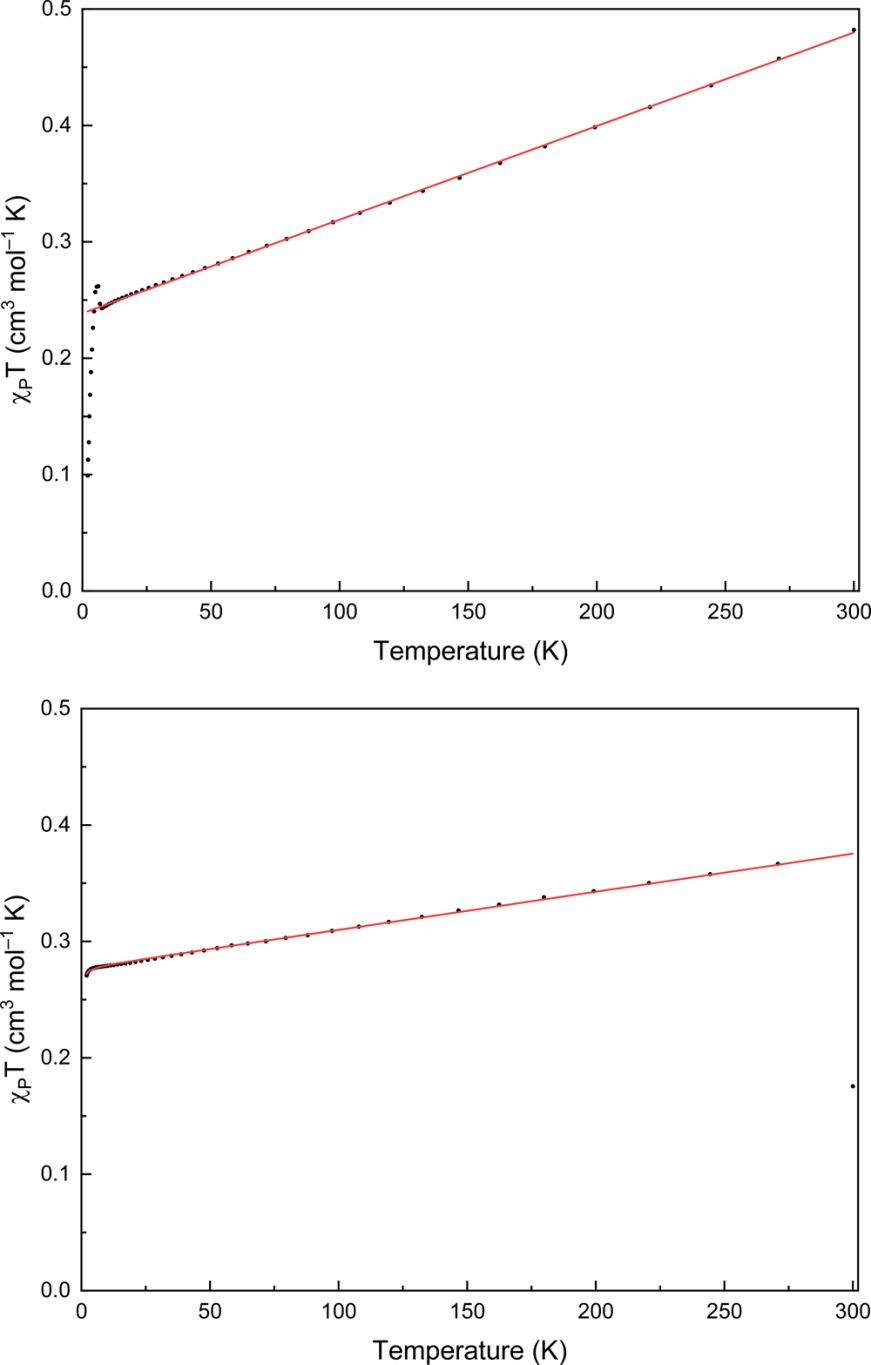
DC magnetic susceptibility data from 2.0 to 300 K (black) and fits from the SH model from 7.6 to 300 K and 2 to 271 K (red traces) for **3** (top) and **4** (bottom), respectively.

The values for χ_P_*T* decrease steadily with temperature until reaching 7.6 K in **3** and 4.1 K in **4**. The value for χ_P_*T* at 7.6 K in **3** is 0.243 cm^3^ K mol^–1^ (μ_eff_ = 1.39 μ_B_) and the value for χ_P_*T* at 4.1 K in **4** is 0.278 cm^3^ K mol^–1^ (μ_eff_ = 1.49 μ_B_), significantly less than the expected value for χ_P_*T* of 0.376 cm^3^ K mol^–1^ (μ_eff_ = 1.73 μ_B_) for *g* = 2. This was also observed previously for a Bi(II) radical.^5a^ The obtained *g*-values from the fits to the data are *g* = 1.60(5) and 1.72(5) for **3** and **4**, respectively. These *g*-values are consistent with the average values obtained from the EPR simulations of *g*_avg_ = 1.804 (**3**) and *g*_avg_ = 1.823 (**4**).^14^ All fit parameters for **3** and **4** are reported in the SI. The average *g*-values are less than the free electron value, *g*_e_ = 2.002319, consistent with substantial spin–orbit coupling at the Bi center with a less than half-filled p-shell.

In this article, we provide the synthesis, isolation, and complete characterization of two cationic organobismuth(II) compounds bearing N,C,N pincer frameworks (**3** and **4**), thus filling an important gap in the understanding of open-shell Bi compounds. Importantly, they mimic elusive Bi(II) states in SET redox processes, whose structural and electronic properties could be studied in detail here for the first time. Solid-state structures for both compounds provided conclusive data about their connectivity revealing monomeric Bi(II). NMR, EPR spectroscopy, and SQUID magnetometry gave clear evidence for a paramagnetic *S* = 1/2 compound. MF-EPR yielded Bi(II) spectra with unmatched resolution. The high spectral quality allowed for the very accurate assignment of the *g*- and ^209^Bi *A*-tensors. This confirmed that the electronic structures of complexes **3** and **4** are very similar and provide a solid model for other neutral Bi(II) radical intermediates. Beyond the characterization of compounds **3** and **4**, the obtained accurate magneto-structural correlations provide unique benchmarks for the further refinement of quantum chemical calculations on compounds containing Bi and other very heavy elements. Since radical chemistry based on low-valent Bi pincer complexes just recently emerged, isolation and characterization of **3** and **4** represent unique examples toward the elucidation of open-shell intermediates. These complexes are of capital importance to understand and design methodologies based on Bi-radical catalysis.

## Supporting Information Available

Experimental procedures and analytical data (^1^H, ^11^B, and ^19^F NMR, HRMS, IR, CV, elemental analysis, MF-EPR, SQUID, UV–vis, and theoretical calculations) for new compounds (PDF)
