# Supplementary material for: Synthesis, Isolation, and Characterization of Two Cationic Organobismuth(II) Pincer Complexes Relevant in Radical Redox Chemistry
Source: J Am Chem Soc. 2023 Feb 28;145(10):5618–23. doi: 10.1021/jacs.2c12564 (PMC10021010; doi:10.1021/jacs.2c12564)
Supplement: Supplementary file 1 — ja2c12564_si_001.pdf [file ja2c12564_si_001.pdf]

# Synthesis, Isolation and Characterization of Two Cationic Organobismuth(II) Pincer Complexes Relevant in Radical Redox Chemistry

Xiuxiu Yang,[a] Edward J. Reijerse,[b] Nils Nöthling,[a] Daniel J. SantaLucia,[a] Markus Leutzsch,[a] Alexander Schnegg,[b]* Josep Cornella[a]*

[a]*Max-Planck-Institut für Kohlenforschung, Kaiser-Wilhelm-Platz 1, 45470, Mülheim an der Ruhr, Germany*

[b]*Max-Planck-Institut für Chemische Energiekonversion, Stiftstrasse 34-36, 45470, Mülheim an der Ruhr, Germany*

1 General methods .......... 3

2 Preparation of bismuth complexes .......... 5

2.1 Preparation of **3** .......... 5

2.2 Preparation of **4** .......... 6

3 NMR spectra of bismuth compounds .......... 7

4 Effective magnetic moments ($\mu_{eff}$) measurement with Evans method .......... 15

5 UV-vis .......... 20

6 IR .......... 22

7 Cyclic Voltammetry .......... 22

8 EPR .......... 24

9 Computational Details .......... 29

10 SQUID .......... 33

11 XRD .......... 34

11.1 Structure search in the Cambridge Structural Database (CSD) .......... 34

11.2 Structural discussions and comparison .......... 35

11.3 Intermolecular interactions .......... 38

11.4 Single crystal structure analysis of compound **3**·THF·pentane solvate .......... 40

11.5 Single crystal structure analysis of compound **4**·1,2-difluorobenzene solvate .......... 55

# 1 General methods

All experiments were performed under argon atmosphere using standard Schlenk and glovebox techniques. THF, *n*-pentane, $Et_2O$, 1,4-dioxane, and THF-$d_8$ were degassed and distilled from sodium/benzophenone and stored with 4Å molecular sieves under argon. 1,2-difluorobenzene was degassed and stored with 4Å molecular sieves under argon. Complex **1**, **2** and $Cp_2FeBAr^F$ were prepared by published methods.[1]

NMR spectra were recorded on a Bruker Avance III HD NanoBay 300 MHz ($^{19}$F NMR), 400 MHz Bruker Avance III HD ($^{11}$B NMR), or Bruker Avance III 500 MHz NMR spectrometer ($^{1}$H NMR). Chemical shifts are reported in ppm relative to TMS and residual chemical shifts of the solvent as the secondary standard (for $^{1}$H). $^{19}$F chemical shifts are reported relative to $CFCl_3$ ($\delta$ = 0 ppm). $^{11}$B chemical shifts are reported relative to $BF_3{\cdot}OEt_2$ ($\delta$ = 0 ppm).

Single crystals suitable for X-ray diffraction were protected with polyisobutylene oil at -30 °C, and then transferred to the goniometer of a Bruker-AXS Kappa Mach3 with an APEX-II detector, IµS microfocus Mo-anode X-ray source and Incoatec Helios mirrors. The structures were solved using SHELXT (G. M. Sheldrick, 2015) and refined using SHELXL (G. M. Sheldrick, 2015) implemented in Olex2 (version 1.5; Dolomanov et al., 2019).

The HRMS analyses were performed using a Thermo Scientific 12 T LTQ-FT mass spectrometer equipped with an APCI source. UV-vis spectra were recorded at a Thermo Fisher Evolution 300 spectrophotometer. IR spectra were recorded using Bruker ALPHA FT-IR spectrometer under ambient conditions with 16 scans on a diamond ATR unit. Elemental analysis were measured by MikroLab Kolbe in Oberhausen in Germany.

CW EPR spectra at X-band were recorded on a Bruker Elexsys 500 CW EPR spectrometer equipped with an ER4116DM resonator and an ESR900 Oxford Helium Flow Cryostat. The modulation amplitude was set to 7.14 Gauss (maximum available for the employed resonator). The microwave power was set to 1 mW. Q-band pulse EPR measurements were performed using a Bruker Elexsys E580 EPR X-band Spectrometer with a homebuilt Q-band extension capable of delivering 34 GHz microwave pulses up to 10W. The experiments were conducted in a homebuilt Q-band Pulse-ENDOR resonator.[2] Electron spin echo (ESE)-detected field-swept spectra were measured at 7-10 K using the pulse sequence: $t_p$–$\tau$–$2t_p$–$\tau$–echo with typical values

$t_p$=20 ns, $\tau$=300 ns, and a repetition time of 1 ms. Subsequently, a pseudomodulation transformation was applied to obtain EPR spectra comparable with standard 1$^{st}$ derivative CW EPR. W-band pulse EPR measurements were performed using a Bruker Elexsys E680 spectrometer equipped with a homebuilt W-band extension and a cryogen free Cryogenic 6 T magnet with a variable temperature insert. ESE-detected field-swept spectra were measured at 10 K using the pulse sequence: $t_p$–$\tau$–2$t_p$–$\tau$–echo with typical values $t_p$=20 ns, $\tau$=260 ns, and a repetition time of 1 ms. Subsequently, a pseudomodulation transformation was applied to obtain EPR spectra comparable with standard 1$^{st}$ derivative CW EPR.[3] All the simulations were obtained using Easyspin 5.2.30 with the function "pepper".[4]

# 2 Preparation of bismuth complexes

## 2.1 Preparation of **3**

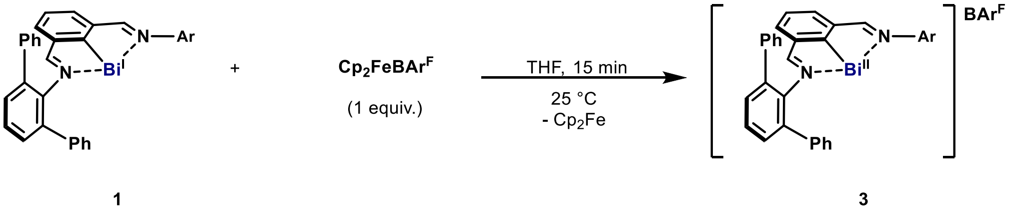

To a mixture of **1** (100 mg, 0.126 mmol) and $Cp_2FeBAr^F$ (132 mg, 0.126 mmol), 10 mL of THF was added quickly under fast stirring. The initial dark purple color turned quickly to purple-brown. After 15 min, the solvent was removed under high vacuum, and the solid was washed 10 times with a 1:5 $Et_2O$:pentane mixture (10 × 5 mL) to remove $Cp_2Fe$. The remaining solid was extracted with $Et_2O$, and the filtrate was dried under high vacuum. Yield: 195 mg, 92%.

**$^1$H NMR (500 MHz, THF-*$d_8$*)** δ 25.63 (bs), 11.67 (bs), 9.61 (bs), 8.69 (bs), 8.19 (bs), 7.78 (s, $BAr^F$), 7.55 (s, $BAr^F$), 3.69 (bs).

**$^{11}$B NMR (128 MHz, THF-*$d_8$*)** δ -6.51.

**$^{19}$F NMR (282 MHz, THF-*$d_8$*)** δ -63.39.

**APCI HRMS** calc'd for $C_{44}H_{31}BiN_2^+$ 796.22857; found: 796.22806.

**APCI HRMS** calc'd for $C_{32}H_{12}BF_{24}^-$ 863.06543; found: 863.06617.

**Elemental analysis** calc'd for $C_{76}H_{43}BBiF_{24}N_2$, C 54.99, H 2.61, N 1.69, B 0.65, F 27.47, Bi 12.59 %; found: C 54.94, H 2.57, N 1.68, B 0.62, F 27.44, Bi 12.62 %.

Note: The errors of H and B are beyond the 0.4 % (1.5 % and 4.6 %, respectively), and this might be attributed to the error combined with the small percentage.

## 2.2 Preparation of **4**

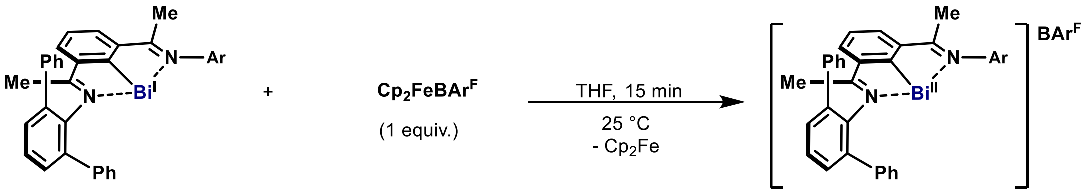


2

4

To a mixture of **2** (100 mg, 0.121 mmol) and $Cp_2FeBAr^F$ (127.2 mg, 0.121 mmol), 10 mL of THF was added quickly under fast stirring. The initial dark brown color turned quickly to orange. After 15 min, the solvent was removed under high vacuum, and the solid was washed 10 times with a 1:5 $Et_2O$:pentane mixture (10 × 5 mL) to remove $Cp_2Fe$. The remaining solid was extracted with $Et_2O$, and the filtrate was dried under high vacuum. Yield: 165 mg, 85%.

**$^1$H NMR (500 MHz, THF-*d₈*)** δ 24.18 (bs), 9.77 (bs), 9.14 (bs), 8.72 (bs), 7.78 (s, $BAr^F$), 7.55 (s, $BAr^F$), 5.31(bs).

**$^{11}$B NMR (128 MHz, THF-$d_8$)** δ -6.51.

**$^{19}$F NMR (282 MHz, THF-$d_8$)** δ -63.39.

**APCI HRMS** calc'd for $C_{46}H_{35}BiN_2^+$ 824.25987; found: 824.25942.

**APCI HRMS** calc'd for $C_{32}H_{12}BF_{24}^-$ 863.06543; found: 863.06627.

**Elemental analysis** calc'd for $C_{78}H_{47}BBiF_{24}N_2$, C 55.50, H 2.81, N 1.66, B 0.64, F 27.01, Bi 12.38 %; found: C 55.36, H 2.79, N 1.64, B 0.65, F 26.94, Bi 12.41 %.

# 3 NMR spectra of bismuth compounds

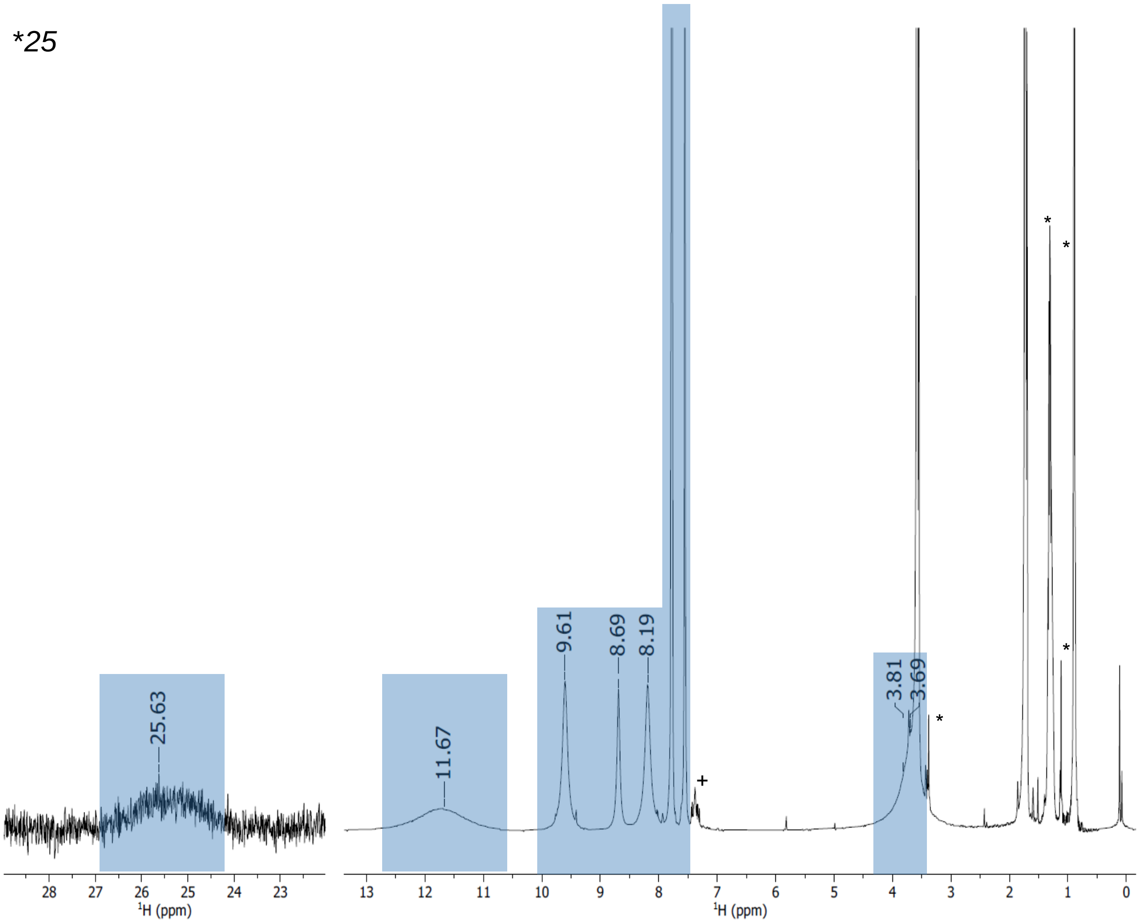


Figure S1. $^1$H NMR spectrum of complex **3** in THF-$d_8$. *Residual $Et_2O$ and hexane. + Unidentified impurities.

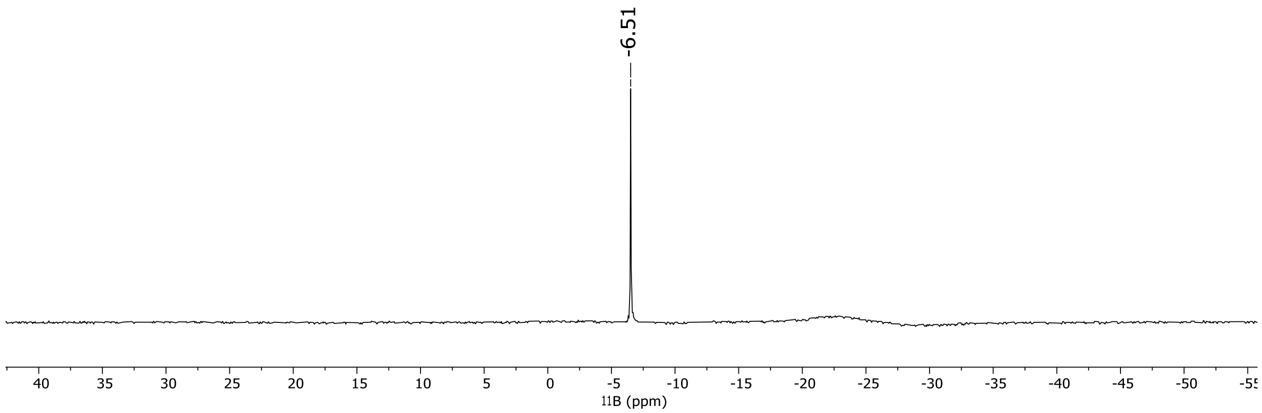


Figure S2. $^{11}B$ NMR spectrum of complex **3** in THF-$d_8$.

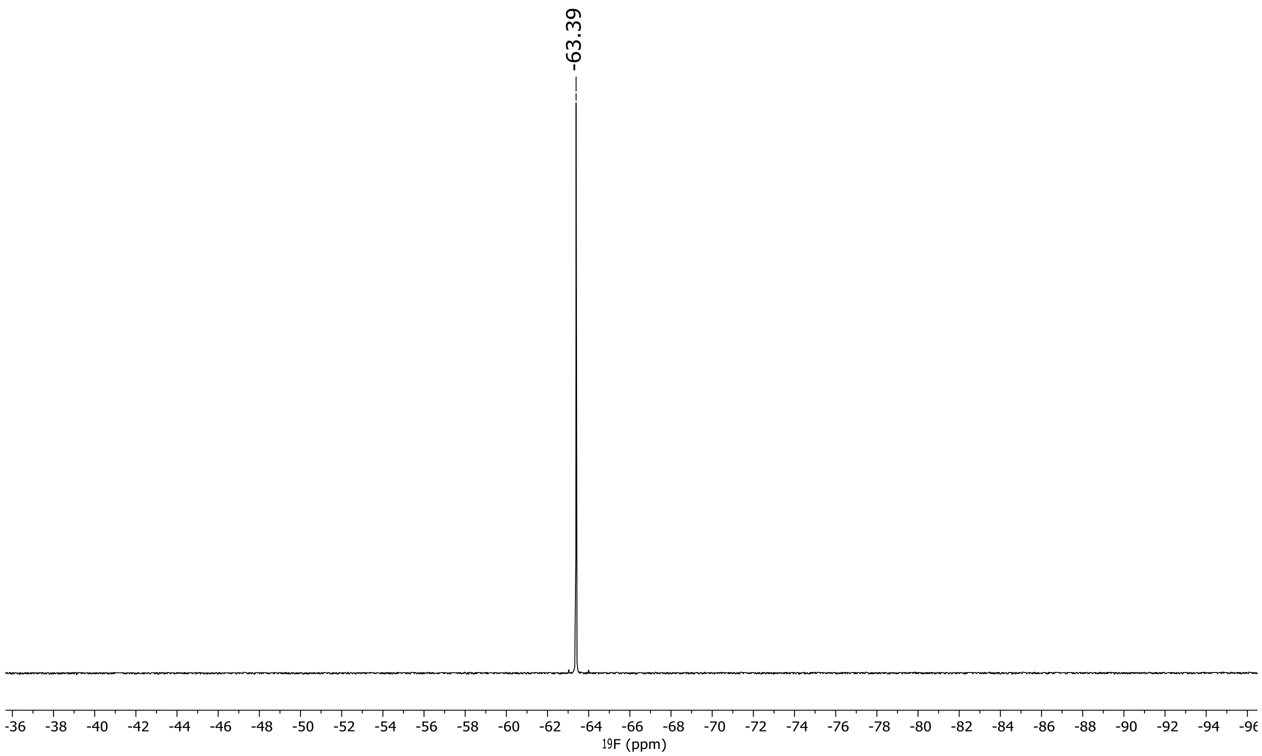


Figure S3. $^{19}F$ NMR spectrum of complex **3** in THF-$d_8$.

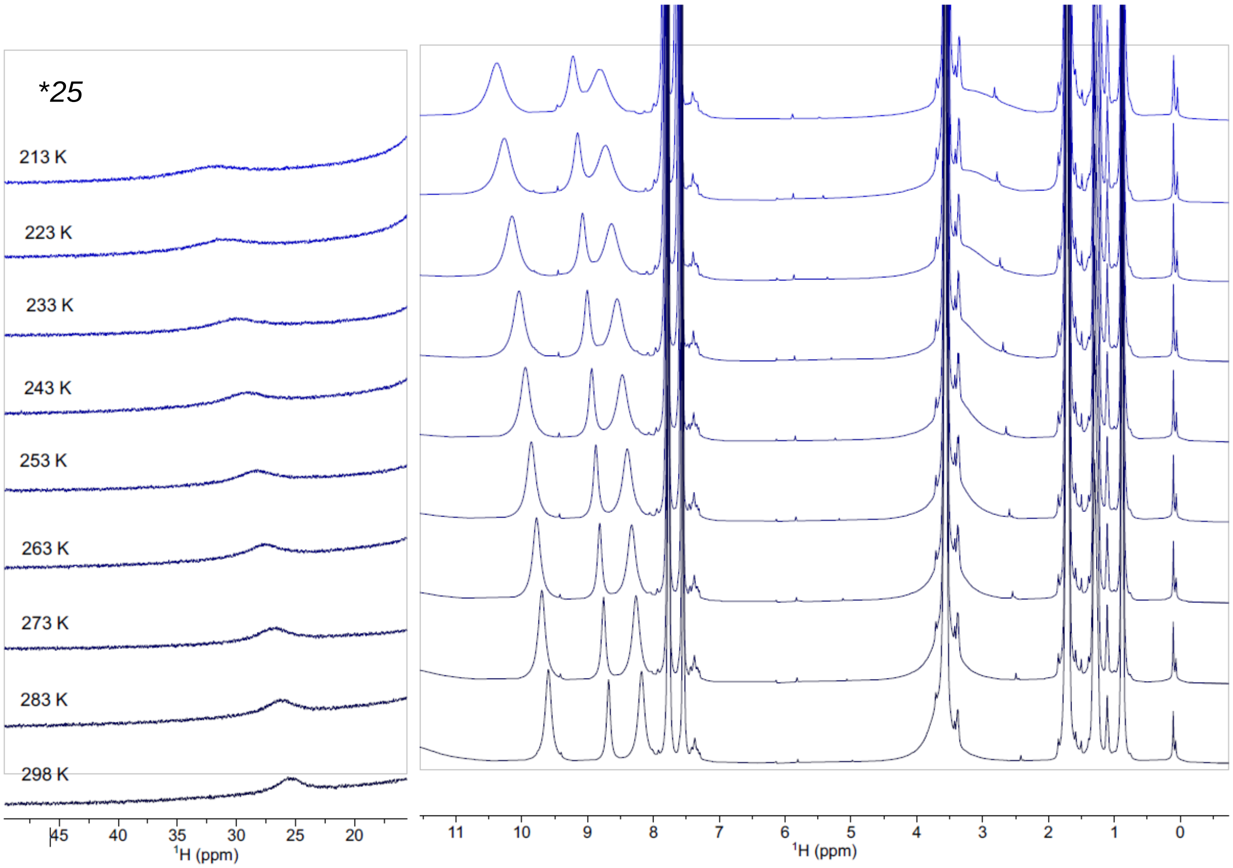


Figure S4. VT $^1$H NMR spectrum of complex **3** in THF-$d_8$ from 213 K to 298 K.

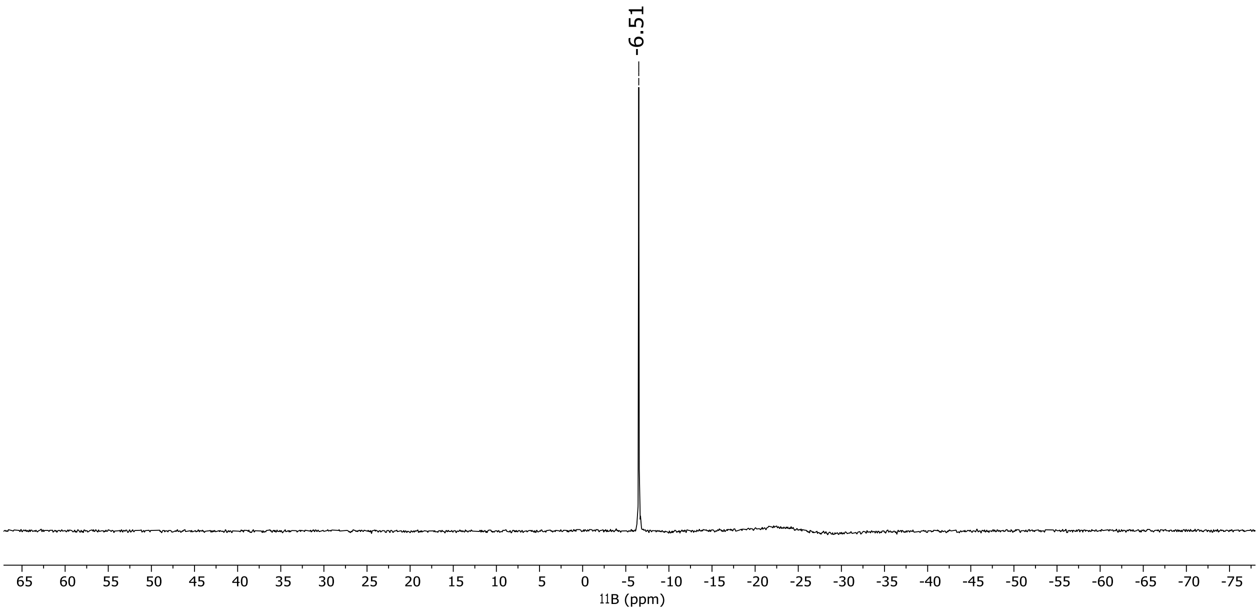


Figure S5. $^{11}$B NMR spectrum of complex **4** in THF-$d_8$.

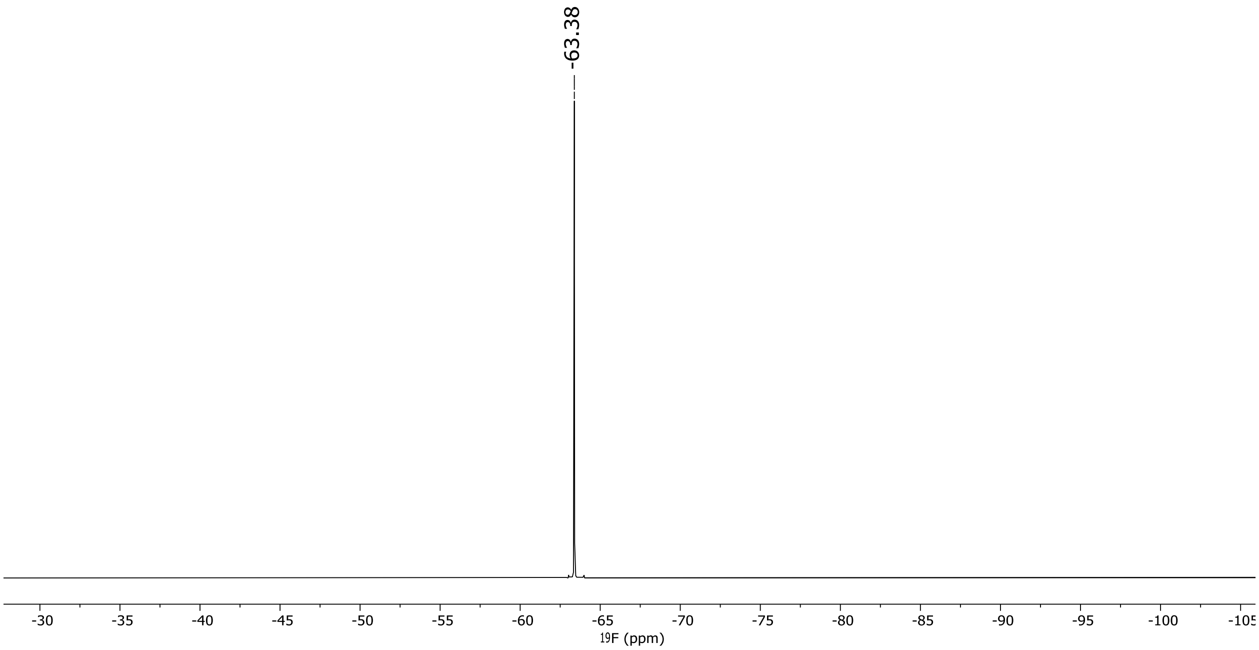


Figure S6. $^{19}F$ NMR spectrum of complex **4** in THF-$d_8$.

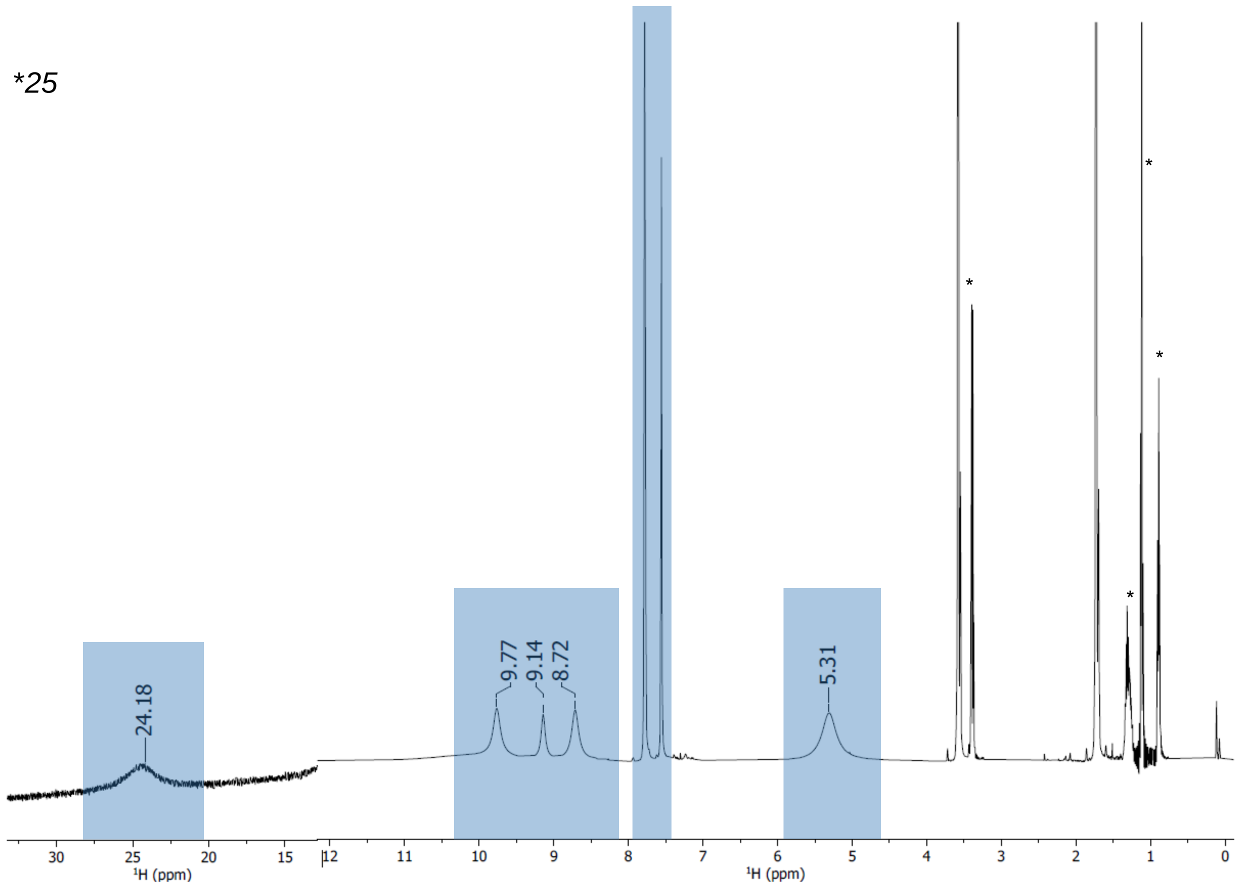


Figure S7. $^1$H NMR spectrum of complex **4** in THF-$d_8$. *Residual $Et_2O$ and hexane.

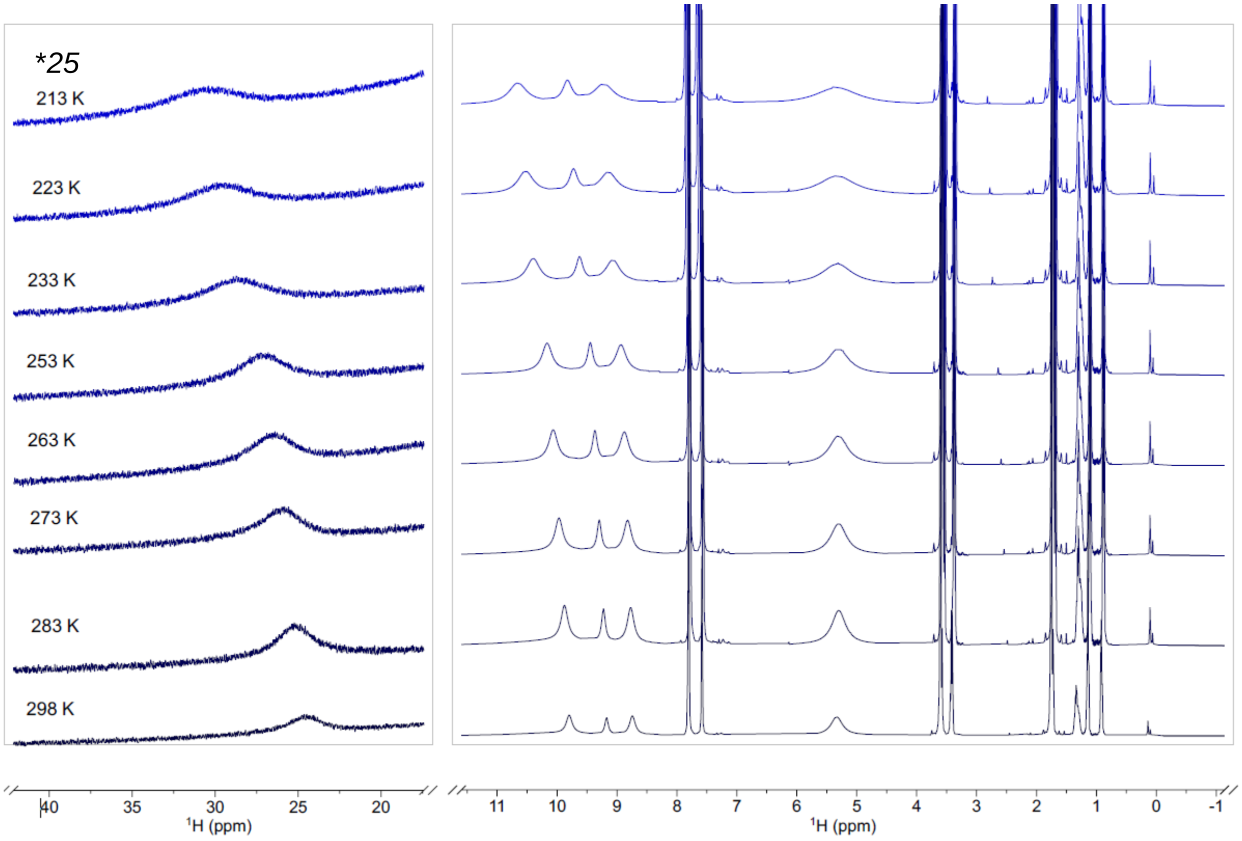


Figure S8. VT $^{1}H$ NMR spectrum of complex **4** in THF-$d_8$ from 213 K to 298 K.

# 4 Effective magnetic moments ($\mu_{eff}$) measurement with Evans method

Inside the glovebox, **3** or **4** (15 mg) was dissolved in 0.6 mL THF-$d_8$ in a J-Young NMR tube which contained a sealed capillary with pure THF-$d_8$. $^1$H NMR spectra were recorded at room temperature, then in increments of 10 °C from -60 °C to 25 °C. Then, a 50 µL stock solution of 1,3,5-trimethoxybenzene (1 mg) was injected into the NMR tube. A $^1$H NMR spectrum was subsequently recorded at 25 °C with a long relaxation delay ($d_1$ = 70 s) to determine the accurate amount of **3** or **4** by integrating the ArCH signal of the BArF counter anion against the standard. By analyzing the differences between the THF-$d_8$ peaks from the sample containing **3** or **4** with the peaks from the capillary, it is possible to calculate effective magnetic moments ($\mu_{eff}$) at each temperature following literature procedures:[5]

$$\mu_{eff} = \sqrt{8 * \chi_p T} \qquad (S1)$$

$$\chi_P = \chi_m - \chi_D \qquad (S2)$$

$$\chi_m = \left| \frac{-3000\Delta f}{4\pi f c} \right| \qquad (S3)$$

Whereas $\chi_m$ = molar susceptibility (cm³/mol), $\Delta f$ = frequency difference to reference without paramagnetic species (Hz), $f$ = NMR frequency (Hz), $c$ = molar concentration (M), $\chi_P$ = paramagnetic susceptibility (cm³/mol), $\chi_D$ = diamagnetic susceptibility (cm³/mol).

*Error estimations of the above values are based on propagation of uncertainty:*

ΔΔf: The values is the standard deviation of the Δf obtained by comparing the two residual THF-$d_8$ solvent ($\Delta f_1$: -OC**H**DCD$_2$-, $\Delta f_2$: -OCD$_2$C**H**D-) peaks against the reference

$$\Delta\chi_m = \left| \chi_m \sqrt{\left(\frac{\Delta\Delta f}{\Delta f}\right)^2 + \left(\frac{\Delta c}{c}\right)^2} \right| \qquad (S4)$$

$$\Delta\chi_P = \sqrt{\Delta\chi_m{}^2 + \Delta\chi_D{}^2} \qquad (S5)$$

$$\mu_{eff} = 0.5 \times \mu_{eff}\sqrt{(\frac{\Delta\chi_P}{\chi_P})^2 + (\frac{\Delta T}{T})^2} \qquad (S6)$$

The diamagnetic susceptibility of the complexes was calculated based on Pascal's constants ($\chi_{Di}$, $\lambda_i$)[6]

$$\chi_D = \sum_i \chi_{D\,i} + \sum_i \lambda_i \qquad (S7)$$

**Complex 3**

*Diamagnetic susceptibility calculation:*

| | $\chi_{Di}$ | $N_i$ | $N_i \times \chi_{D\,i}$ |
|---|---|---|---|
| **$C_{ring}$** | -6.24 | 68 | -424.32 |
| **C** | -6.00 | 8 | -48 |
| **H** | -2.93 | 43 | -125.99 |
| **F** | -6.30 | 24 | -151.2 |
| **$N_{ring}$** | -4.61 | 2 | -9.22 |
| **B** | -7.00 | 1 | -7 |
| **$Bi^{2+*}$** | -25.00 | 1 | -25 |
| | | | |
| | **$\lambda_i$** | | **$N_i \times \lambda_i$** |
| **Benzene** | -1.40 | 11 | -15.4 |
| **C=NR** | 8.15 | 2 | 16.3 |
| **Ar-Ar** | -0.50 | 4 | -2 |
| **$Ar-NR_2$** | 1.00 | 2 | 2 |
| | | $\chi_D$ = | -7.90E-04 $cm^3\ mol^{-1}$ |

*value for $Bi^{3+}$ was used as the value was not reported

*Calculation of $\mu_{eff}$ at different temperatures*

The following constants were used for the calculations:

| | | |
|---|---|---|
| $F_{spectrometer}$ (Hz) | 499870000 | |
| c (mol/L) | 0.0128 | ± 5.00% |
| M (g/mol) | 1659.95 | |
| $\chi_D$ ($cm^3$/mol) | -7.90E-04 | ± 10.00% |
| c (g/L) | 21.22 | |
| ΔT (K) | 1 | |

Table S1. Calculation of magnetic moments ($\mu_{eff}$) of **3** at different temperatures.

| T (K) | $\Delta F_1$ (Hz) | $\Delta F_2$ (Hz) | ΔF (Hz) | | $\chi_m$ (l/g) | $\chi_m$ ($cm^3$/mol) | | $\chi_P$ ($cm^3$/mol) | | $\mu_{eff}$ ($\mu_B$) | |
|---|---|---|---|---|---|---|---|---|---|---|---|
| 213 | 25.22 | 27.09 | 26.16 | ± 1.32 | 5.89E-10 | 9.77E-04 | ± 6.95E-05 | 1.77E-03 | ± 1.05E-04 | 1.74 | ± 0.05 |
| 223 | 23.25 | 24.93 | 24.09 | ± 1.19 | 5.42E-10 | 9.00E-04 | ± 6.32E-05 | 1.69E-03 | ± 1.01E-04 | 1.74 | ± 0.05 |
| 233 | 21.82 | 23.36 | 22.59 | ± 1.09 | 5.08E-10 | 8.44E-04 | ± 5.86E-05 | 1.63E-03 | ± 9.84E-05 | 1.75 | ± 0.05 |
| 243 | 20.46 | 21.83 | 21.15 | ± 0.97 | 4.76E-10 | 7.90E-04 | ± 5.36E-05 | 1.58E-03 | ± 9.54E-05 | 1.75 | ± 0.05 |
| 253 | 19.13 | 20.41 | 19.77 | ± 0.91 | 4.45E-10 | 7.39E-04 | ± 5.01E-05 | 1.53E-03 | ± 9.35E-05 | 1.76 | ± 0.05 |
| 263 | 18.08 | 19.20 | 18.64 | ± 0.79 | 4.20E-10 | 6.96E-04 | ± 4.57E-05 | 1.49E-03 | ± 9.12E-05 | 1.77 | ± 0.05 |
| 273 | 16.95 | 17.98 | 17.47 | ± 0.73 | 3.93E-10 | 6.53E-04 | ± 4.25E-05 | 1.44E-03 | ± 8.97E-05 | 1.77 | ± 0.06 |
| 283 | 15.96 | 16.93 | 16.45 | ± 0.69 | 3.70E-10 | 6.14E-04 | ± 4.00E-05 | 1.40E-03 | ± 8.85E-05 | 1.78 | ± 0.06 |
| 298 | 14.60 | 15.54 | 15.07 | ± 0.66 | 3.39E-10 | 5.63E-04 | ± 3.75E-05 | 1.35E-03 | ± 8.74E-05 | 1.80 | ± 0.06 |

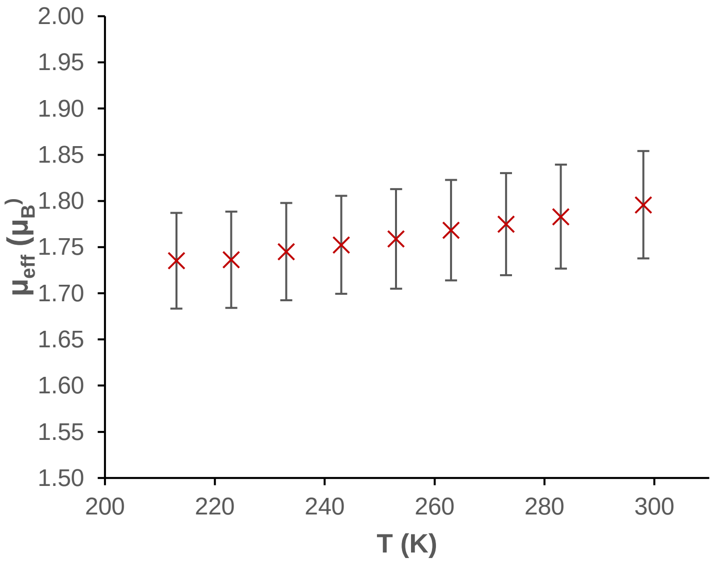


Figure S9. Effective magnetic moments ($\mu_{eff}$) of **3** from 213 K to 298 K.

## Complex 4

*Diamagnetic susceptibility calculation:*

| | $\chi_{Di}$ | $N_i$ | $N_i \times \chi_{D\,i}$ |
|---|---|---|---|
| **$C_{ring}$** | -6.24 | 68 | -424.32 |
| **C** | -6.00 | 10 | -60 |
| **H** | -2.93 | 47 | -137.71 |
| **F** | -6.30 | 24 | -151.2 |
| **$N_{ring}$** | -4.61 | 2 | -9.22 |
| **B** | -7.00 | 1 | -7 |
| **$Bi^{2+}$ *** | -25.00 | 1 | -25 |
| | $\lambda_i$ | | $N_i \times \lambda_i$ |
| **Benzene** | -1.40 | 11 | -15.4 |
| **C=NR** | 8.15 | 2 | 16.3 |
| **Ar-Ar** | -0.50 | 4 | -2 |
| **$Ar\text{-}NR_2$** | 1.00 | 2 | 2 |
| | | $\chi_D$ = | -8.14E-04 $cm^3\ mol^{-1}$ |

*Calculation of $\mu_{eff}$ at different temperatures*

The following constants were used for the calculations:

| | | |
|---|---|---|
| $F_{spectrometer}$ (Hz) | 499870000 | |
| c (mol/L) | 0.0137122 | ± 5.00% |
| M (g/mol) | 1688.00 | |
| $\chi_D$ ($cm^3$/mol) | -8.12E-04 | ± 10.00% |
| c (g/L) | 23.15 | |
| ΔT (K) | 1 | |

Table S2. Calculation of magnetic moments ($\mu_{eff}$) of **4** at different temperatures.

| T (K) | $\Delta F_1$ (Hz) | $\Delta F_2$ (Hz) | ΔF (Hz) | | $\chi_m$ (l/g) | $\chi_m$ ($cm^3$/mol) | | $\chi_P$ ($cm^3$/mol) | | $\mu_{eff}$ ($\mu_B$) | |
|---|---|---|---|---|---|---|---|---|---|---|---|
| 213 | 27.45 | 28.78 | 28.12 | ± 0.94 | 5.80E-10 | 9.79E-04 | ± 5.89E-05 | 1.79E-03 | ± 1.00E-04 | 1.75 | ± 0.05 |
| 223 | 25.56 | 26.75 | 26.16 | ± 0.84 | 5.40E-10 | 9.11E-04 | ± 5.42E-05 | 1.72E-03 | ± 9.77E-05 | 1.75 | ± 0.05 |
| 233 | 23.81 | 24.90 | 24.36 | ± 0.77 | 5.03E-10 | 8.48E-04 | ± 5.02E-05 | 1.66E-03 | ± 9.56E-05 | 1.76 | ± 0.05 |
| 243 | 22.15 | 23.18 | 22.67 | ± 0.73 | 4.68E-10 | 7.89E-04 | ± 4.69E-05 | 1.60E-03 | ± 9.39E-05 | 1.76 | ± 0.05 |
| 253 | 20.75 | 21.63 | 21.19 | ± 0.62 | 4.37E-10 | 7.38E-04 | ± 4.28E-05 | 1.55E-03 | ± 9.19E-05 | 1.77 | ± 0.05 |
| 263 | 19.42 | 20.32 | 19.87 | ± 0.64 | 4.10E-10 | 6.92E-04 | ± 4.11E-05 | 1.51E-03 | ± 9.11E-05 | 1.78 | ± 0.05 |
| 273 | 18.23 | 19.13 | 18.68 | ± 0.64 | 3.85E-10 | 6.51E-04 | ± 3.94E-05 | 1.46E-03 | ± 9.04E-05 | 1.79 | ± 0.06 |
| 283 | 17.39 | 18.19 | 17.79 | ± 0.57 | 3.67E-10 | 6.20E-04 | ± 3.67E-05 | 1.43E-03 | ± 8.93E-05 | 1.80 | ± 0.06 |
| 298 | 15.44 | 16.14 | 15.79 | ± 0.49 | 3.26E-10 | 5.50E-04 | ± 3.25E-05 | 1.36E-03 | ± 8.76E-05 | 1.80 | ± 0.06 |

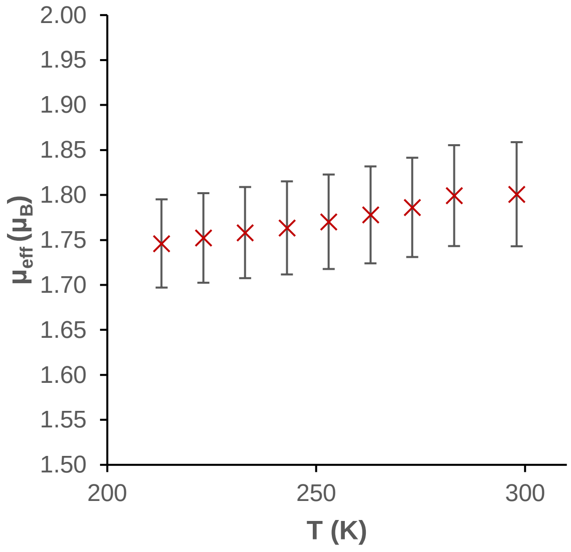


Figure S10. Effective magnetic moments ($\mu_{\mathrm{eff}}$) of **4** from 213 K to 298 K.

# 5 UV-vis

**3** or **4** (1 mg) was dissolved in 1.0 mL toluene. 0.3 mL of this solution was diluted to 0.6 mL (**3**) or used without dilution (**4**).

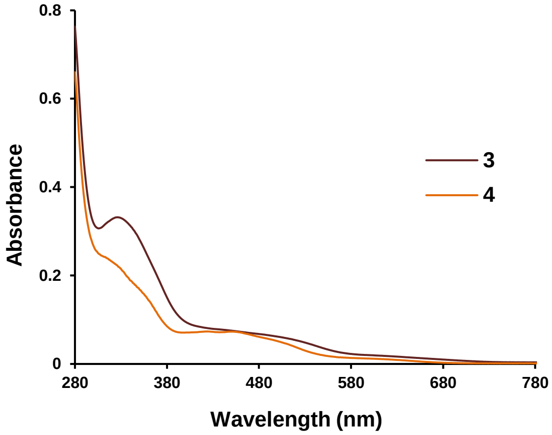


Figure S11. UV-vis spectra of **3** and **4** in toluene at 25 °C.

The UV-vis spectra of **3** at 25 °C showed a broad absorption band from 580 to 400 nm and an intense absorption band at 330 nm (Figure S11). A TD-DFT calculation of the optimized structure of **3** showed intense transitions at 330 and 334 nm. The absorption at 330 nm was assigned as the HOMO-3 and HOMO-4 to HOMO (π(C-C)→6*p*(Bi)), as well as HOMO-1 to LUMO+3 (π(C-C)→π*(C-C)) (*vide infra*). The absorption at 334 nm was assigned as HOMO-4 to HOMO (π(C-C)→6*p*(Bi)) and HOMO-2 to LUMO (π(C-C)→π*(C-C) on the back bond). The UV-vis spectra of **4** at room temperature showed a broad absorption band from 550 to 400 nm, accompanied by two shoulders at 360 and 330 nm (Figure S11). A TD-DFT calculation of the optimized structure of **4** showed strong transitions at 332 and 356 nm, and medium to weak transitions from 500 nm to 400 nm (maximums at 400 and 410 nm). The absorption bands at 330 and 356 nm were both assigned as the aromatic system of N substituents (HOMO-3, HOMO-2) to the HOMO of **4** (π(C-C) → 6*p*(Bi)). The absorptions at 424, 482 and 499 nm are comprised of ligand to ligand transitions.

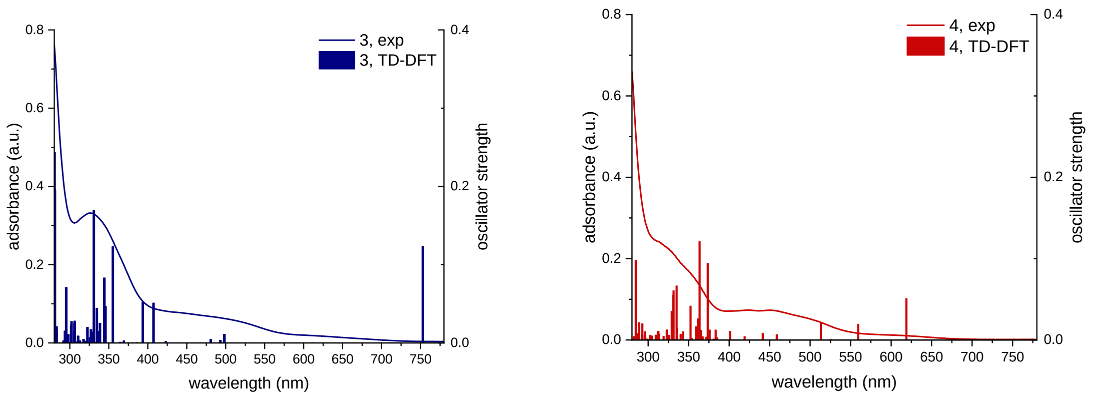


Figure S12. Overlap of computed and experimental absorption spectra in toluene for **3** (left) and **4** (right).

# 6 IR

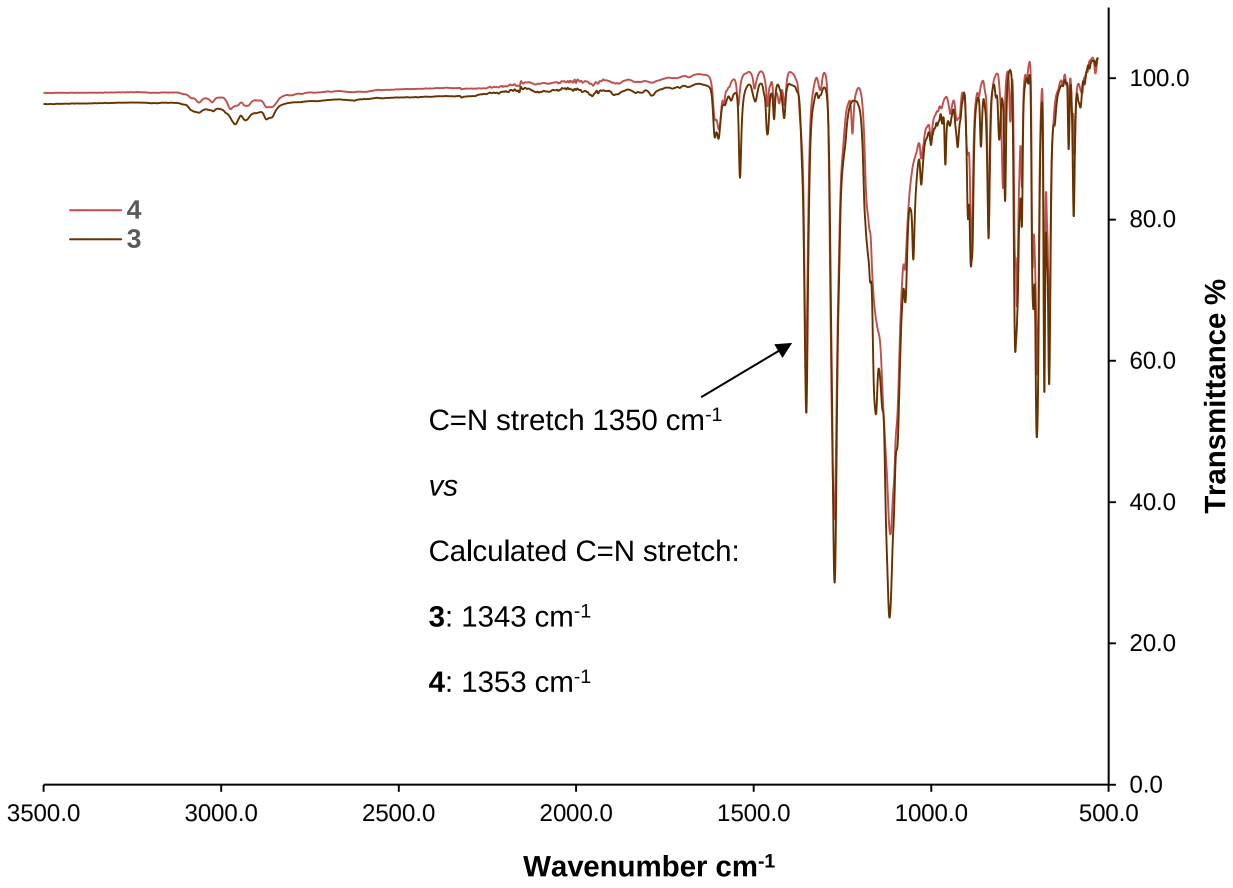


Figure S13. Solid-state IR spectra of complexes **3** and **4**.

# 7 Cyclic Voltammetry

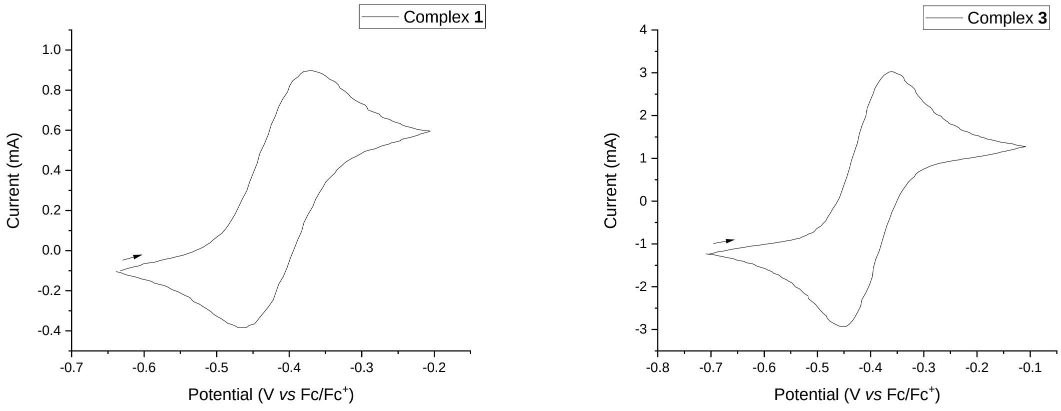


Figure S14. Cyclic voltammetry of complex **1** (left) and **3** (right) in 0.1 M solution of $[nBu_4N][BAr^F]$ in $CH_3CN$ *vs*. $Fc/Fc^+$ at a scan rate of 100 mV/s; working electrode: glassy carbon; counter electrode: Pt; reference electrode: Ag.

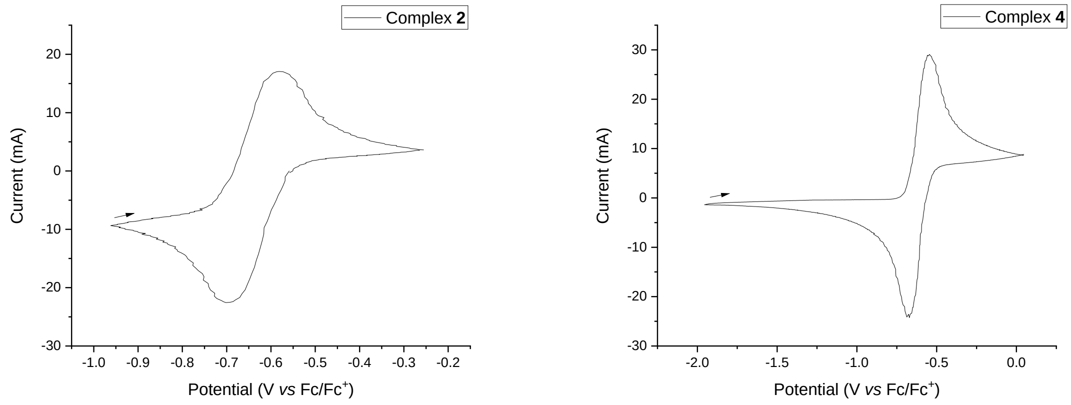


Figure S15. Cyclic voltammetry of complex **2** (left) and **4** (right) in 0.1 M solution of [nBu$_4$N][BAr$^F$] in THF *vs*. Fc/Fc$^+$ at a scan rate of 100 mV/s; working electrode: glassy carbon; counter electrode: Pt; reference electrode: Ag.

# 8 EPR

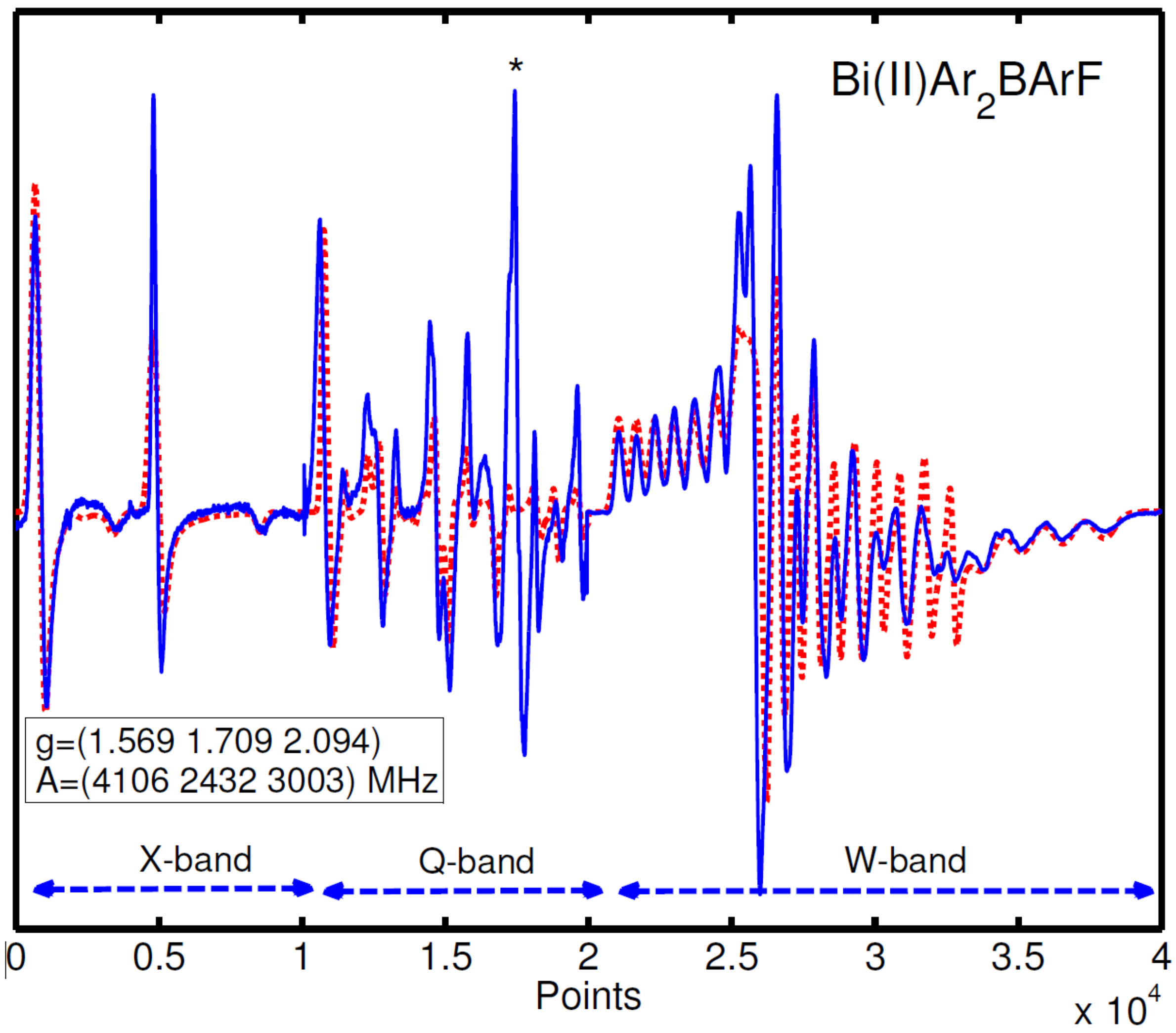


Figure S16. Combined X-, Q-, and W-band spectral features of **3** (solid blue trace) and the corresponding spectral fit (dashed red trace) using the magnetic parameters as listed in Table 2. The (*) marked feature at Q-band originates from an unknown impurity around g~2. Note that

the X, Q, and W-EPR spectra are concatenated during the fitting procedure. Therefore, the X-axis indicates the range of data points (total of 40000).

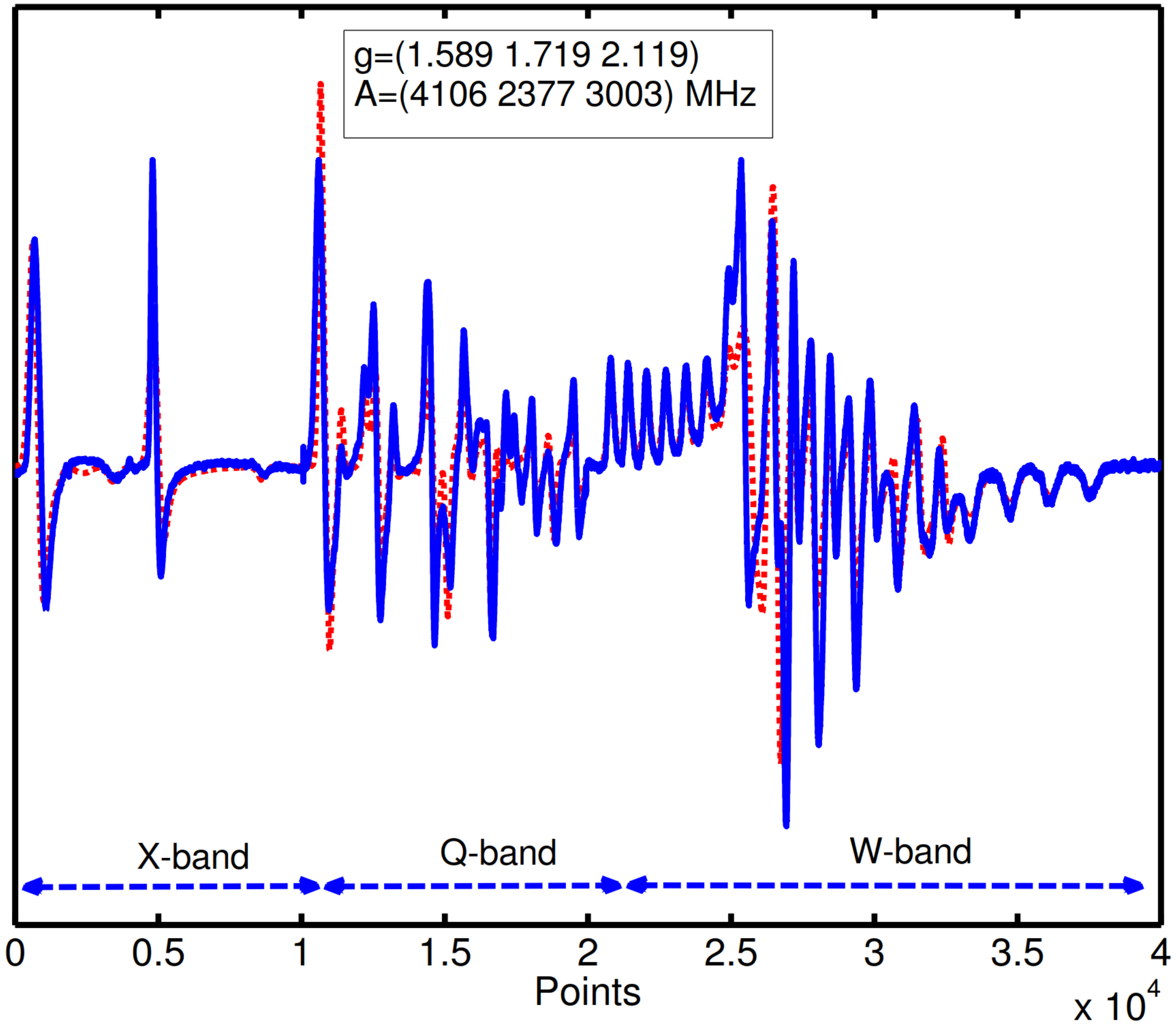


Figure S17. Combined X-, Q-, and W-band spectral features of **4** (solid blue trace) and the corresponding spectral fit (dashed red trace) using the magnetic parameters as listed in Table 2.

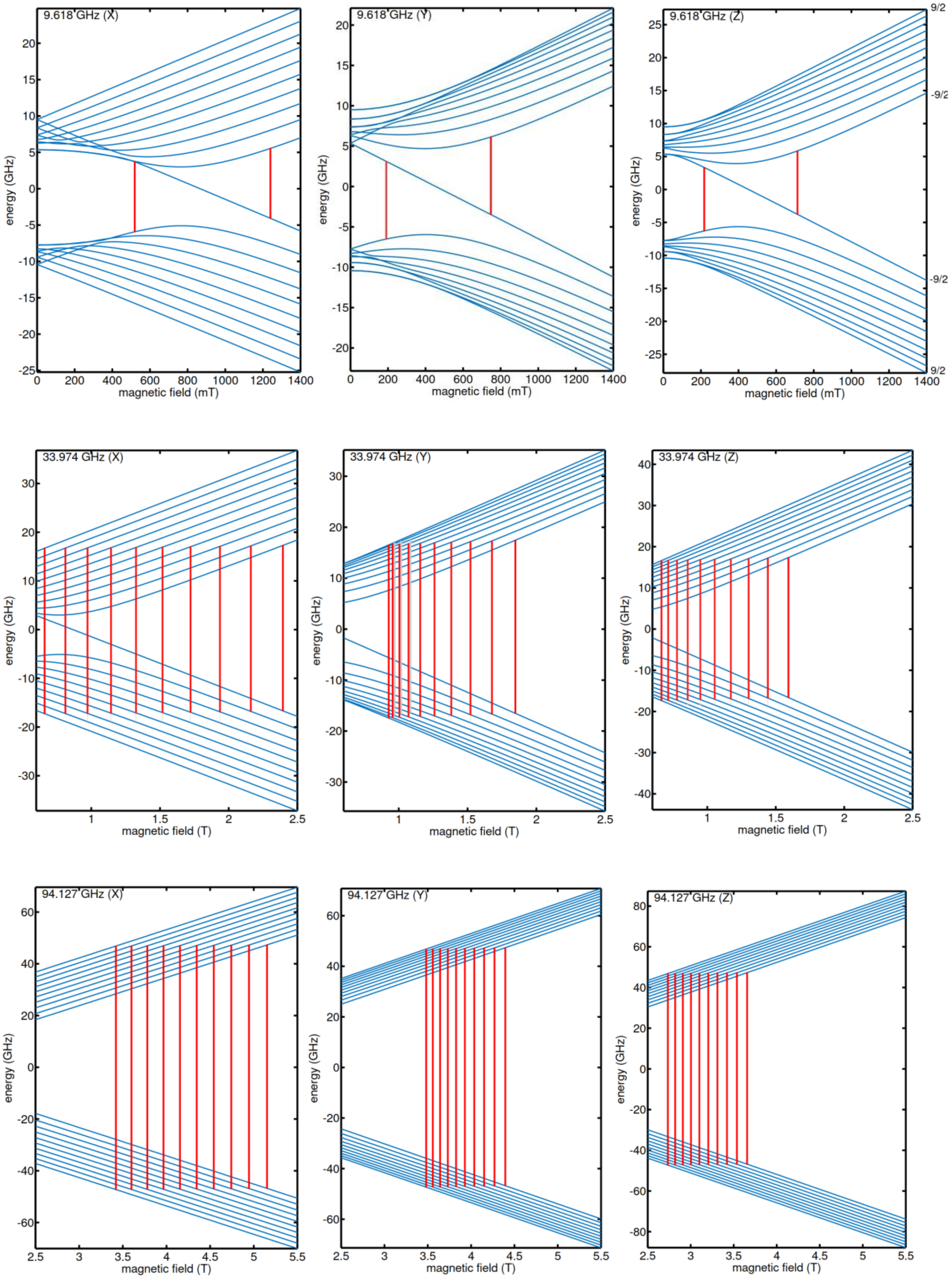

9.618 GHz (X)
9.618 GHz (Y)
9.618 GHz (Z)
33.974 GHz (X)
33.974 GHz (Y)
33.974 GHz (Z)
94.127 GHz (X)
94.127 GHz (Y)
94.127 GHz (Z)
energy (GHz)
magnetic field (mT)
magnetic field (T)
9/2
-9/2
-9/2
9/2

Figure S18. Energy levels and EPR transitions at X-, Q-, and W-band for **4** corresponding to the spin Hamiltonian parameters given in Table 2 (see also Figure S17).

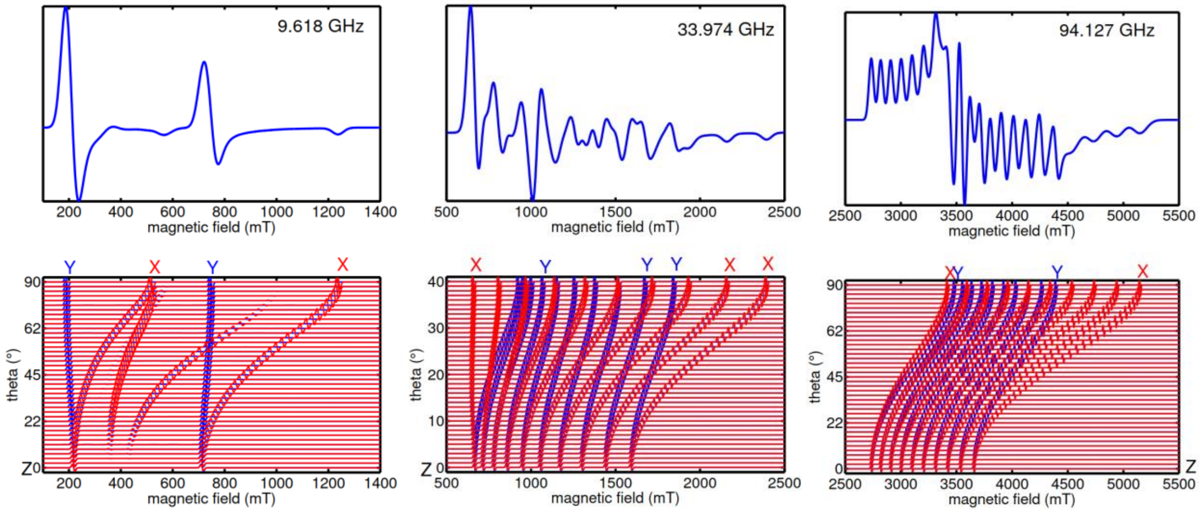


Figure S19. Powder Simulations at X-(1st derivative), Q-(1st derivative), and W-band (1st derivative) corresponding to the spin Hamiltonian parameters fitted for **4** (Table 2 and Figure S17). The lower panels show the corresponding orientation dependent single crystal spectra (roadmap).

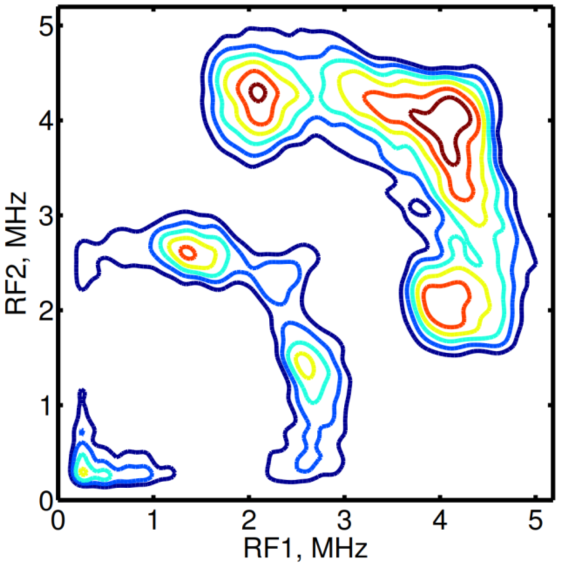


Figure S20. Q-band HYSCORE spectrum at 663 mT of **3**. Experimental parameters: t90=20 ns, τ=324 ns, 7 K.

Table S3. *g*-tensor and hyperfine-tensor (MHz) parameters of Bi(II) Compounds as well as the Bi(II) spin populations (*P*) according to spectral simulation and DFT calculations. Estimated

“Experimental” spin populations of the *s*-orbital *P*(s) and *p*-orbital *P*(p) are calculated from the HFI according to the procedure of Morton and Preston[7] while the “theoretical” (Mulliken) spin populations are obtained directly from the molecular orbitals as calculated with DFT.

| Compound | $g_x$ | $g_y$ | $g_z$ | $A_x$ | $A_y$ | $A_z$ | *P*(*s*) | *P*(*p*) | *P* |
|---|---|---|---|---|---|---|---|---|---|
| **3** | 1.569 | 1.709 | 2.094 | 4106 | 2432 | 3003 | 0.048 | 0.816 | 0.86 |
| **4** | 1.589 | 1.719 | 2.119 | 4106 | 2377 | 3003 | 0.048 | 0.832 | 0.88 |
| DFT of **3,4** | | | | | | | | | |
| **3-**B3LYP | 1.538 | 1.994 | 2.159 | -8618 | -2902 | -6788 | | | 0.87 |
| **3-**PBE0 | 1.582 | 1.995 | 2.173 | -100091 | -4446 | -8338 | | | 0.92 |
| **3-**TPSSh | 1.634 | 1.997 | 2.180 | -9817 | -4532 | -8231 | | | 0.89 |
| **4-**B3LYP | 1.579 | 1.994 | 2.195 | -8701 | -2972 | -6916 | | | 0.89 |
| | | | | | | | | | |
| $O(SiN)_2Bi$[8] | 1.621 | 1.676 | 1.832 | 4764 | 2804 | 3830 | 0.057 | 0.851 | 0.91 |
| | | | | | | | | | |
| $[LXGa]_2Bi$[9] | 1.54 | 1.95 | 2.67 | 1360 | 2140 | 1450 | 0.025 | 0.426 | 0.45 |
| [LClGaBi][10] | 1.73 | 2.09 | 2.61 | 900 | 2050 | 1200 | 0.021 | 0.588 | 0.61 |

An experimental estimate of the bismuth spin population *P*(Bi) can be made by separating the isotropic component of the HFI (attributed to the *s*-orbital spin population) from the anisotropic component (attributed to the *p*-orbital spin population), i.e. $A_{iso} = (A_1+A_2+A_3)/3$ and $A_{aniso} = (A_1, A_2, A_3) - A_{iso}$. According to the approach of Morton and Preston[7] the *s*-population can be calculated as the ratio of the $A_{iso}$ to the tabulated intrinsic $A_{iso}$ of Bi with 100% *s*-population (66195 MHz) while the *p*-population is obtained as the ratio of the axial component $T = ((A_2+A_3)-A_{iso})/2$ to the intrinsic *T*-value of Bi in case of 100% p-population (567 MHz). These estimated Bi-spin populations are presented in Table 2 for **3** and **4**.

# 9 Computational Details

All quantum chemical calculations in the present work were performed using the ORCA 5.0.3 program suite employing the scalar relativistic zeroth order regular approximation (ZORA).[11] Geometry optimizations were carried out using the BP86 density functional. TD-DFT calculations were carried out using the PBE0 density functional, while EPR analyses were carried out using the B3LYP density functional. All calculations are in conjunction with the ZORA-Def2-TZVP basis set for hydrogen (H), carbon (C), nitrogen (N), and oxygen (O). For Bismuth (Bi), the SARC-ZORA-TZVP basis set was used which features a contraction optimized for the ZORA Hamiltonian. The RI approximation with the SARC/J fitting basis set was employed to accelerate the calculations. Furthermore, the atom-pairwise D3 dispersion correction with Becke-Johnson (D3BJ) damping was taken into account. Subsequent frequency calculations revealed that all optimized geometries are local minima having no imaginary frequencies.

Table S4. Comparison of the optimized geometry of **3** from different methods with the experimental crystal structure.

| | **3** | BP86 | B3LYP | PBE0 |
|---|---|---|---|---|
| Bi–N1 | 2.423(3) | 2.487 | 2.490 | 2.470 |
| Bi–N2 | 2.505(3) | 2.478 | 2.500 | 2.468 |
| Bi–C1 | 2.177(3) | 2.198 | 2.198 | 2.182 |
| N1–C7 | 1.280(5) | 1.299 | 1.284 | 1.282 |
| N2–C8 | 1.284(4) | 1.299 | 1.284 | 1.282 |
| C1-Bi-N1 | 70.357 | 70.947 | 70.832 | 70.892 |
| C1-Bi-N2 | 72.060 | 71.045 | 70.750 | 70.941 |

Table S5. Comparison of the optimized geometry of **4** from different methods with the experimental crystal structure.

| | **4** | BP86 | B3LYP | PBE0 |
|---|---|---|---|---|
| Bi–N1 | 2.4569(18) | 2.448 | 2.455 | 2.438 |
| Bi–N2 | 2.4786(17) | 2.446 | 2.459 | 2.440 |
| Bi–C1 | 2.1809(19) | 2.196 | 2.195 | 2.179 |
| C1-Bi-N1 | 71.320 | 71.071 | 70.951 | 71.032 |
| C1-Bi-N2 | 71.477 | 71.130 | 70.991 | 71.079 |

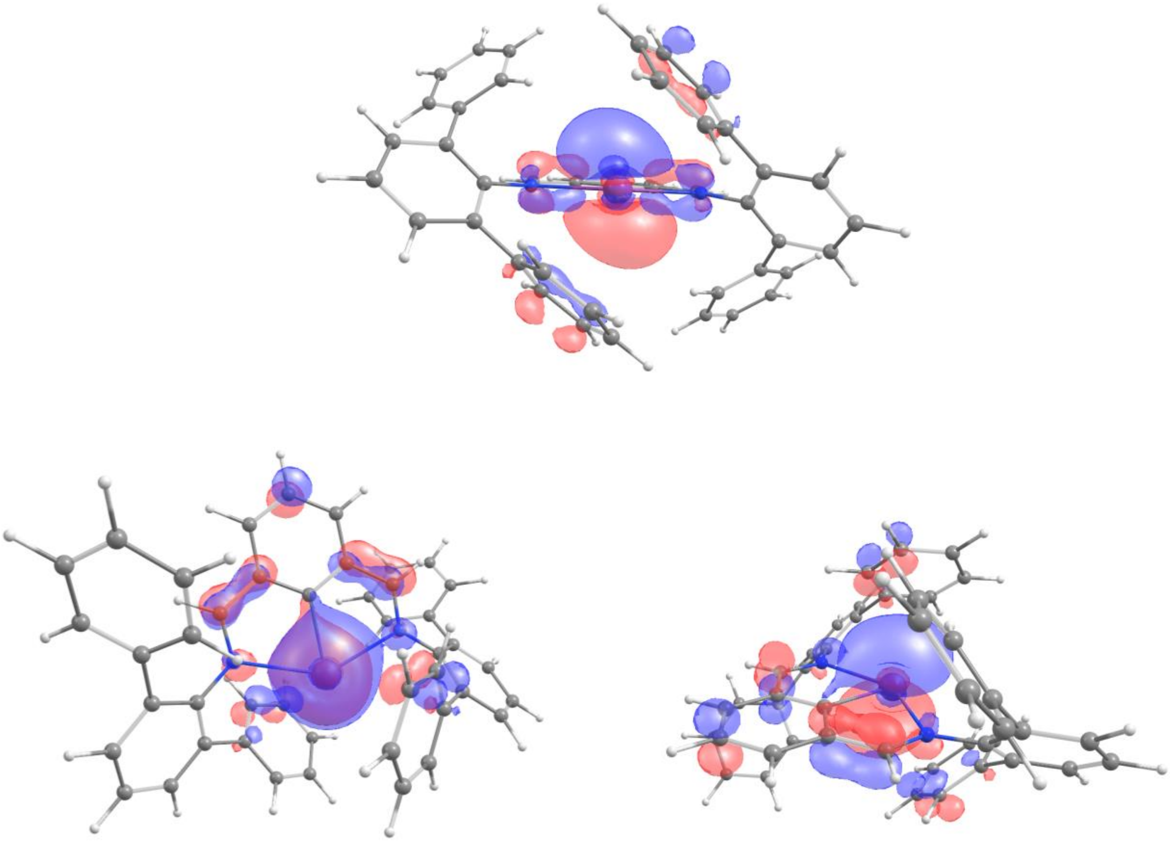

Figure S21. HOMO orbital of **3**.

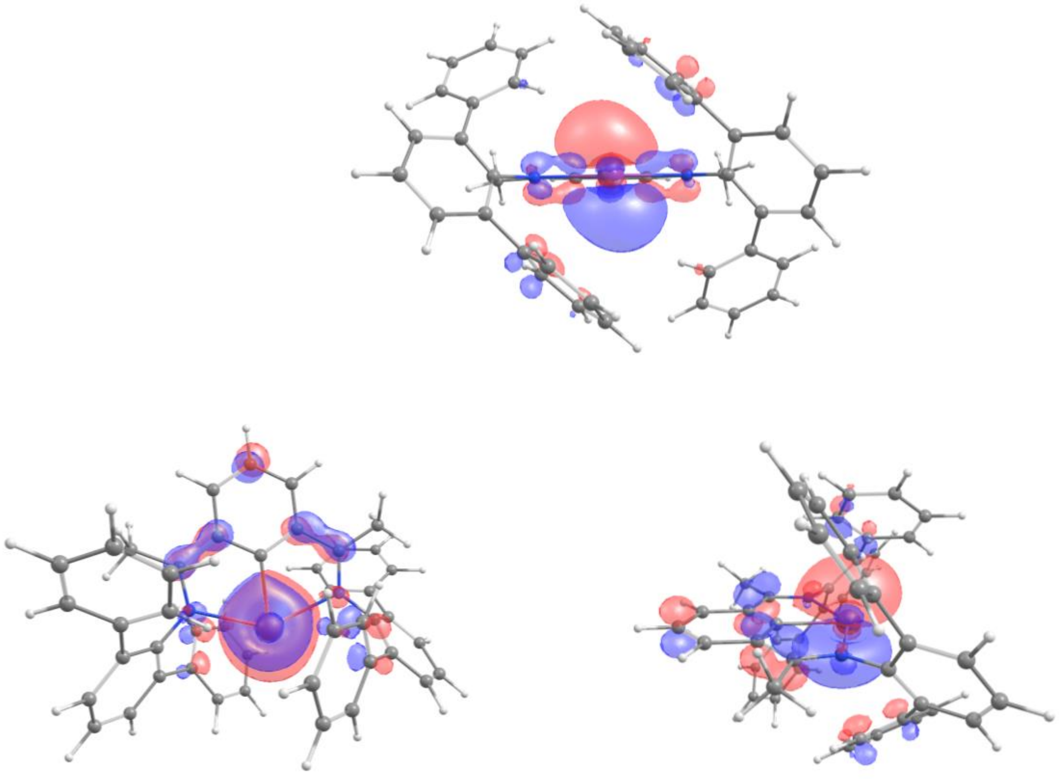

Figure S22. HOMO orbital of **4**.

Table S6. Spin populations of Bi, N1, and C8.

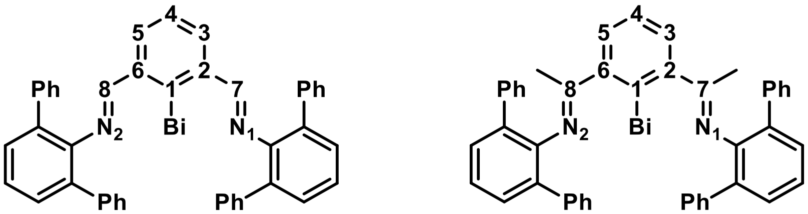


| | Mulliken spin populations | |
|---|---|---|
| Atom | **3** | **4** |
| Bi | 0.836 | 0.887 |
| N1 | -0.01 | -0.023 |
| N2 | -0.01 | -0.024 |
| C1 | -0.079 | -0.08 |

| | | |
|---|---|---|
| C2 | 0.049 | 0.045 |
| C4 | 0.056 | 0.052 |
| C6 | 0.049 | 0.045 |
| C7 | 0.053 | 0.055 |
| C8 | 0.058 | 0.056 |

# 10 SQUID

Variable-temperature DC susceptibility data for **3** and **4** were measured with a MPMS 3 Quantum Design SQUID magnetometer at the Max-Planck-Institut für Chemische Energiekonversion (MPI CEC) in the Joint Workspace of the Max-Planck-Institut für Kohlenforschung. Susceptibility data were obtained by cooling to 2.0 K with no applied external magnetic field and subsequently measuring the magnetic moment from 2.0 to 300 K in an applied 1000 G DC magnetic field. The data for **4** exhibit an outlier at the 300 K datapoint, which was therefore excluded from modeling. The data for **3** exhibit an unexpected deviation in the 2.0-6.8 K temperature range, likely due to an impurity exhibiting antiferromagnetic ordering; therefore, the 2.0-6.8 K data were excluded from the modeling. Experimental susceptibility data were corrected for underlying diamagnetism using the following equation: $\chi_{\mathrm{D}} = -(M_W/2) \cdot 10^{-6}$ $cm^3$ $mol^{-1}$.[6] The susceptibility and magnetization data for both compounds were modeled simultaneously using JulX20.[12] All data were parameterized using the following Zeeman spin Hamiltonian:

$$\hat{H} = \beta \vec{B} \bar{\bar{g}} \vec{\hat{S}}$$

where $\beta$ is the Bohr magneton, $\vec{B}$ is the applied external magnetic field, and $\vec{\hat{S}}$ and $\bar{\bar{g}}$ are the spin vector and electron $g$-tensor, respectively. Both compounds were modeled with average$g$-values ($g_x = g_y = g_z$). Temperature-independent paramagnetism (TIP) was also included in the models of the susceptibility data. Furthermore, a Weiss constant ($2zJ$) was included in the model for **4** to account for intermolecular spin-spin interactions, the cause of the small downturn in the data at 4.1 K.

Table S7. Fit parameters for **3** and **4**.

| | **3** | **4** |
|---|---|---|
| $g_{\mathrm{avg}}$ | 1.60(5) | 1.72(5) |
| $2zJ$ ($cm^{-1}$) | ---- | –24.3 |
| TIP ($cm^3$ $mol^{-1}$) | 0.0008043 | 0.0003272 |
| Residual | 0.00923 | 0.00677 |

# 11 XRD

## 11.1 Structure search in the Cambridge Structural Database (CSD)

A database (WebCSD Version 1.2.42; https://www.ccdc.cam.ac.uk/structures/WebCSD/StructureSearch ) survey was performed to search for related structural motifs. The following search motif was used in the web interface structure search with ‘substructure’ match conditions:

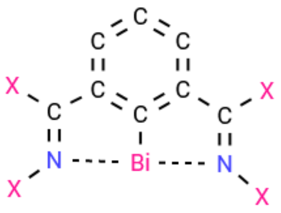


The initial search found 28 structures containing the predefined Bi:N,C,N pincer entity. All entries were listed in the following table. Herein Bi mainly appears in oxidation state +I and +III.

Table S8. Overview of search results for Bi:N,C,N pincer structures.

| Database identifier | Deposition number | Database identifier | Deposition number |
|---|---|---|---|
| ADUTAX | 1553198 | OMIYEQ | 1436527 |
| CURBAT | 771564 | UZOCOD | 1496624 |
| GELPEW | 2149908 | UZODEU | 1496627 |
| GELSAV | 2097218 | VUCMUD | 1032176 |
| IBEBIE | 2091964 | VUSVIQ | 1409228 |
| IBEBOK | 2091965 | VUSWAJ | 1409231 |
| IBEBUQ | 2091966 | VUSWEN | 1409232 |
| IBECAX | 2091967 | WUXHOP | 1990140 |
| IBECEB | 2091968 | WUXJEH | 1990144 |
| KADXEW | 2031445 | WUXJIL | 1990145 |
| KADXIA | 2031446 | XANTOY | 1515225 |
| KADXOG | 2031447 | XANVEQ | 1515228 |
| KADXUM | 2031448 | XANVOA | 1515230 |
| OMIYAM | 1436526 | XANVUG | 1515231 |

## 11.2 Structural discussions and comparison

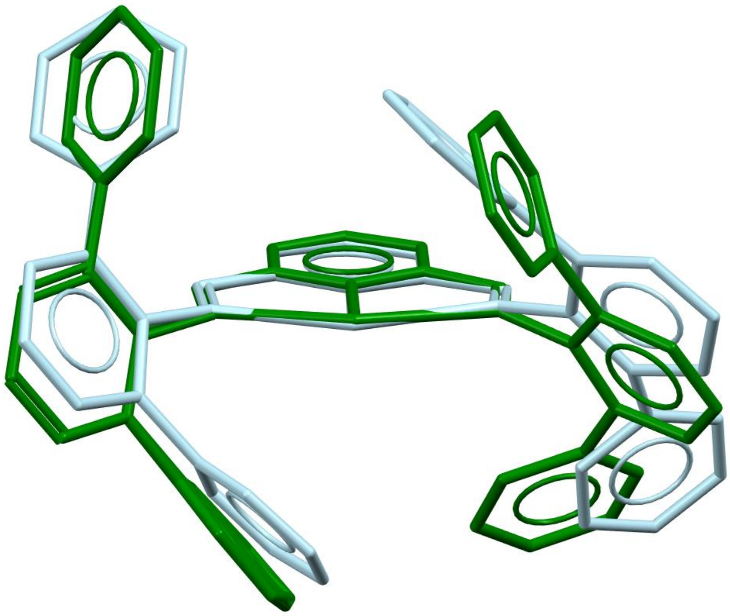

Figure S23. Overlay of the Bi:N,C,N pincer units of cationic part of **3** (light blue) and **1** (green) showing a RMS of 0.0617. Only the *meta*-Terphenyl units show different conformations in both structures.

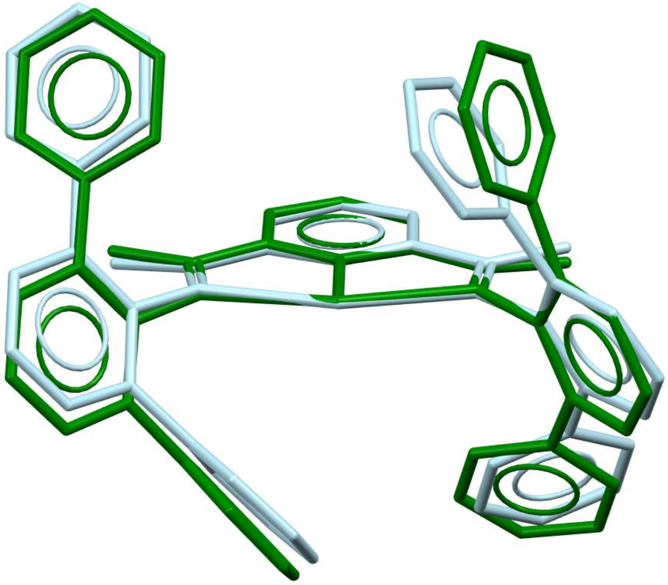

Figure S24. Overlay of the Bi:N,C,N pincer units of cationic part of **4** (light blue) and **2** (green) showing a RMS of 0.0626. Only the *meta*-Terphenyl units show different conformations in both structures.

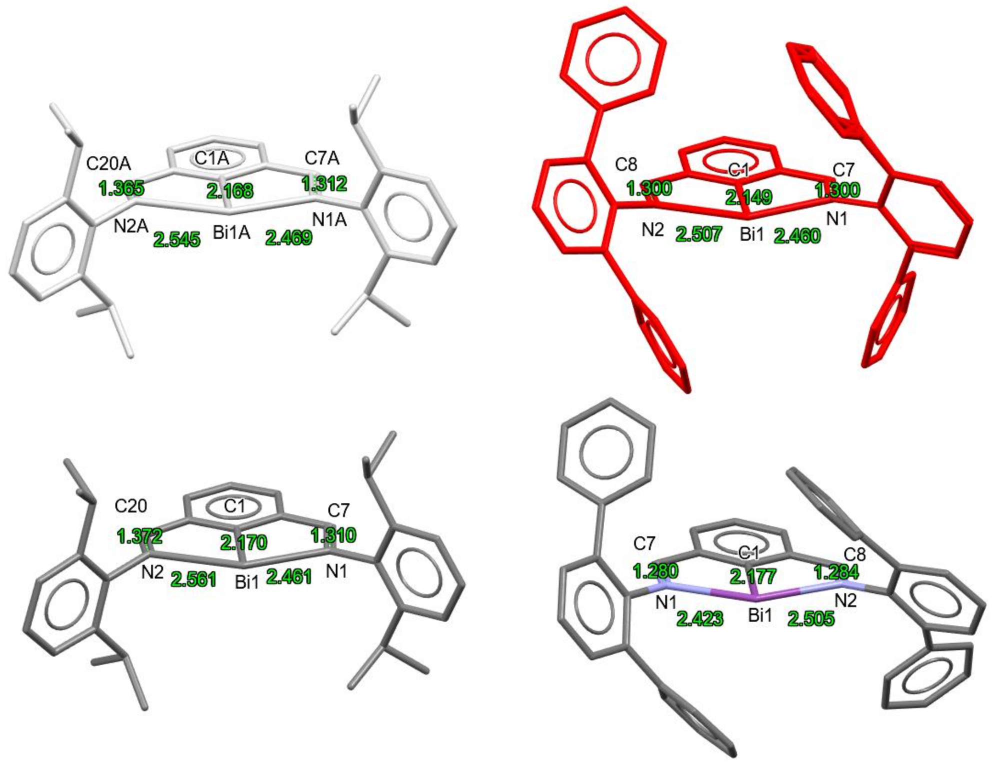


Figure S25. Comparison of selected bond length of cationic part of **3** (colored by elements), **1** (red) and both molecules of the asymmetric unit of **GELPEW** (grey).

By comparing selected bond length of **3**, **1** and **GELPEW**, one could observe that the cationic Bi(II) structure of **3** shows significant differences in the Bi–C (longest) and N=C (shortest) bond lengths. An inspection of corresponding angles does not show any significant differences. A similar trend can be observed in the methylated structures of **2**, **4** and **CURBAT**.

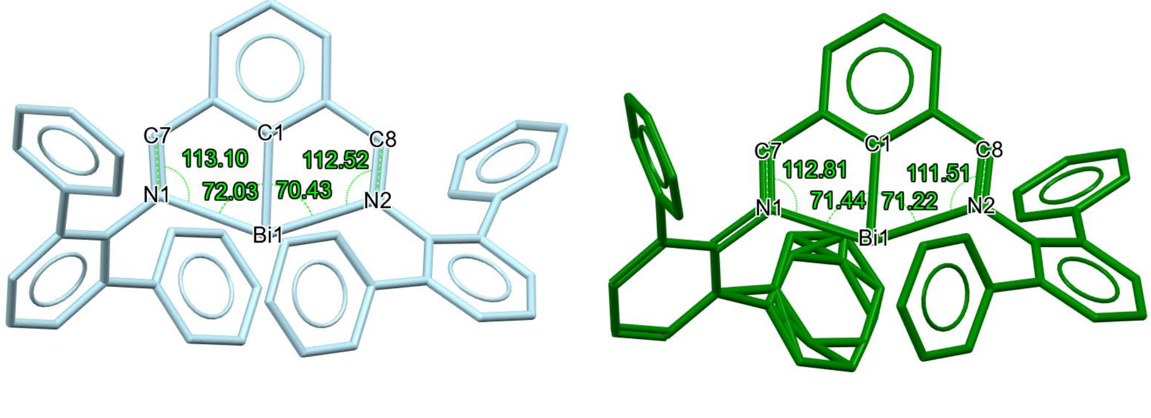


Figure S26. Comparison of selected angles of cationic part of **3** (light blue) and **1** (green).

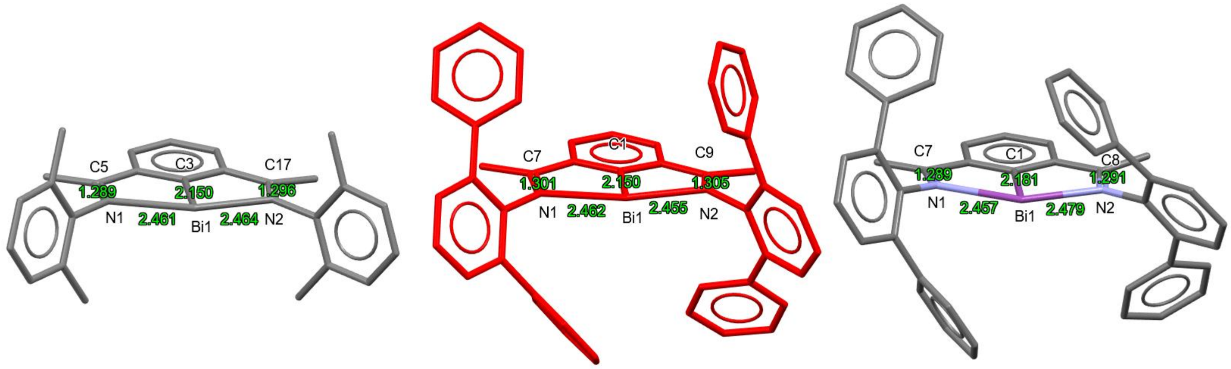


Figure S27. Comparison of selected bond length of CURBAT (grey), **2** (red) and **4** (colored by elements).

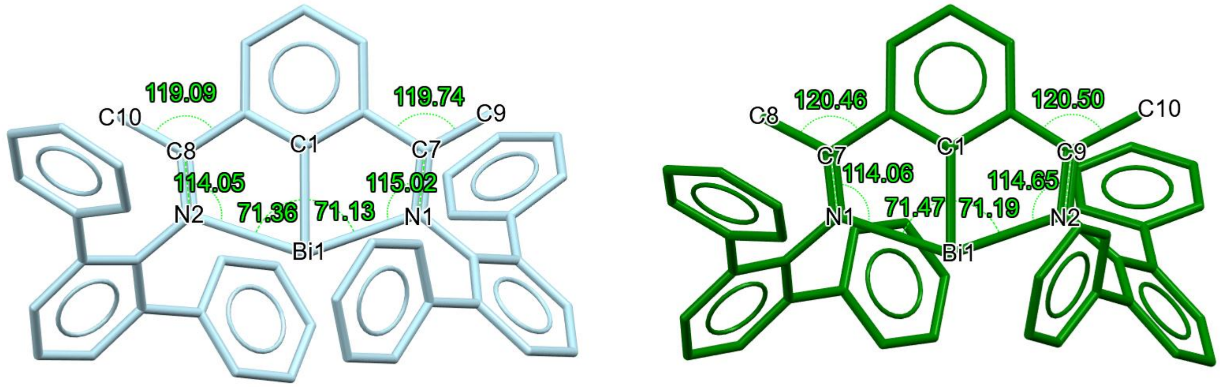


Figure S28. Comparison of selected angles of **2** (light blue) and **4** (green).

## 11.3 Intermolecular interactions

The structures of **3** and **4** have been inspected for short contacts. Using the short contact function of Mercury program (Version 2021.2.0, Short contact < sum of van der Waals radii) reveals differences in both structures. It is worth noting, that in **3** a short contact (3.329 Å) between the positively charged Bi(II) atom and one F atom of a $BAr^F$ anion can be found. The compound was crystallized from a solvent mixture of 1,4-dioxane and pentane.

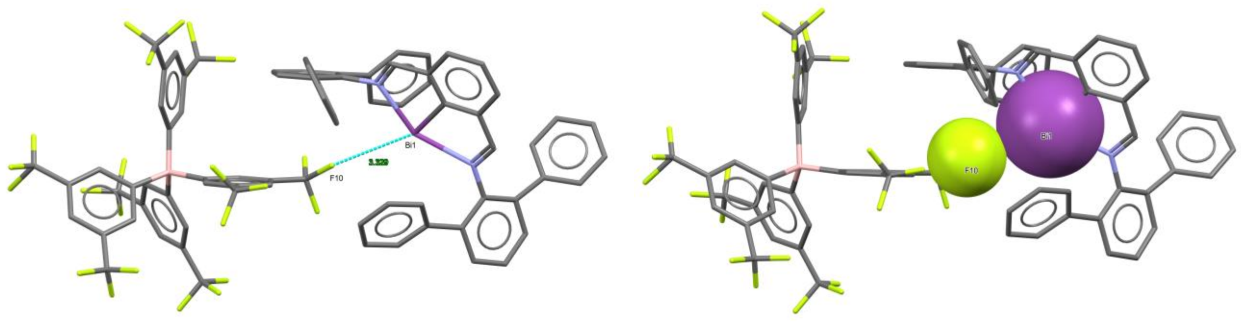


Figure S29. Short contacts in **3** indicated by blue dashed line (left) and by van der Waals representation of both interacting atoms (right).

The intermolecular interactions in structure **4** are different. Here one can observe a short contact (3.341 Å) between the positively charged Bi(II) atom and one F atom of a 1,2-difluorobenzene solute molecule. The small and flat solute molecule is able to insert into the pocket formed from the *meta*-Terphenyl substituted N,C,N pincer ligand. This occurs in spite of the presence of the F atoms in the $BAr^F$ anion. It is worth noting, that the closest contacts to F atoms of the surrounding $BAr^F$ anions range between 5.152 and 6.026 Å. This is presumably an indicator of a highly fluorophilic behavior of Bi(II) cations.

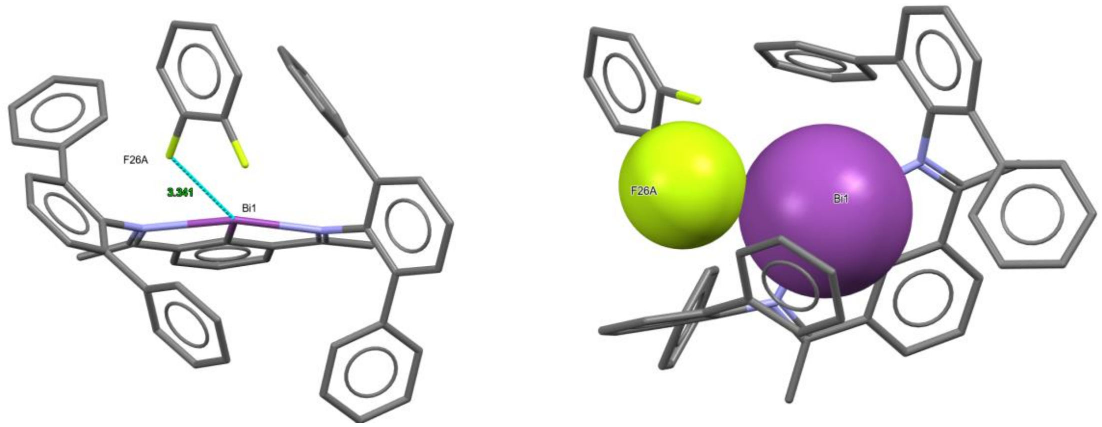


Figure S30. Short contacts in **4** indicated by blue dashed line (left) and by van der Waals representation of both interacting atoms (right).

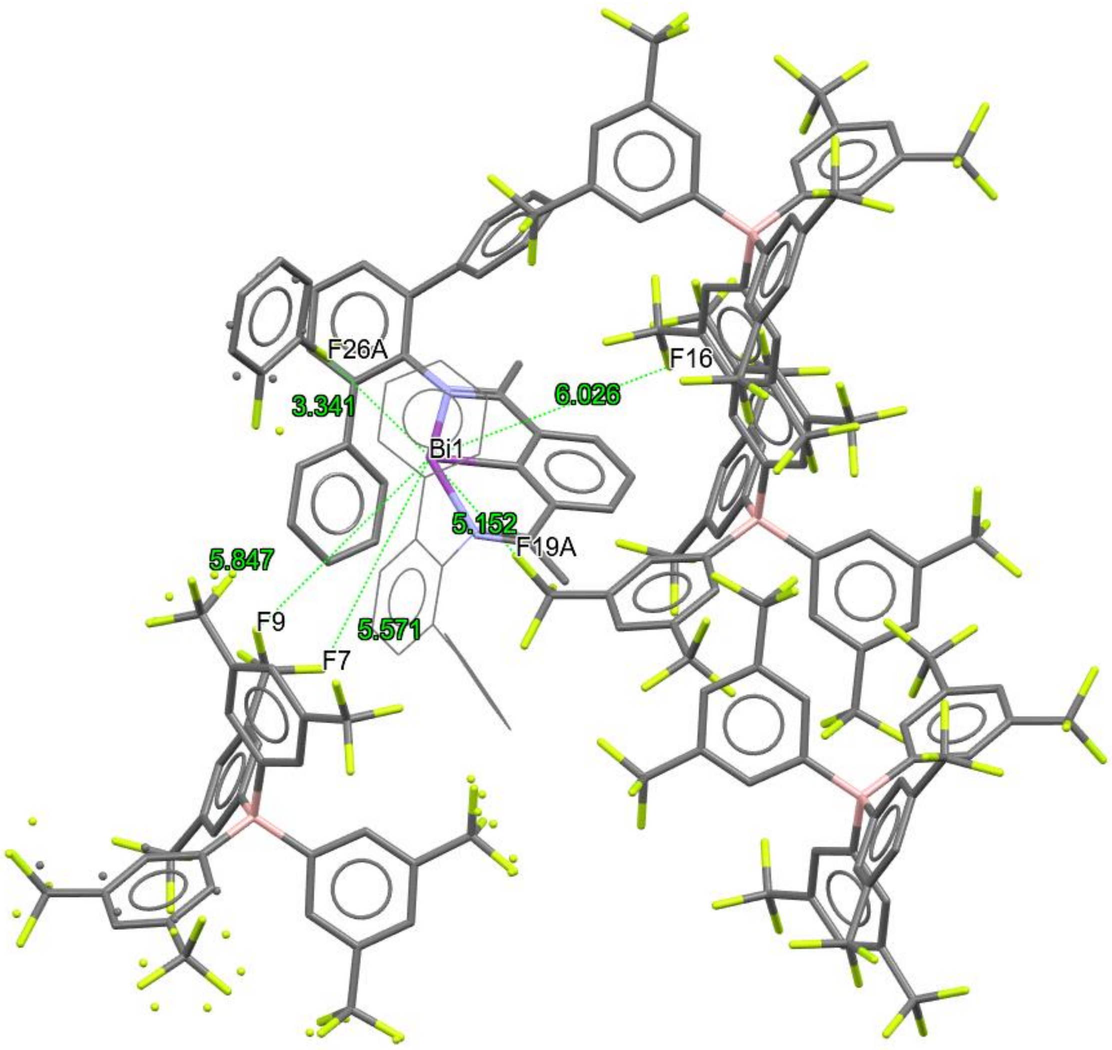


Figure S31. Comparison of various Bi … F distances in the structure of **4**.

## 11.4 Single crystal structure analysis of compound **3**·THF·pentane solvate

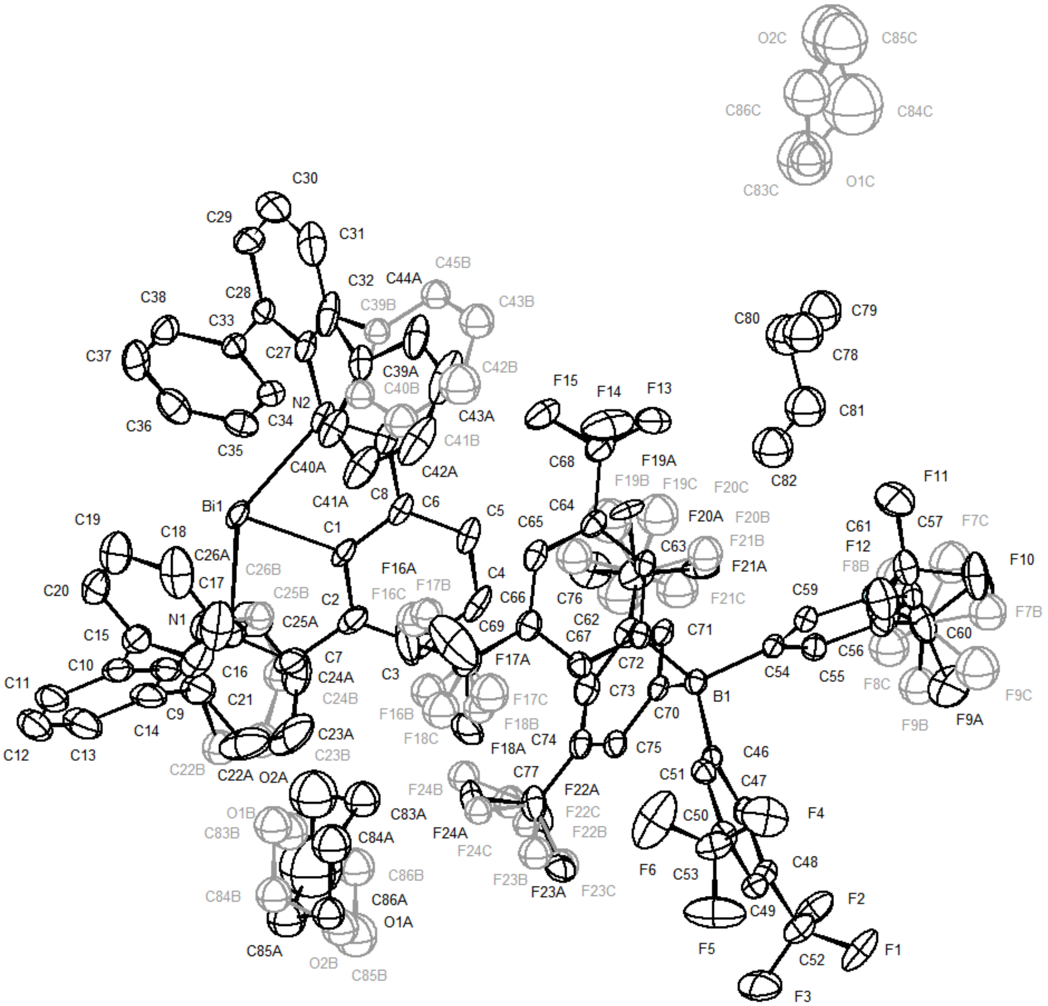


Figure S32. The molecular structure of **3**·THF·pentane solvate. H atoms have been removed for clarity. Main structure shown in black and disordered parts shown in grey.

**X-ray Crystal Structure Analysis of compound 3·THF·pentane solvate**:

$C_{165}H_{114}B_2Bi_2F_{48}N_4O_4$, $M_r$ = 3568.18 g mol$^{-1}$, orange plate, crystal size 0.314 x 0.142 x 0.02 mm$^3$, triclinic, space group *P*-1 [2], *a* = 13.3187(6) Å, b = 17.2111(7) Å, *c* = 18.5792(9) Å, α= 102.770(2)°, β= 100.007(2)°, γ = 110.397(2), *V* = 3743.7(3) Å$^3$, *T* = 100(2) K, *Z* = 1, $D_{calc}$ = 1.583 g·cm$^3$, $\lambda$ = 0.71073 Å, $\mu(Mo\text{-}K_\alpha)$ = 2.464 mm$^{-1}$, Gaussian absorption correction ($T_{min}$ = 0.48561, $T_{max}$ = 0.91621), Bruker-AXS Kappa Mach3 with APEX-II detector and IμS microfocus Mo-anode X-ray source, 1.169 < $\theta$ < 29.130°, 114687 measured reflections, 20167 independent reflections, 16174 reflections with $I > 2\sigma(I)$, $R_{int}$ = 0.0570. The structure was solved by *SHELXT* and refined by full-matrix least-squares (*SHELXL*) against $F^2$ to $R_1$ = 0.0411

[$I > 2\sigma(I)$], $wR_2$ = 0.0917 [all data], 1141 parameters and 80 restraints.

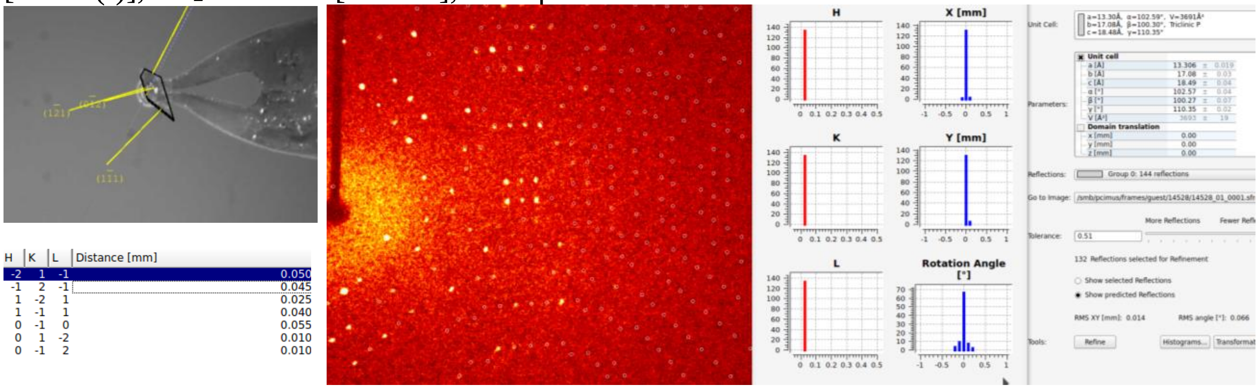


Figure S33. Crystal faces and unit cell determination/refinement of compound **3**·THF·pentane solvate.

INTENSITY STATISTICS FOR DATASET

| Resolution | #Data | #Theory | %Complete | Redundancy | Mean I | Mean I/s | Rmerge | Rsigma |
|---|---|---|---|---|---|---|---|---|
| Inf - 2.81 | 359 | 359 | 100.0 | 9.40 | 103.08 | 74.18 | 0.0226 | 0.0110 |
| 2.81 - 1.86 | 848 | 848 | 100.0 | 9.54 | 56.24 | 60.94 | 0.0265 | 0.0128 |
| 1.86 - 1.48 | 1203 | 1203 | 100.0 | 9.49 | 37.46 | 49.47 | 0.0328 | 0.0159 |
| 1.48 - 1.29 | 1248 | 1248 | 100.0 | 9.32 | 26.62 | 40.47 | 0.0431 | 0.0201 |
| 1.29 - 1.17 | 1237 | 1237 | 100.0 | 9.06 | 19.94 | 31.33 | 0.0548 | 0.0256 |
| 1.17 - 1.09 | 1153 | 1153 | 100.0 | 8.35 | 17.01 | 25.79 | 0.0713 | 0.0316 |
| 1.09 - 1.02 | 1338 | 1338 | 100.0 | 6.47 | 14.68 | 19.52 | 0.0839 | 0.0424 |
| 1.02 - 0.97 | 1212 | 1212 | 100.0 | 5.39 | 12.18 | 15.36 | 0.0955 | 0.0551 |
| 0.97 - 0.93 | 1137 | 1137 | 100.0 | 4.78 | 9.97 | 12.14 | 0.1112 | 0.0703 |
| 0.93 - 0.89 | 1371 | 1371 | 100.0 | 4.31 | 8.56 | 10.07 | 0.1257 | 0.0863 |
| 0.89 - 0.86 | 1229 | 1229 | 100.0 | 4.00 | 7.37 | 8.41 | 0.1322 | 0.1045 |
| 0.86 - 0.84 | 885 | 885 | 100.0 | 3.86 | 6.41 | 7.26 | 0.1553 | 0.1225 |
| 0.84 - 0.81 | 1539 | 1539 | 100.0 | 3.74 | 5.56 | 6.14 | 0.1795 | 0.1459 |
| 0.81 - 0.79 | 1148 | 1148 | 100.0 | 3.65 | 5.00 | 5.44 | 0.1881 | 0.1681 |
| 0.79 - 0.77 | 1258 | 1260 | 99.8 | 3.48 | 4.46 | 4.72 | 0.2139 | 0.1967 |
| 0.77 - 0.75 | 1407 | 1408 | 99.9 | 3.45 | 3.90 | 4.02 | 0.2569 | 0.2301 |
| 0.75 - 0.74 | 747 | 748 | 99.9 | 3.35 | 3.45 | 3.45 | 0.2767 | 0.2698 |
| 0.74 - 0.72 | 1661 | 1664 | 99.8 | 3.25 | 3.17 | 3.11 | 0.2912 | 0.3052 |
| 0.72 - 0.71 | 917 | 922 | 99.5 | 3.15 | 2.91 | 2.74 | 0.3246 | 0.3436 |
| 0.71 - 0.70 | 940 | 943 | 99.7 | 3.15 | 2.61 | 2.49 | 0.3546 | 0.3855 |
| 0.70 - 0.69 | 1063 | 1221 | 87.1 | 2.26 | 2.32 | 1.93 | 0.3802 | 0.5281 |
| 0.79 - 0.69 | 7993 | 8166 | 97.9 | 3.16 | 3.32 | 3.28 | 0.2809 | 0.2953 |
| Inf - 0.69 | 23900 | 24073 | 99.3 | 5.23 | 13.40 | 16.23 | 0.0595 | 0.0587 |

Complete .cif-data of the compound are available under the CCDC number CCDC-**2214321**.

A resolution cut off (SHEL 999 0.73) was applied to exclude reflections with poor signal to noise ratio at higher 2Θ angles. The structure contains several disordered areas in the main part as well as in the solute molecules. Two aromatic rings of the cationic Bi species show a disorder over two positions with a fixed occupancy of 65:35% each. The AFIX 66 instruction was applied to force six membered aromatic rings into a hexagonal shape. The ISOR instruction (ISOR 0.02 0.01) was used to treat atomic displacement parameters of one disordered phenyl ring of the cationic entity.

All minor parts have been described using isotropic atomic displacement parameters. Four of the eight terminal –$CF_3$ groups of the $BAr^F$ anion show rotational disorders over two or more positions. The occupancies of the F atoms where fixed at 25:25:50% or 20:20:60%. Additional disorders could be found in the solute molecules. The DSR tool plug in in Olex2 was used to describe the disorders of the 1,4-dioxane over three positions with an occupancy of 33.333% each. Additionally, one pentane molecule is located on and disordered over a crystallographic special position (inversion centre) with an occupancy fixed at 50:50%. Hydrogen atoms were placed in geometrically calculated positions and refined using an appropriate riding model. Hydrogen atoms at C7 and C8 were found by inspection of the residual electron density map during later refinement cycles. This finding is in accordance with short N–C (N1–C7 = 1.280 Å; N2–C8 = 1.284 Å) distances and confirms the presence of two N=C double bonds in the solid state.

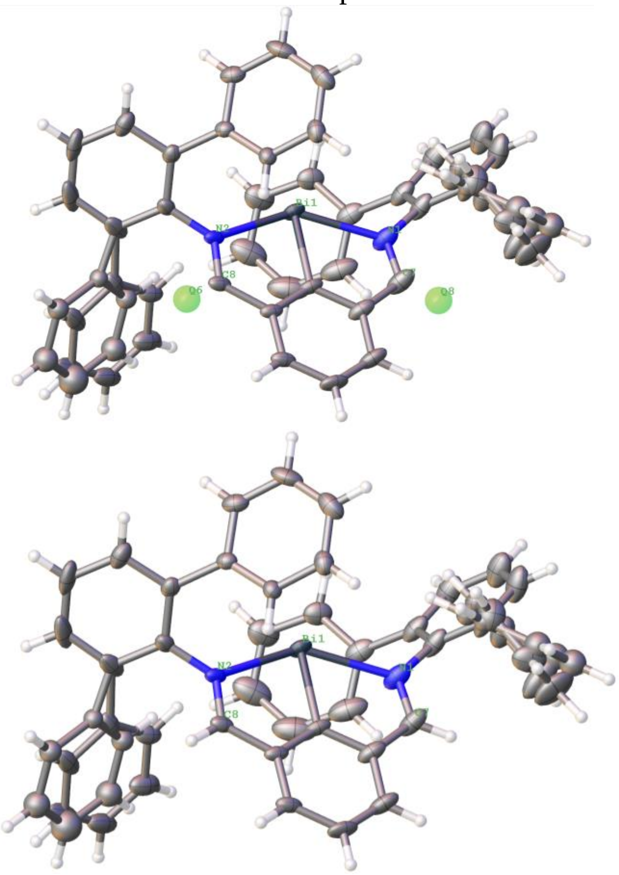

Figure S34. Solid-state structure of the cationic Bi(II) unit of **3**·THF·pentane solvate. Hydrogen atom positions attached to the C7 and C8 carbon atoms could be observed as residual electron density peaks (green) during later refinement.

Table S9**.** Crystal data and structure refinement of compound **3**·THF·pentane solvate

| | | |
|---|---|---|
| Identification code | 14528 | |
| Empirical formula | $C_{165}$ $H_{114}$ $B_2$ $Bi_2$ $F_{48}$ $N_4$ $O_4$ | |
| Color | orange | |
| Formula weight | 3568.18 g·mol$^{-1}$ | |
| Temperature | 100(2) K | |
| Wavelength | 0.71073 Å | |
| Crystal system | Triclinic | |
| Space group | *P*-1, (no. 2) | |
| Unit cell dimensions | a = 13.3187(6) Å | α= 102.770(2)°. |
| | b = 17.2111(7) Å | β= 100.007(2)°. |
| | c = 18.5792(9) Å | γ = 110.397(2)°. |
| Volume | 3743.7(3) Å$^3$ | |
| Z | 1 | |
| Density (calculated) | 1.583 Mg·m$^{-3}$ | |
| Absorption coefficient | 2.464 mm$^{-1}$ | |
| F(000) | 1772 e | |
| Crystal size | 0.314 x 0.142 x 0.02 mm$^3$ | |
| θ range for data collection | 1.169 to 29.130°. | |
| Index ranges | -18 ≤ h ≤ 18, -23≤ k ≤ 23, -25 ≤ l ≤ 25 | |
| Reflections collected | 114687 | |
| Independent reflections | 20167 [$R_{int}$ = 0.0570] | |
| Reflections with I>2σ(I) | 16174 | |
| Completeness to θ = 25.242° | 100.0 % | |
| Absorption correction | Gaussian | |
| Max. and min. transmission | 0.91621 and 0.48561 | |
| Refinement method | Full-matrix least-squares on $F^2$ | |
| Data / restraints / parameters | 20167 / 80 / 1141 | |
| Goodness-of-fit on $F^2$ | 1.024 | |
| Final R indices [I>2σ(I)] | $R_1$ = 0.0411 | $wR^2$ = 0.0842 |
| R indices (all data) | $R_1$ = 0.0611 | $wR^2$ = 0.0917 |
| Extinction coefficient | n/a | |
| Largest diff. peak and hole | 1.894 and -2.073 e·Å$^{-3}$ | |

Table S10**.** Bond lengths [Å] and angles [°] of compound **3**·THF·pentane solvate

| | | | |
|---|---|---|---|
| Bi(1)-N(1) | 2.423(3) | Bi(1)-N(2) | 2.505(3) |
| Bi(1)-C(1) | 2.177(3) | N(1)-C(7) | 1.280(5) |
| N(1)-C(9) | 1.430(5) | N(2)-C(8) | 1.284(4) |
| N(2)-C(27) | 1.423(4) | C(1)-C(2) | 1.390(5) |
| C(1)-C(6) | 1.387(5) | C(2)-C(3) | 1.406(5) |
| C(2)-C(7) | 1.449(6) | C(3)-H(3) | 0.9500 |
| C(3)-C(4) | 1.374(6) | C(4)-H(4) | 0.9500 |
| C(4)-C(5) | 1.390(5) | C(5)-H(5) | 0.9500 |
| C(5)-C(6) | 1.402(4) | C(6)-C(8) | 1.454(5) |
| C(7)-H(7) | 0.9500 | C(8)-H(8) | 0.9500 |
| C(9)-C(10) | 1.412(5) | C(9)-C(14) | 1.396(6) |
| C(10)-C(11) | 1.392(6) | C(10)-C(15) | 1.481(6) |
| C(11)-H(11) | 0.9500 | C(11)-C(12) | 1.371(6) |
| C(12)-H(12) | 0.9500 | C(12)-C(13) | 1.390(6) |
| C(13)-H(13) | 0.9500 | C(13)-C(14) | 1.385(6) |
| C(14)-C(21) | 1.496(5) | C(15)-C(16) | 1.395(6) |
| C(15)-C(20) | 1.391(5) | C(16)-H(16) | 0.9500 |
| C(16)-C(17) | 1.386(7) | C(17)-H(17) | 0.9500 |
| C(17)-C(18) | 1.387(7) | C(18)-H(18) | 0.9500 |
| C(18)-C(19) | 1.388(6) | C(19)-H(19) | 0.9500 |
| C(19)-C(20) | 1.396(6) | C(20)-H(20) | 0.9500 |
| C(21)-C(22B) | 1.3900 | C(21)-C(26B) | 1.3900 |
| C(21)-C(22A) | 1.423(9) | C(21)-C(26A) | 1.379(8) |
| C(22B)-H(22B) | 0.9500 | C(22B)-C(23B) | 1.3900 |
| C(23B)-H(23B) | 0.9500 | C(23B)-C(24B) | 1.3900 |
| C(24B)-H(24B) | 0.9500 | C(24B)-C(25B) | 1.3900 |
| C(25B)-H(25B) | 0.9500 | C(25B)-C(26B) | 1.3900 |
| C(26B)-H(26B) | 0.9500 | C(22A)-H(22A) | 0.9500 |
| C(22A)-C(23A) | 1.400(10) | C(23A)-H(23A) | 0.9500 |
| C(23A)-C(24A) | 1.344(11) | C(24A)-H(24A) | 0.9500 |
| C(24A)-C(25A) | 1.386(11) | C(25A)-H(25A) | 0.9500 |
| C(25A)-C(26A) | 1.390(9) | C(26A)-H(26A) | 0.9500 |
| C(27)-C(28) | 1.410(5) | C(27)-C(32) | 1.398(5) |
| C(28)-C(29) | 1.399(5) | C(28)-C(33) | 1.478(4) |

| | | | |
|---|---|---|---|
| C(29)-H(29) | 0.9500 | C(29)-C(30) | 1.378(6) |
| C(30)-H(30) | 0.9500 | C(30)-C(31) | 1.386(7) |
| C(31)-H(31) | 0.9500 | C(31)-C(32) | 1.394(6) |
| C(32)-C(39A) | 1.612(9) | C(32)-C(39B) | 1.351(6) |
| C(33)-C(34) | 1.399(4) | C(33)-C(38) | 1.407(4) |
| C(34)-H(34) | 0.9500 | C(34)-C(35) | 1.387(4) |
| C(35)-H(35) | 0.9500 | C(35)-C(36) | 1.378(5) |
| C(36)-H(36) | 0.9500 | C(36)-C(37) | 1.377(6) |
| C(37)-H(37) | 0.9500 | C(37)-C(38) | 1.384(5) |
| C(38)-H(38) | 0.9500 | C(39A)-C(40A) | 1.409(10) |
| C(39A)-C(44A) | 1.372(7) | C(39B)-C(45B) | 1.3900 |
| C(39B)-C(40B) | 1.3900 | C(45B)-H(45B) | 0.9500 |
| C(45B)-C(43B) | 1.3900 | C(43B)-H(43B) | 0.9500 |
| C(43B)-C(42B) | 1.3900 | C(42B)-H(42B) | 0.9500 |
| C(42B)-C(41B) | 1.3900 | C(41B)-H(41B) | 0.9500 |
| C(41B)-C(40B) | 1.3900 | C(40B)-H(40B) | 0.9500 |
| C(40A)-H(40A) | 0.9500 | C(40A)-C(41A) | 1.397(8) |
| C(41A)-H(41A) | 0.9500 | C(41A)-C(42A) | 1.376(10) |
| C(42A)-H(42A) | 0.9500 | C(42A)-C(43A) | 1.373(12) |
| C(43A)-H(43A) | 0.9500 | C(43A)-C(44A) | 1.426(11) |
| C(44A)-H(44A) | 0.9500 | F(1)-C(52) | 1.339(4) |
| F(2)-C(52) | 1.339(4) | F(3)-C(52) | 1.337(4) |
| F(4)-C(53) | 1.330(4) | F(5)-C(53) | 1.327(4) |
| F(6)-C(53) | 1.316(4) | F(7A)-C(60) | 1.348(6) |
| F(7B)-C(60) | 1.271(19) | F(7C)-C(60) | 1.34(2) |
| F(8A)-C(60) | 1.339(6) | F(8B)-C(60) | 1.36(2) |
| F(8C)-C(60) | 1.276(18) | F(9A)-C(60) | 1.331(7) |
| F(9B)-C(60) | 1.356(19) | F(9C)-C(60) | 1.35(2) |
| F(10)-C(61) | 1.347(4) | F(11)-C(61) | 1.334(5) |
| F(12)-C(61) | 1.340(4) | F(13)-C(68) | 1.333(5) |
| F(14)-C(68) | 1.326(4) | F(15)-C(68) | 1.348(4) |
| F(16A)-C(69) | 1.344(7) | F(16B)-C(69) | 1.376(13) |
| F(16C)-C(69) | 1.300(12) | F(17A)-C(69) | 1.325(7) |
| F(17B)-C(69) | 1.361(12) | F(17C)-C(69) | 1.415(14) |
| F(18A)-C(69) | 1.320(6) | F(18B)-C(69) | 1.242(11) |
| F(18C)-C(69) | 1.351(14) | F(19A)-C(76) | 1.413(7) |

| | | | |
|---|---|---|---|
| F(19B)-C(76) | 1.20(2) | F(19C)-C(76) | 1.331(16) |
| F(20A)-C(76) | 1.330(6) | F(20B)-C(76) | 1.367(17) |
| F(20C)-C(76) | 1.350(14) | F(21A)-C(76) | 1.333(7) |
| F(21B)-C(76) | 1.370(16) | F(21C)-C(76) | 1.330(14) |
| F(22A)-C(77) | 1.387(9) | F(22B)-C(77) | 1.234(19) |
| F(22C)-C(77) | 1.326(15) | F(23A)-C(77) | 1.442(10) |
| F(23B)-C(77) | 1.164(13) | F(23C)-C(77) | 1.31(2) |
| F(24A)-C(77) | 1.310(6) | F(24B)-C(77) | 1.460(11) |
| F(24C)-C(77) | 1.416(14) | C(46)-C(47) | 1.410(4) |
| C(46)-C(51) | 1.392(4) | C(46)-B(1) | 1.634(5) |
| C(47)-H(47) | 0.9500 | C(47)-C(48) | 1.382(4) |
| C(48)-C(49) | 1.390(5) | C(48)-C(52) | 1.495(4) |
| C(49)-H(49) | 0.9500 | C(49)-C(50) | 1.380(4) |
| C(50)-C(51) | 1.393(5) | C(50)-C(53) | 1.496(5) |
| C(51)-H(51) | 0.9500 | C(54)-C(55) | 1.399(4) |
| C(54)-C(59) | 1.396(4) | C(54)-B(1) | 1.638(4) |
| C(55)-H(55) | 0.9500 | C(55)-C(56) | 1.387(4) |
| C(56)-C(57) | 1.384(5) | C(56)-C(60) | 1.499(5) |
| C(57)-H(57) | 0.9500 | C(57)-C(58) | 1.379(5) |
| C(58)-C(59) | 1.386(4) | C(58)-C(61) | 1.486(5) |
| C(59)-H(59) | 0.9500 | C(62)-C(63) | 1.398(4) |
| C(62)-C(67) | 1.403(4) | C(62)-B(1) | 1.640(4) |
| C(63)-H(63) | 0.9500 | C(63)-C(64) | 1.398(4) |
| C(64)-C(65) | 1.384(5) | C(64)-C(68) | 1.482(5) |
| C(65)-H(65) | 0.9500 | C(65)-C(66) | 1.375(5) |
| C(66)-C(67) | 1.393(4) | C(66)-C(69) | 1.496(5) |
| C(67)-H(67) | 0.9500 | C(70)-C(71) | 1.392(5) |
| C(70)-C(75) | 1.403(4) | C(70)-B(1) | 1.644(4) |
| C(71)-H(71) | 0.9500 | C(71)-C(72) | 1.388(4) |
| C(72)-C(73) | 1.384(5) | C(72)-C(76) | 1.488(5) |
| C(73)-H(73) | 0.9500 | C(73)-C(74) | 1.376(5) |
| C(74)-C(75) | 1.394(4) | C(74)-C(77) | 1.495(5) |
| C(75)-H(75) | 0.9500 | O(1A)-C(84A) | 1.401(12) |
| O(1A)-C(85A) | 1.420(12) | O(2A)-C(83A) | 1.30(2) |
| O(2A)-C(86A) | 1.53(4) | C(83A)-H(83A) | 0.9900 |
| C(83A)-H(83B) | 0.9900 | C(83A)-C(84A) | 1.516(13) |

| | | | |
|---|---|---|---|
| C(84A)-H(84A) | 0.9900 | C(84A)-H(84B) | 0.9900 |
| C(85A)-H(85A) | 0.9900 | C(85A)-H(85B) | 0.9900 |
| C(85A)-C(86A) | 1.524(15) | C(86A)-H(86A) | 0.9900 |
| C(86A)-H(86B) | 0.9900 | O(1B)-C(83B) | 1.417(11) |
| O(1B)-C(86B) | 1.454(12) | O(2B)-C(84B) | 1.421(11) |
| O(2B)-C(85B) | 1.406(12) | C(83B)-H(83C) | 0.9900 |
| C(83B)-H(83D) | 0.9900 | C(83B)-C(84B) | 1.475(12) |
| C(84B)-H(84C) | 0.9900 | C(84B)-H(84D) | 0.9900 |
| C(85B)-H(85C) | 0.9900 | C(85B)-H(85D) | 0.9900 |
| C(85B)-C(86B) | 1.462(13) | C(86B)-H(86C) | 0.9900 |
| C(86B)-H(86D) | 0.9900 | O(1C)-C(83C) | 1.64(3) |
| O(1C)-C(86C) | 1.41(3) | O(2C)-C(84C) | 1.58(4) |
| O(2C)-C(85C) | 1.34(3) | C(83C)-H(83E) | 0.9900 |
| C(83C)-H(83F) | 0.9900 | C(83C)-C(84C) | 1.42(4) |
| C(84C)-H(84E) | 0.9900 | C(84C)-H(84F) | 0.9900 |
| C(85C)-H(85E) | 0.9900 | C(85C)-H(85F) | 0.9900 |
| C(85C)-C(86C) | 1.31(3) | C(86C)-H(86E) | 0.9900 |
| C(86C)-H(86F) | 0.9900 | C(78)-H(78A) | 0.9800 |
| C(78)-H(78B) | 0.9800 | C(78)-H(78C) | 0.9800 |
| C(78)-C(79) | 1.115(13) | C(79)-H(79A) | 0.9900 |
| C(79)-H(79B) | 0.9900 | C(79)-C(80) | 1.540(14) |
| C(80)-H(80A) | 0.9900 | C(80)-H(80B) | 0.9900 |
| C(80)-C(81) | 1.433(11) | C(81)-H(81A) | 0.9900 |
| C(81)-H(81B) | 0.9900 | C(81)-C(82) | 1.559(9) |
| C(82)-H(82A) | 0.9800 | C(82)-H(82B) | 0.9800 |
| C(82)-H(82C) | 0.9800 | | |

| | | | |
|---|---|---|---|
| N(1)-Bi(1)-N(2) | 142.36(9) | C(1)-Bi(1)-N(1) | 72.02(12) |
| C(1)-Bi(1)-N(2) | 70.43(11) | C(7)-N(1)-Bi(1) | 113.1(3) |
| C(7)-N(1)-C(9) | 123.5(3) | C(9)-N(1)-Bi(1) | 123.1(2) |
| C(8)-N(2)-Bi(1) | 112.5(2) | C(8)-N(2)-C(27) | 121.3(3) |
| C(27)-N(2)-Bi(1) | 126.14(19) | C(2)-C(1)-Bi(1) | 118.4(3) |
| C(6)-C(1)-Bi(1) | 120.6(2) | C(6)-C(1)-C(2) | 121.0(3) |
| C(1)-C(2)-C(3) | 119.1(4) | C(1)-C(2)-C(7) | 117.4(3) |
| C(3)-C(2)-C(7) | 123.5(3) | C(2)-C(3)-H(3) | 120.0 |
| C(4)-C(3)-C(2) | 120.0(3) | C(4)-C(3)-H(3) | 120.0 |

| | | | |
|---|---|---|---|
| C(3)-C(4)-H(4) | 119.6 | C(3)-C(4)-C(5) | 120.8(3) |
| C(5)-C(4)-H(4) | 119.6 | C(4)-C(5)-H(5) | 120.1 |
| C(4)-C(5)-C(6) | 119.7(3) | C(6)-C(5)-H(5) | 120.1 |
| C(1)-C(6)-C(5) | 119.3(3) | C(1)-C(6)-C(8) | 117.6(3) |
| C(5)-C(6)-C(8) | 123.1(3) | N(1)-C(7)-C(2) | 119.0(3) |
| N(1)-C(7)-H(7) | 120.5 | C(2)-C(7)-H(7) | 120.5 |
| N(2)-C(8)-C(6) | 118.6(3) | N(2)-C(8)-H(8) | 120.7 |
| C(6)-C(8)-H(8) | 120.7 | C(10)-C(9)-N(1) | 116.5(4) |
| C(14)-C(9)-N(1) | 121.0(3) | C(14)-C(9)-C(10) | 122.1(4) |
| C(9)-C(10)-C(15) | 120.5(3) | C(11)-C(10)-C(9) | 117.5(4) |
| C(11)-C(10)-C(15) | 121.9(3) | C(10)-C(11)-H(11) | 119.5 |
| C(12)-C(11)-C(10) | 121.0(4) | C(12)-C(11)-H(11) | 119.5 |
| C(11)-C(12)-H(12) | 119.7 | C(11)-C(12)-C(13) | 120.5(4) |
| C(13)-C(12)-H(12) | 119.7 | C(12)-C(13)-H(13) | 119.6 |
| C(14)-C(13)-C(12) | 120.8(4) | C(14)-C(13)-H(13) | 119.6 |
| C(9)-C(14)-C(21) | 121.3(4) | C(13)-C(14)-C(9) | 118.0(4) |
| C(13)-C(14)-C(21) | 120.6(4) | C(16)-C(15)-C(10) | 121.5(4) |
| C(20)-C(15)-C(10) | 120.1(3) | C(20)-C(15)-C(16) | 118.3(4) |
| C(15)-C(16)-H(16) | 119.6 | C(17)-C(16)-C(15) | 120.8(4) |
| C(17)-C(16)-H(16) | 119.6 | C(16)-C(17)-H(17) | 119.9 |
| C(16)-C(17)-C(18) | 120.3(4) | C(18)-C(17)-H(17) | 119.9 |
| C(17)-C(18)-H(18) | 120.1 | C(17)-C(18)-C(19) | 119.8(5) |
| C(19)-C(18)-H(18) | 120.1 | C(18)-C(19)-H(19) | 120.2 |
| C(18)-C(19)-C(20) | 119.6(4) | C(20)-C(19)-H(19) | 120.2 |
| C(15)-C(20)-C(19) | 121.2(4) | C(15)-C(20)-H(20) | 119.4 |
| C(19)-C(20)-H(20) | 119.4 | C(22B)-C(21)-C(14) | 120.4(4) |
| C(22B)-C(21)-C(26B) | 120.0 | C(26B)-C(21)-C(14) | 119.4(4) |
| C(22A)-C(21)-C(14) | 120.4(5) | C(26A)-C(21)-C(14) | 120.5(4) |
| C(26A)-C(21)-C(22A) | 118.6(5) | C(21)-C(22B)-H(22B) | 120.0 |
| C(21)-C(22B)-C(23B) | 120.0 | C(23B)-C(22B)-H(22B) | 120.0 |
| C(22B)-C(23B)-H(23B) | 120.0 | C(24B)-C(23B)-C(22B) | 120.0 |
| C(24B)-C(23B)-H(23B) | 120.0 | C(23B)-C(24B)-H(24B) | 120.0 |
| C(23B)-C(24B)-C(25B) | 120.0 | C(25B)-C(24B)-H(24B) | 120.0 |
| C(24B)-C(25B)-H(25B) | 120.0 | C(26B)-C(25B)-C(24B) | 120.0 |
| C(26B)-C(25B)-H(25B) | 120.0 | C(21)-C(26B)-H(26B) | 120.0 |
| C(25B)-C(26B)-C(21) | 120.0 | C(25B)-C(26B)-H(26B) | 120.0 |

| | | | |
|---|---|---|---|
| C(21)-C(22A)-H(22A) | 120.7 | C(23A)-C(22A)-C(21) | 118.7(7) |
| C(23A)-C(22A)-H(22A) | 120.7 | C(22A)-C(23A)-H(23A) | 119.3 |
| C(24A)-C(23A)-C(22A) | 121.4(7) | C(24A)-C(23A)-H(23A) | 119.3 |
| C(23A)-C(24A)-H(24A) | 119.8 | C(23A)-C(24A)-C(25A) | 120.3(6) |
| C(25A)-C(24A)-H(24A) | 119.8 | C(24A)-C(25A)-H(25A) | 120.1 |
| C(24A)-C(25A)-C(26A) | 119.8(7) | C(26A)-C(25A)-H(25A) | 120.1 |
| C(21)-C(26A)-C(25A) | 120.8(6) | C(21)-C(26A)-H(26A) | 119.6 |
| C(25A)-C(26A)-H(26A) | 119.6 | C(28)-C(27)-N(2) | 117.3(3) |
| C(32)-C(27)-N(2) | 121.0(3) | C(32)-C(27)-C(28) | 121.7(3) |
| C(27)-C(28)-C(33) | 122.7(3) | C(29)-C(28)-C(27) | 118.1(3) |
| C(29)-C(28)-C(33) | 119.2(3) | C(28)-C(29)-H(29) | 119.6 |
| C(30)-C(29)-C(28) | 120.8(4) | C(30)-C(29)-H(29) | 119.6 |
| C(29)-C(30)-H(30) | 120.0 | C(29)-C(30)-C(31) | 119.9(4) |
| C(31)-C(30)-H(30) | 120.0 | C(30)-C(31)-H(31) | 119.2 |
| C(30)-C(31)-C(32) | 121.7(3) | C(32)-C(31)-H(31) | 119.2 |
| C(27)-C(32)-C(39A) | 118.0(4) | C(31)-C(32)-C(27) | 117.6(4) |
| C(31)-C(32)-C(39A) | 123.8(4) | C(39B)-C(32)-C(27) | 132.7(5) |
| C(39B)-C(32)-C(31) | 107.7(4) | C(34)-C(33)-C(28) | 124.1(3) |
| C(34)-C(33)-C(38) | 117.1(3) | C(38)-C(33)-C(28) | 118.8(3) |
| C(33)-C(34)-H(34) | 119.2 | C(35)-C(34)-C(33) | 121.7(3) |
| C(35)-C(34)-H(34) | 119.2 | C(34)-C(35)-H(35) | 120.0 |
| C(36)-C(35)-C(34) | 120.0(3) | C(36)-C(35)-H(35) | 120.0 |
| C(35)-C(36)-H(36) | 120.2 | C(37)-C(36)-C(35) | 119.5(3) |
| C(37)-C(36)-H(36) | 120.2 | C(36)-C(37)-H(37) | 119.5 |
| C(36)-C(37)-C(38) | 121.0(3) | C(38)-C(37)-H(37) | 119.5 |
| C(33)-C(38)-H(38) | 119.6 | C(37)-C(38)-C(33) | 120.7(3) |
| C(37)-C(38)-H(38) | 119.6 | C(40A)-C(39A)-C(32) | 123.4(4) |
| C(44A)-C(39A)-C(32) | 118.0(7) | C(44A)-C(39A)-C(40A) | 118.5(6) |
| C(32)-C(39B)-C(45B) | 130.3(6) | C(32)-C(39B)-C(40B) | 109.6(6) |
| C(45B)-C(39B)-C(40B) | 120.0 | C(39B)-C(45B)-H(45B) | 120.0 |
| C(43B)-C(45B)-C(39B) | 120.0 | C(43B)-C(45B)-H(45B) | 120.0 |
| C(45B)-C(43B)-H(43B) | 120.0 | C(45B)-C(43B)-C(42B) | 120.0 |
| C(42B)-C(43B)-H(43B) | 120.0 | C(43B)-C(42B)-H(42B) | 120.0 |
| C(41B)-C(42B)-C(43B) | 120.0 | C(41B)-C(42B)-H(42B) | 120.0 |
| C(42B)-C(41B)-H(41B) | 120.0 | C(42B)-C(41B)-C(40B) | 120.0 |
| C(40B)-C(41B)-H(41B) | 120.0 | C(39B)-C(40B)-H(40B) | 120.0 |

| | | | |
|---|---|---|---|
| C(41B)-C(40B)-C(39B) | 120.0 | C(41B)-C(40B)-H(40B) | 120.0 |
| C(39A)-C(40A)-H(40A) | 119.2 | C(41A)-C(40A)-C(39A) | 121.6(6) |
| C(41A)-C(40A)-H(40A) | 119.2 | C(40A)-C(41A)-H(41A) | 120.6 |
| C(42A)-C(41A)-C(40A) | 118.8(8) | C(42A)-C(41A)-H(41A) | 120.6 |
| C(41A)-C(42A)-H(42A) | 119.5 | C(43A)-C(42A)-C(41A) | 120.9(8) |
| C(43A)-C(42A)-H(42A) | 119.5 | C(42A)-C(43A)-H(43A) | 119.9 |
| C(42A)-C(43A)-C(44A) | 120.2(8) | C(44A)-C(43A)-H(43A) | 119.9 |
| C(39A)-C(44A)-C(43A) | 119.9(8) | C(39A)-C(44A)-H(44A) | 120.1 |
| C(43A)-C(44A)-H(44A) | 120.1 | C(47)-C(46)-B(1) | 120.0(3) |
| C(51)-C(46)-C(47) | 115.5(3) | C(51)-C(46)-B(1) | 124.1(3) |
| C(46)-C(47)-H(47) | 118.9 | C(48)-C(47)-C(46) | 122.1(3) |
| C(48)-C(47)-H(47) | 118.9 | C(47)-C(48)-C(49) | 121.0(3) |
| C(47)-C(48)-C(52) | 121.2(3) | C(49)-C(48)-C(52) | 117.8(3) |
| C(48)-C(49)-H(49) | 121.0 | C(50)-C(49)-C(48) | 117.9(3) |
| C(50)-C(49)-H(49) | 121.0 | C(49)-C(50)-C(51) | 120.8(3) |
| C(49)-C(50)-C(53) | 119.9(3) | C(51)-C(50)-C(53) | 119.3(3) |
| C(46)-C(51)-C(50) | 122.5(3) | C(46)-C(51)-H(51) | 118.8 |
| C(50)-C(51)-H(51) | 118.8 | F(1)-C(52)-F(2) | 106.3(3) |
| F(1)-C(52)-C(48) | 112.0(3) | F(2)-C(52)-C(48) | 113.6(3) |
| F(3)-C(52)-F(1) | 105.3(3) | F(3)-C(52)-F(2) | 106.5(3) |
| F(3)-C(52)-C(48) | 112.5(3) | F(4)-C(53)-C(50) | 112.1(3) |
| F(5)-C(53)-F(4) | 104.8(3) | F(5)-C(53)-C(50) | 112.7(3) |
| F(6)-C(53)-F(4) | 106.6(3) | F(6)-C(53)-F(5) | 106.9(3) |
| F(6)-C(53)-C(50) | 113.1(3) | C(55)-C(54)-B(1) | 123.4(3) |
| C(59)-C(54)-C(55) | 115.6(3) | C(59)-C(54)-B(1) | 120.6(3) |
| C(54)-C(55)-H(55) | 118.7 | C(56)-C(55)-C(54) | 122.5(3) |
| C(56)-C(55)-H(55) | 118.7 | C(55)-C(56)-C(60) | 119.9(3) |
| C(57)-C(56)-C(55) | 120.3(3) | C(57)-C(56)-C(60) | 119.8(3) |
| C(56)-C(57)-H(57) | 120.8 | C(58)-C(57)-C(56) | 118.5(3) |
| C(58)-C(57)-H(57) | 120.8 | C(57)-C(58)-C(59) | 120.8(3) |
| C(57)-C(58)-C(61) | 119.4(3) | C(59)-C(58)-C(61) | 119.8(3) |
| C(54)-C(59)-H(59) | 118.9 | C(58)-C(59)-C(54) | 122.3(3) |
| C(58)-C(59)-H(59) | 118.9 | F(7A)-C(60)-C(56) | 112.6(4) |
| F(7B)-C(60)-F(8B) | 108.3(12) | F(7B)-C(60)-F(9B) | 105.8(11) |
| F(7B)-C(60)-C(56) | 114.2(9) | F(7C)-C(60)-F(9C) | 105.6(12) |
| F(7C)-C(60)-C(56) | 111.8(9) | F(8A)-C(60)-F(7A) | 107.3(6) |

| | | | |
|---|---|---|---|
| F(8A)-C(60)-C(56) | 112.7(3) | F(8B)-C(60)-C(56) | 109.5(8) |
| F(8C)-C(60)-F(7C) | 109.8(12) | F(8C)-C(60)-F(9C) | 102.2(12) |
| F(8C)-C(60)-C(56) | 115.0(8) | F(9A)-C(60)-F(7A) | 106.0(5) |
| F(9A)-C(60)-F(8A) | 106.5(6) | F(9A)-C(60)-C(56) | 111.4(4) |
| F(9B)-C(60)-F(8B) | 105.9(11) | F(9B)-C(60)-C(56) | 112.7(8) |
| F(9C)-C(60)-C(56) | 111.5(10) | F(10)-C(61)-C(58) | 112.4(3) |
| F(11)-C(61)-F(10) | 105.9(3) | F(11)-C(61)-F(12) | 106.9(3) |
| F(11)-C(61)-C(58) | 112.7(3) | F(12)-C(61)-F(10) | 105.8(3) |
| F(12)-C(61)-C(58) | 112.6(3) | C(63)-C(62)-C(67) | 115.4(3) |
| C(63)-C(62)-B(1) | 122.9(3) | C(67)-C(62)-B(1) | 121.3(3) |
| C(62)-C(63)-H(63) | 118.8 | C(64)-C(63)-C(62) | 122.3(3) |
| C(64)-C(63)-H(63) | 118.8 | C(63)-C(64)-C(68) | 121.5(3) |
| C(65)-C(64)-C(63) | 120.4(3) | C(65)-C(64)-C(68) | 118.2(3) |
| C(64)-C(65)-H(65) | 120.6 | C(66)-C(65)-C(64) | 118.8(3) |
| C(66)-C(65)-H(65) | 120.6 | C(65)-C(66)-C(67) | 120.6(3) |
| C(65)-C(66)-C(69) | 119.5(3) | C(67)-C(66)-C(69) | 119.9(3) |
| C(62)-C(67)-H(67) | 118.8 | C(66)-C(67)-C(62) | 122.4(3) |
| C(66)-C(67)-H(67) | 118.8 | F(13)-C(68)-F(15) | 105.2(3) |
| F(13)-C(68)-C(64) | 113.5(3) | F(14)-C(68)-F(13) | 107.3(3) |
| F(14)-C(68)-F(15) | 105.3(3) | F(14)-C(68)-C(64) | 112.9(3) |
| F(15)-C(68)-C(64) | 111.9(3) | F(16A)-C(69)-C(66) | 112.3(4) |
| F(16B)-C(69)-C(66) | 110.1(6) | F(16C)-C(69)-F(17C) | 112.6(9) |
| F(16C)-C(69)-F(18C) | 103.4(10) | F(16C)-C(69)-C(66) | 114.5(6) |
| F(17A)-C(69)-F(16A) | 102.7(6) | F(17A)-C(69)-C(66) | 113.5(4) |
| F(17B)-C(69)-F(16B) | 109.7(9) | F(17B)-C(69)-C(66) | 110.1(6) |
| F(17C)-C(69)-C(66) | 105.7(6) | F(18A)-C(69)-F(16A) | 105.4(5) |
| F(18A)-C(69)-F(17A) | 105.7(6) | F(18A)-C(69)-C(66) | 116.0(3) |
| F(18B)-C(69)-F(16B) | 106.0(9) | F(18B)-C(69)-F(17B) | 105.5(9) |
| F(18B)-C(69)-C(66) | 115.3(6) | F(18C)-C(69)-F(17C) | 108.7(10) |
| F(18C)-C(69)-C(66) | 112.0(7) | C(71)-C(70)-C(75) | 116.2(3) |
| C(71)-C(70)-B(1) | 119.8(3) | C(75)-C(70)-B(1) | 123.7(3) |
| C(70)-C(71)-H(71) | 118.7 | C(72)-C(71)-C(70) | 122.6(3) |
| C(72)-C(71)-H(71) | 118.7 | C(71)-C(72)-C(76) | 119.3(3) |
| C(73)-C(72)-C(71) | 120.2(3) | C(73)-C(72)-C(76) | 120.6(3) |
| C(72)-C(73)-H(73) | 120.7 | C(74)-C(73)-C(72) | 118.6(3) |
| C(74)-C(73)-H(73) | 120.7 | C(73)-C(74)-C(75) | 121.1(3) |

| | | | |
|---|---|---|---|
| C(73)-C(74)-C(77) | 119.2(3) | C(75)-C(74)-C(77) | 119.6(3) |
| C(70)-C(75)-H(75) | 119.4 | C(74)-C(75)-C(70) | 121.3(3) |
| C(74)-C(75)-H(75) | 119.4 | F(19A)-C(76)-C(72) | 110.8(3) |
| F(19B)-C(76)-F(20B) | 115.3(11) | F(19B)-C(76)-F(21B) | 102.2(11) |
| F(19B)-C(76)-C(72) | 114.3(10) | F(19C)-C(76)-F(20C) | 103.8(9) |
| F(19C)-C(76)-C(72) | 115.0(7) | F(20A)-C(76)-F(19A) | 104.0(5) |
| F(20A)-C(76)-F(21A) | 107.9(5) | F(20A)-C(76)-C(72) | 115.2(4) |
| F(20B)-C(76)-F(21B) | 101.7(9) | F(20B)-C(76)-C(72) | 110.1(8) |
| F(20C)-C(76)-C(72) | 109.8(7) | F(21A)-C(76)-F(19A) | 104.7(5) |
| F(21A)-C(76)-C(72) | 113.3(4) | F(21B)-C(76)-C(72) | 112.3(7) |
| F(21C)-C(76)-F(19C) | 110.7(10) | F(21C)-C(76)-F(20C) | 105.0(8) |
| F(21C)-C(76)-C(72) | 111.8(7) | F(22A)-C(77)-F(23A) | 103.6(8) |
| F(22A)-C(77)-C(74) | 108.3(5) | F(22B)-C(77)-F(24B) | 86.8(13) |
| F(22B)-C(77)-C(74) | 122.1(8) | F(22C)-C(77)-F(24C) | 108.5(8) |
| F(22C)-C(77)-C(74) | 114.8(9) | F(23A)-C(77)-C(74) | 111.2(4) |
| F(23B)-C(77)-F(22B) | 108.2(11) | F(23B)-C(77)-F(24B) | 110.0(8) |
| F(23B)-C(77)-C(74) | 117.9(6) | F(23C)-C(77)-F(22C) | 106.4(10) |
| F(23C)-C(77)-F(24C) | 99.5(10) | F(23C)-C(77)-C(74) | 114.4(9) |
| F(24A)-C(77)-F(22A) | 114.0(6) | F(24A)-C(77)-F(23A) | 105.5(6) |
| F(24A)-C(77)-C(74) | 113.7(4) | F(24B)-C(77)-C(74) | 107.0(5) |
| F(24C)-C(77)-C(74) | 112.1(6) | C(46)-B(1)-C(54) | 103.0(2) |
| C(46)-B(1)-C(62) | 112.9(3) | C(46)-B(1)-C(70) | 113.4(2) |
| C(54)-B(1)-C(62) | 113.0(2) | C(54)-B(1)-C(70) | 111.7(3) |
| C(62)-B(1)-C(70) | 103.2(2) | C(84A)-O(1A)-C(85A) | 110.1(11) |
| C(83A)-O(2A)-C(86A) | 104.5(17) | O(2A)-C(83A)-H(83A) | 110.7 |
| O(2A)-C(83A)-H(83B) | 110.7 | O(2A)-C(83A)-C(84A) | 105.2(13) |
| H(83A)-C(83A)-H(83B) | 108.8 | C(84A)-C(83A)-H(83A) | 110.7 |
| C(84A)-C(83A)-H(83B) | 110.7 | O(1A)-C(84A)-C(83A) | 109.0(10) |
| O(1A)-C(84A)-H(84A) | 109.9 | O(1A)-C(84A)-H(84B) | 109.9 |
| C(83A)-C(84A)-H(84A) | 109.9 | C(83A)-C(84A)-H(84B) | 109.9 |
| H(84A)-C(84A)-H(84B) | 108.3 | O(1A)-C(85A)-H(85A) | 109.6 |
| O(1A)-C(85A)-H(85B) | 109.6 | O(1A)-C(85A)-C(86A) | 110.5(14) |
| H(85A)-C(85A)-H(85B) | 108.1 | C(86A)-C(85A)-H(85A) | 109.6 |
| C(86A)-C(85A)-H(85B) | 109.6 | O(2A)-C(86A)-H(86A) | 110.3 |
| O(2A)-C(86A)-H(86B) | 110.3 | C(85A)-C(86A)-O(2A) | 107.1(18) |
| C(85A)-C(86A)-H(86A) | 110.3 | C(85A)-C(86A)-H(86B) | 110.3 |

| | | | |
|---|---|---|---|
| H(86A)-C(86A)-H(86B) | 108.5 | C(83B)-O(1B)-C(86B) | 109.8(11) |
| C(85B)-O(2B)-C(84B) | 109.0(12) | O(1B)-C(83B)-H(83C) | 109.8 |
| O(1B)-C(83B)-H(83D) | 109.8 | O(1B)-C(83B)-C(84B) | 109.3(9) |
| H(83C)-C(83B)-H(83D) | 108.3 | C(84B)-C(83B)-H(83C) | 109.8 |
| C(84B)-C(83B)-H(83D) | 109.8 | O(2B)-C(84B)-C(83B) | 111.6(10) |
| O(2B)-C(84B)-H(84C) | 109.3 | O(2B)-C(84B)-H(84D) | 109.3 |
| C(83B)-C(84B)-H(84C) | 109.3 | C(83B)-C(84B)-H(84D) | 109.3 |
| H(84C)-C(84B)-H(84D) | 108.0 | O(2B)-C(85B)-H(85C) | 109.6 |
| O(2B)-C(85B)-H(85D) | 109.6 | O(2B)-C(85B)-C(86B) | 110.1(11) |
| H(85C)-C(85B)-H(85D) | 108.2 | C(86B)-C(85B)-H(85C) | 109.6 |
| C(86B)-C(85B)-H(85D) | 109.6 | O(1B)-C(86B)-C(85B) | 111.6(11) |
| O(1B)-C(86B)-H(86C) | 109.3 | O(1B)-C(86B)-H(86D) | 109.3 |
| C(85B)-C(86B)-H(86C) | 109.3 | C(85B)-C(86B)-H(86D) | 109.3 |
| H(86C)-C(86B)-H(86D) | 108.0 | C(86C)-O(1C)-C(83C) | 115.1(16) |
| C(85C)-O(2C)-C(84C) | 112(2) | O(1C)-C(83C)-H(83E) | 111.4 |
| O(1C)-C(83C)-H(83F) | 111.4 | H(83E)-C(83C)-H(83F) | 109.2 |
| C(84C)-C(83C)-O(1C) | 102(2) | C(84C)-C(83C)-H(83E) | 111.4 |
| C(84C)-C(83C)-H(83F) | 111.4 | O(2C)-C(84C)-H(84E) | 109.5 |
| O(2C)-C(84C)-H(84F) | 109.5 | C(83C)-C(84C)-O(2C) | 111(3) |
| C(83C)-C(84C)-H(84E) | 109.5 | C(83C)-C(84C)-H(84F) | 109.5 |
| H(84E)-C(84C)-H(84F) | 108.1 | O(2C)-C(85C)-H(85E) | 109.1 |
| O(2C)-C(85C)-H(85F) | 109.1 | H(85E)-C(85C)-H(85F) | 107.9 |
| C(86C)-C(85C)-O(2C) | 112(2) | C(86C)-C(85C)-H(85E) | 109.1 |
| C(86C)-C(85C)-H(85F) | 109.1 | O(1C)-C(86C)-H(86E) | 106.8 |
| O(1C)-C(86C)-H(86F) | 106.8 | C(85C)-C(86C)-O(1C) | 122(2) |
| C(85C)-C(86C)-H(86E) | 106.8 | C(85C)-C(86C)-H(86F) | 106.8 |
| H(86E)-C(86C)-H(86F) | 106.6 | H(78A)-C(78)-H(78B) | 109.5 |
| H(78A)-C(78)-H(78C) | 109.5 | H(78B)-C(78)-H(78C) | 109.5 |
| C(79)-C(78)-H(78A) | 109.5 | C(79)-C(78)-H(78B) | 109.5 |
| C(79)-C(78)-H(78C) | 109.5 | C(78)-C(79)-H(79A) | 107.3 |
| C(78)-C(79)-H(79B) | 107.3 | C(78)-C(79)-C(80) | 120.2(12) |
| H(79A)-C(79)-H(79B) | 106.9 | C(80)-C(79)-H(79A) | 107.3 |
| C(80)-C(79)-H(79B) | 107.3 | C(79)-C(80)-H(80A) | 110.6 |
| C(79)-C(80)-H(80B) | 110.6 | H(80A)-C(80)-H(80B) | 108.8 |
| C(81)-C(80)-C(79) | 105.5(8) | C(81)-C(80)-H(80A) | 110.6 |
| C(81)-C(80)-H(80B) | 110.6 | C(80)-C(81)-H(81A) | 107.9 |

| | | | |
|---|---|---|---|
| C(80)-C(81)-H(81B) | 107.9 | C(80)-C(81)-C(82) | 117.5(8) |
| H(81A)-C(81)-H(81B) | 107.2 | C(82)-C(81)-H(81A) | 107.9 |
| C(82)-C(81)-H(81B) | 107.9 | C(81)-C(82)-H(82A) | 109.5 |
| C(81)-C(82)-H(82B) | 109.5 | C(81)-C(82)-H(82C) | 109.5 |
| H(82A)-C(82)-H(82B) | 109.5 | H(82A)-C(82)-H(82C) | 109.5 |
| H(82B)-C(82)-H(82C) | 109.5 | | |

## 11.5 Single crystal structure analysis of compound **4**·1,2-difluorobenzene solvate

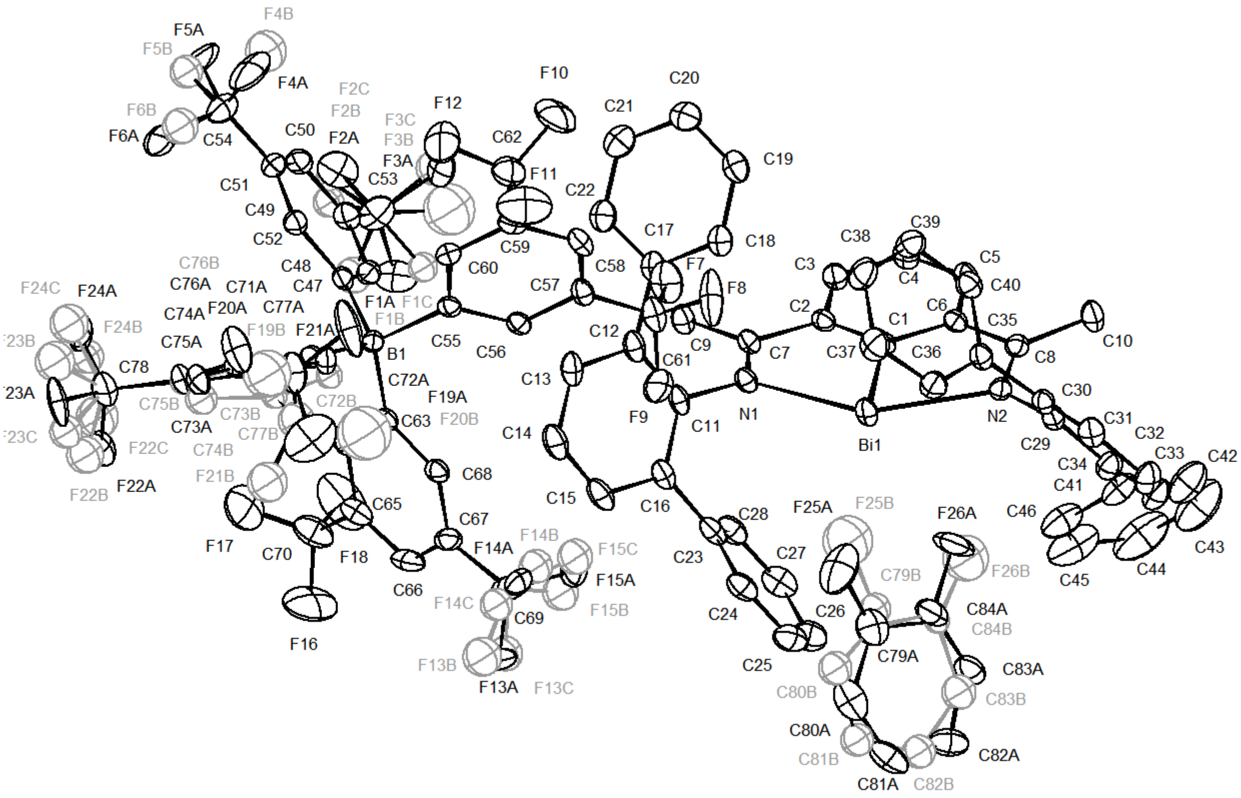


Figure S35. The molecular structure of compound **4**·1,2-difluorobenzene solvate. H atoms have been removed for clarity. Main structure shown in black and disordered parts shown in grey.

**X-ray Crystal Structure Analysis of compound 4·1,2-difluorobenzene solvate**:
$C_{84}H_{51}BBiF_{26}N_2$, $M_r$ = 1802.06 g mol$^{-1}$, orange prism, crystal size 0.278 x 0.074 x 0.068 mm$^3$, triclinic, space group *P*-1 [2], *a* = 13.8650(7) Å, b = 16.2579(8) Å, *c* = 17.5527(9) Å, $\alpha$= 102.879(2)°, $\beta$= 92.613(2)°, $\gamma$ = 98.564(2), *V* = 3801.4(3) Å$^3$, *T* = 100(2) K, *Z* = 2, $D_{calc}$ = 1.574 g·cm$^3$, $\lambda$ = 0.71073 Å, $\mu(Mo\text{-}K_\alpha)$ = 2.430 mm$^{-1}$, Gaussian absorption correction ($T_{min}$ = 0.76934, $T_{max}$ = 0.90078), Bruker-AXS Kappa Mach3 with APEX-II detector and IµS microfocus Mo-anode X-ray source, 1.194 < $\theta$ < 31.506°, 131508 measured reflections, 25294 independent reflections, 20187 reflections with $I > 2\sigma(I)$, $R_{int}$ = 0.0493. The structure was solved by *SHELXT* and refined by full-matrix least-squares (*SHELXL*) against $F^2$ to $R_1$ = 0.0347 [$I > 2\sigma(I)$], $wR_2$ = 0.0754 [all data], 1145 parameters and 42 restraints.

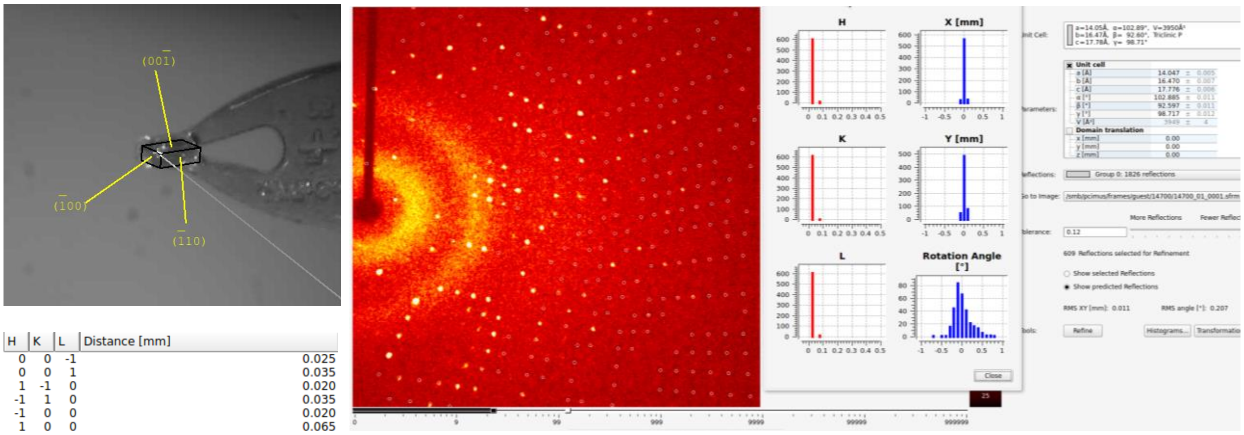


Figure S36. Crystal faces and unit cell determination/refinement of compound **4**·1,2-difluorobenzene solvate**.**

INTENSITY STATISTICS FOR DATASET

| Resolution | #Data | #Theory | %Complete | Redundancy | Mean I | Mean I/s | Rmerge | Rsigma |
|---|---|---|---|---|---|---|---|---|
| Inf - 2.52 | 499 | 499 | 100.0 | 9.35 | 106.68 | 82.92 | 0.0225 | 0.0099 |
| 2.52 - 1.68 | 1175 | 1175 | 100.0 | 9.53 | 59.77 | 67.76 | 0.0237 | 0.0115 |
| 1.68 - 1.33 | 1711 | 1711 | 100.0 | 9.39 | 36.89 | 54.65 | 0.0309 | 0.0144 |
| 1.33 - 1.16 | 1689 | 1689 | 100.0 | 9.11 | 26.83 | 43.21 | 0.0414 | 0.0183 |
| 1.16 - 1.06 | 1604 | 1604 | 100.0 | 7.88 | 21.86 | 33.57 | 0.0504 | 0.0244 |
| 1.06 - 0.98 | 1773 | 1773 | 100.0 | 5.82 | 17.88 | 23.58 | 0.0633 | 0.0360 |
| 0.98 - 0.92 | 1763 | 1763 | 100.0 | 4.79 | 13.77 | 17.18 | 0.0750 | 0.0508 |
| 0.92 - 0.88 | 1447 | 1447 | 100.0 | 4.19 | 11.57 | 13.69 | 0.0875 | 0.0646 |
| 0.88 - 0.84 | 1773 | 1773 | 100.0 | 3.92 | 10.01 | 11.50 | 0.0991 | 0.0784 |
| 0.84 - 0.80 | 2138 | 2138 | 100.0 | 3.72 | 8.38 | 9.38 | 0.1212 | 0.0978 |
| 0.80 - 0.78 | 1196 | 1196 | 100.0 | 3.61 | 7.60 | 8.33 | 0.1297 | 0.1124 |
| 0.78 - 0.75 | 2124 | 2125 | 100.0 | 3.44 | 6.56 | 7.06 | 0.1507 | 0.1363 |
| 0.75 - 0.73 | 1523 | 1526 | 99.8 | 3.34 | 5.99 | 6.22 | 0.1654 | 0.1560 |
| 0.73 - 0.71 | 1820 | 1824 | 99.8 | 3.18 | 5.08 | 5.16 | 0.1930 | 0.1932 |
| 0.71 - 0.69 | 2001 | 2011 | 99.5 | 3.09 | 4.54 | 4.57 | 0.2137 | 0.2242 |
| 0.69 - 0.68 | 1058 | 1062 | 99.6 | 2.94 | 3.82 | 3.75 | 0.2465 | 0.2793 |
| 0.68 - 0.66 | 2335 | 2357 | 99.1 | 2.91 | 3.39 | 3.31 | 0.2691 | 0.3197 |
| 0.66 - 0.65 | 1281 | 1301 | 98.5 | 2.76 | 2.89 | 2.79 | 0.3119 | 0.3930 |
| 0.65 - 0.64 | 1372 | 1390 | 98.7 | 2.75 | 2.75 | 2.59 | 0.3306 | 0.4202 |
| 0.64 - 0.63 | 1425 | 1457 | 97.8 | 2.66 | 2.37 | 2.21 | 0.3613 | 0.4982 |
| 0.63 - 0.62 | 1560 | 1827 | 85.4 | 2.07 | 2.16 | 1.85 | 0.3920 | 0.6171 |
| 0.72 - 0.62 | 11905 | 12281 | 96.9 | 2.77 | 3.31 | 3.21 | 0.2735 | 0.3388 |
| Inf - 0.62 | 33267 | 33648 | 98.9 | 4.56 | 13.59 | 16.58 | 0.0544 | 0.0645 |

Complete .cif-data of the compound are available under the CCDC number CCDC-**2214322**.

A resolution cut off (SHEL 999 0.68) was applied to exclude reflections with poor signal to noise ratio at higher 2Θ angles. One aromatic ring of the anionic BAr$^F$ species shows a disorder over two positions with a fixed occupancy of 75:25%. All minor parts have been described using isotropic atomic displacement parameters. The AFIX 66 instruction was applied to force six membered aromatic rings into a hexagonal shape.

Four of the eight terminal $–CF_3$ groups of the $BAr^F$ anion show rotational disorder over two or more positions. The occupancies of F atoms were fixed at 25:75% or 20:20:60%. An ISOR instruction (ISOR 0.001 0.002) was used to treat the atomic displacement parameters of F1A F2A F3A and F13A F15A F14A. DFIX (1.34 0.001) was used to equalize the C–F bond lengths of disordered F1B F2B F3B and F1C F2C F3C attached to C53.

An additional positional disorder could be found in the 1,2-difluorobenzene solute molecule and it was refined with fixed occupancy of 75:25%. In both parts, AFIX 66 was used to force aromatic rings into hexagons.

Hydrogen atoms were placed in geometrically calculated positions and refined using an appropriate riding model. Hydrogen atoms at C9 and C10 were found by inspection of the residual electron density map during later refinement cycles. This finding is in accordance with short N–C (N1–C7 = 1.289 Å; N2–C8 = 1.291 Å) distances and confirms the presence of two N=C double bonds in the solid state.

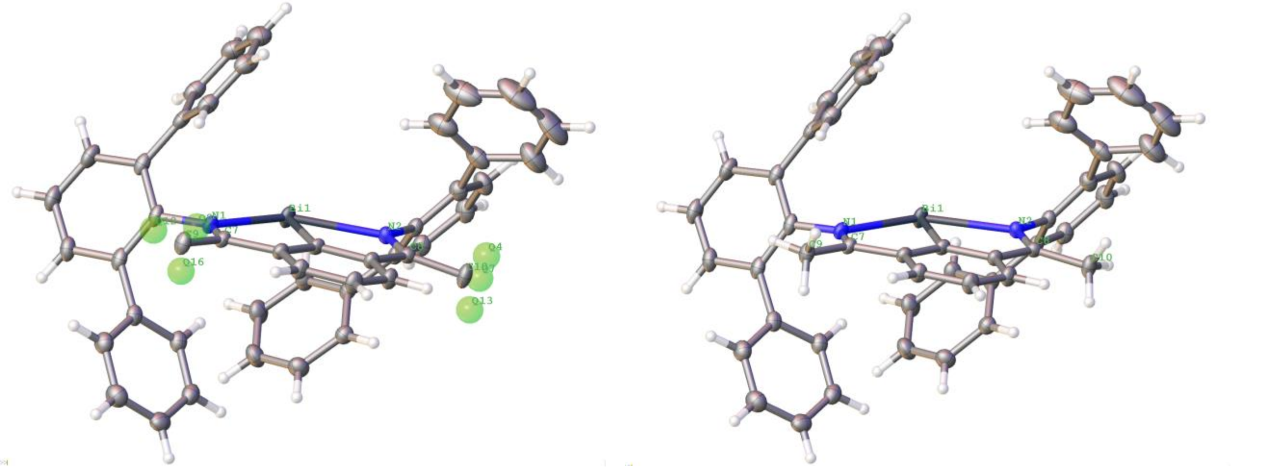


Figure S37. Solid-state structure of the cationic Bi(II) unit of compound **4**. Hydrogen atom positions attached to C9 and C10 methyl groups could be observed as residual electron density peaks (green) during later refinement.

Table S11**.** Crystal data and structure refinement of compound **4**·1,2-difluorobenzene solvate

| | | |
|---|---|---|
| Identification code | 14700 | |
| Empirical formula | $C_{84} H_{51} B Bi F_{26} N_2$ | |
| Color | dark orange | |
| Formula weight | 1802.06 g·mol$^{-1}$ | |
| Temperature | 100(2) K | |
| Wavelength | 0.71073 Å | |
| Crystal system | Triclinic | |
| Space group | *P*-1, (no. 2) | |
| Unit cell dimensions | a = 13.8650(7) Å | α= 102.879(2)°. |
| | b = 16.2579(8) Å | β= 92.613(2)°. |
| | c = 17.5527(9) Å | γ = 98.564(2)°. |
| Volume | 3801.4(3) Å$^3$ | |
| Z | 2 | |
| Density (calculated) | 1.574 Mg·m$^{-3}$ | |
| Absorption coefficient | 2.430 mm$^{-1}$ | |
| F(000) | 1782 e | |
| Crystal size | 0.278 x 0.074 x 0.068 mm$^3$ | |
| θ range for data collection | 1.194 to 31.506°. | |
| Index ranges | -20 ≤ h ≤ 19, -23≤ k ≤ 23, -25 ≤ l ≤ 25 | |
| Reflections collected | 131508 | |
| Independent reflections | 25294 [$R_{int}$ = 0.0493] | |
| Reflections with I>2σ(I) | 20187 | |
| Completeness to θ = 25.242° | 100.0 % | |
| Absorption correction | Gaussian | |
| Max. and min. transmission | 0.90078 and 0.76934 | |
| Refinement method | Full-matrix least-squares on $F^2$ | |
| Data / restraints / parameters | 25294 / 42 / 1145 | |
| Goodness-of-fit on $F^2$ | 1.020 | |
| Final R indices [I>2σ(I)] | $R_1$ = 0.0347 | $wR^2$ = 0.0699 |
| R indices (all data) | $R_1$ = 0.0552 | $wR^2$ = 0.0755 |
| Extinction coefficient | n/a | |
| Largest diff. peak and hole | 1.323 and -1.221 e·Å$^{-3}$ | |

Table S12. Bond lengths [Å] and angles [°] of compound **4**·1,2-difluorobenzene solvate

| | | | |
|---|---|---|---|
| Bi(1)-N(1) | 2.4569(18) | Bi(1)-N(2) | 2.4786(17) |
| Bi(1)-C(1) | 2.1809(19) | N(1)-C(7) | 1.290(3) |
| N(1)-C(11) | 1.427(3) | N(2)-C(8) | 1.291(3) |
| N(2)-C(29) | 1.429(3) | C(1)-C(2) | 1.391(3) |
| C(1)-C(6) | 1.397(3) | C(2)-C(3) | 1.397(3) |
| C(2)-C(7) | 1.480(3) | C(3)-H(3) | 0.9500 |
| C(3)-C(4) | 1.385(3) | C(4)-H(4) | 0.9500 |
| C(4)-C(5) | 1.391(3) | C(5)-H(5) | 0.9500 |
| C(5)-C(6) | 1.395(3) | C(6)-C(8) | 1.477(3) |
| C(7)-C(9) | 1.493(3) | C(8)-C(10) | 1.496(3) |
| C(9)-H(9A) | 0.9800 | C(9)-H(9B) | 0.9800 |
| C(9)-H(9C) | 0.9800 | C(10)-H(10A) | 0.9800 |
| C(10)-H(10B) | 0.9800 | C(10)-H(10C) | 0.9800 |
| C(11)-C(12) | 1.400(3) | C(11)-C(16) | 1.404(3) |
| C(12)-C(13) | 1.392(3) | C(12)-C(17) | 1.488(3) |
| C(13)-H(13) | 0.9500 | C(13)-C(14) | 1.386(4) |
| C(14)-H(14) | 0.9500 | C(14)-C(15) | 1.379(4) |
| C(15)-H(15) | 0.9500 | C(15)-C(16) | 1.398(3) |
| C(16)-C(23) | 1.479(4) | C(17)-C(18) | 1.398(3) |
| C(17)-C(22) | 1.386(3) | C(18)-H(18) | 0.9500 |
| C(18)-C(19) | 1.375(3) | C(19)-H(19) | 0.9500 |
| C(19)-C(20) | 1.385(4) | C(20)-H(20) | 0.9500 |
| C(20)-C(21) | 1.381(4) | C(21)-H(21) | 0.9500 |
| C(21)-C(22) | 1.386(4) | C(22)-H(22) | 0.9500 |
| C(23)-C(24) | 1.399(3) | C(23)-C(28) | 1.397(3) |
| C(24)-H(24) | 0.9500 | C(24)-C(25) | 1.378(4) |
| C(25)-H(25) | 0.9500 | C(25)-C(26) | 1.389(4) |
| C(26)-H(26) | 0.9500 | C(26)-C(27) | 1.387(4) |
| C(27)-H(27) | 0.9500 | C(27)-C(28) | 1.377(4) |
| C(28)-H(28) | 0.9500 | C(29)-C(30) | 1.400(3) |
| C(29)-C(34) | 1.405(3) | C(30)-C(31) | 1.393(3) |
| C(30)-C(35) | 1.482(3) | C(31)-H(31) | 0.9500 |
| C(31)-C(32) | 1.377(3) | C(32)-H(32) | 0.9500 |
| C(32)-C(33) | 1.376(4) | C(33)-H(33) | 0.9500 |

| | | | |
|---|---|---|---|
| C(33)-C(34) | 1.395(3) | C(34)-C(41) | 1.487(4) |
| C(35)-C(36) | 1.399(3) | C(35)-C(40) | 1.395(3) |
| C(36)-H(36) | 0.9500 | C(36)-C(37) | 1.379(3) |
| C(37)-H(37) | 0.9500 | C(37)-C(38) | 1.381(4) |
| C(38)-H(38) | 0.9500 | C(38)-C(39) | 1.395(3) |
| C(39)-H(39) | 0.9500 | C(39)-C(40) | 1.378(3) |
| C(40)-H(40) | 0.9500 | C(41)-C(42) | 1.394(4) |
| C(41)-C(46) | 1.383(4) | C(42)-H(42) | 0.9500 |
| C(42)-C(43) | 1.387(5) | C(43)-H(43) | 0.9500 |
| C(43)-C(44) | 1.377(6) | C(44)-H(44) | 0.9500 |
| C(44)-C(45) | 1.381(6) | C(45)-H(45) | 0.9500 |
| C(45)-C(46) | 1.392(4) | C(46)-H(46) | 0.9500 |
| F(1A)-C(53) | 1.300(4) | F(1B)-C(53) | 1.3419(10) |
| F(1C)-C(53) | 1.3407(10) | F(2A)-C(53) | 1.321(5) |
| F(2B)-C(53) | 1.3401(10) | F(2C)-C(53) | 1.3391(10) |
| F(3A)-C(53) | 1.429(5) | F(3B)-C(53) | 1.3390(10) |
| F(3C)-C(53) | 1.3397(10) | F(4A)-C(54) | 1.323(4) |
| F(4B)-C(54) | 1.414(13) | F(5A)-C(54) | 1.335(6) |
| F(5B)-C(54) | 1.303(18) | F(6A)-C(54) | 1.371(4) |
| F(6B)-C(54) | 1.213(10) | F(7)-C(61) | 1.333(3) |
| F(8)-C(61) | 1.335(3) | F(9)-C(61) | 1.329(3) |
| F(10)-C(62) | 1.325(3) | F(11)-C(62) | 1.341(3) |
| F(12)-C(62) | 1.331(3) | F(13A)-C(69) | 1.326(4) |
| F(13B)-C(69) | 1.362(12) | F(13C)-C(69) | 1.413(12) |
| F(14A)-C(69) | 1.353(4) | F(14B)-C(69) | 1.450(13) |
| F(14C)-C(69) | 1.232(10) | F(15A)-C(69) | 1.365(4) |
| F(15B)-C(69) | 1.250(13) | F(15C)-C(69) | 1.362(13) |
| F(16)-C(70) | 1.329(3) | F(17)-C(70) | 1.329(4) |
| F(18)-C(70) | 1.328(3) | F(19A)-C(77A) | 1.322(5) |
| F(19B)-C(77B) | 1.34(2) | F(20A)-C(77A) | 1.352(5) |
| F(20B)-C(77B) | 1.264(19) | F(21A)-C(77A) | 1.331(5) |
| F(21B)-C(77B) | 1.292(18) | F(22A)-C(78) | 1.374(4) |
| F(22B)-C(78) | 1.265(11) | F(22C)-C(78) | 1.330(14) |
| F(23A)-C(78) | 1.331(4) | F(23B)-C(78) | 1.394(13) |
| F(23C)-C(78) | 1.285(12) | F(24A)-C(78) | 1.312(4) |
| F(24B)-C(78) | 1.368(12) | F(24C)-C(78) | 1.368(13) |

| | | | |
|---|---|---|---|
| C(47)-C(48) | 1.392(3) | C(47)-C(52) | 1.396(3) |
| C(47)-B(1) | 1.638(3) | C(48)-H(48) | 0.9500 |
| C(48)-C(49) | 1.387(3) | C(49)-C(50) | 1.384(3) |
| C(49)-C(53) | 1.494(3) | C(50)-H(50) | 0.9500 |
| C(50)-C(51) | 1.379(3) | C(51)-C(52) | 1.391(3) |
| C(51)-C(54) | 1.489(3) | C(52)-H(52) | 0.9500 |
| C(55)-C(56) | 1.399(3) | C(55)-C(60) | 1.393(3) |
| C(55)-B(1) | 1.642(3) | C(56)-H(56) | 0.9500 |
| C(56)-C(57) | 1.392(3) | C(57)-C(58) | 1.377(3) |
| C(57)-C(61) | 1.493(3) | C(58)-H(58) | 0.9500 |
| C(58)-C(59) | 1.393(3) | C(59)-C(60) | 1.388(3) |
| C(59)-C(62) | 1.485(4) | C(60)-H(60) | 0.9500 |
| C(63)-C(64) | 1.399(3) | C(63)-C(68) | 1.400(3) |
| C(63)-B(1) | 1.631(3) | C(64)-H(64) | 0.9500 |
| C(64)-C(65) | 1.389(4) | C(65)-C(66) | 1.380(4) |
| C(65)-C(70) | 1.500(4) | C(66)-H(66) | 0.9500 |
| C(66)-C(67) | 1.378(4) | C(67)-C(68) | 1.389(3) |
| C(67)-C(69) | 1.492(4) | C(68)-H(68) | 0.9500 |
| C(71)-C(72B) | 1.3900 | C(71)-C(76B) | 1.3900 |
| C(71)-C(72A) | 1.404(4) | C(71)-C(76A) | 1.405(4) |
| C(71)-B(1) | 1.637(3) | C(72B)-H(72B) | 0.9500 |
| C(72B)-C(73B) | 1.3900 | C(73B)-C(74B) | 1.3900 |
| C(73B)-C(77B) | 1.543(18) | C(74B)-H(74B) | 0.9500 |
| C(74B)-C(75B) | 1.3900 | C(75B)-C(76B) | 1.3900 |
| C(75B)-C(78) | 1.481(5) | C(76B)-H(76B) | 0.9500 |
| C(72A)-H(72A) | 0.9500 | C(72A)-C(73A) | 1.390(4) |
| C(73A)-C(74A) | 1.386(4) | C(73A)-C(77A) | 1.489(5) |
| C(74A)-H(74A) | 0.9500 | C(74A)-C(75A) | 1.377(7) |
| C(75A)-C(76A) | 1.391(6) | C(75A)-C(78) | 1.505(4) |
| C(76A)-H(76A) | 0.9500 | F(25A)-C(79A) | 1.320(4) |
| F(26A)-C(84A) | 1.325(4) | C(79A)-C(84A) | 1.3900 |
| C(79A)-C(80A) | 1.3900 | C(84A)-C(83A) | 1.3900 |
| C(83A)-H(83A) | 0.9500 | C(83A)-C(82A) | 1.3900 |
| C(82A)-H(82A) | 0.9500 | C(82A)-C(81A) | 1.3900 |
| C(81A)-H(81A) | 0.9500 | C(81A)-C(80A) | 1.3900 |
| C(80A)-H(80A) | 0.9500 | F(25B)-C(79B) | 1.361(16) |

| | | | |
|---|---|---|---|
| F(26B)-C(84B) | 1.314(19) | C(84B)-C(83B) | 1.3900 |
| C(84B)-C(79B) | 1.3900 | C(83B)-H(83B) | 0.9500 |
| C(83B)-C(82B) | 1.3900 | C(82B)-H(82B) | 0.9500 |
| C(82B)-C(81B) | 1.3900 | C(81B)-H(81B) | 0.9500 |
| C(81B)-C(80B) | 1.3900 | C(80B)-H(80B) | 0.9500 |
| C(80B)-C(79B) | 1.3900 | | |
| | | | |
| N(1)-Bi(1)-N(2) | 142.39(6) | C(1)-Bi(1)-N(1) | 71.13(7) |
| C(1)-Bi(1)-N(2) | 71.36(7) | C(7)-N(1)-Bi(1) | 115.02(14) |
| C(7)-N(1)-C(11) | 123.50(18) | C(11)-N(1)-Bi(1) | 121.42(13) |
| C(8)-N(2)-Bi(1) | 114.05(14) | C(8)-N(2)-C(29) | 123.67(18) |
| C(29)-N(2)-Bi(1) | 122.14(12) | C(2)-C(1)-Bi(1) | 119.29(14) |
| C(2)-C(1)-C(6) | 121.58(18) | C(6)-C(1)-Bi(1) | 119.13(15) |
| C(1)-C(2)-C(3) | 118.90(19) | C(1)-C(2)-C(7) | 118.48(18) |
| C(3)-C(2)-C(7) | 122.57(19) | C(2)-C(3)-H(3) | 120.0 |
| C(4)-C(3)-C(2) | 120.1(2) | C(4)-C(3)-H(3) | 120.0 |
| C(3)-C(4)-H(4) | 119.7 | C(3)-C(4)-C(5) | 120.62(19) |
| C(5)-C(4)-H(4) | 119.7 | C(4)-C(5)-H(5) | 119.9 |
| C(4)-C(5)-C(6) | 120.2(2) | C(6)-C(5)-H(5) | 119.9 |
| C(1)-C(6)-C(8) | 118.75(18) | C(5)-C(6)-C(1) | 118.6(2) |
| C(5)-C(6)-C(8) | 122.66(19) | N(1)-C(7)-C(2) | 115.90(18) |
| N(1)-C(7)-C(9) | 124.35(19) | C(2)-C(7)-C(9) | 119.74(17) |
| N(2)-C(8)-C(6) | 116.57(18) | N(2)-C(8)-C(10) | 124.27(19) |
| C(6)-C(8)-C(10) | 119.09(18) | C(7)-C(9)-H(9A) | 109.5 |
| C(7)-C(9)-H(9B) | 109.5 | C(7)-C(9)-H(9C) | 109.5 |
| H(9A)-C(9)-H(9B) | 109.5 | H(9A)-C(9)-H(9C) | 109.5 |
| H(9B)-C(9)-H(9C) | 109.5 | C(8)-C(10)-H(10A) | 109.5 |
| C(8)-C(10)-H(10B) | 109.5 | C(8)-C(10)-H(10C) | 109.5 |
| H(10A)-C(10)-H(10B) | 109.5 | H(10A)-C(10)-H(10C) | 109.5 |
| H(10B)-C(10)-H(10C) | 109.5 | C(12)-C(11)-N(1) | 120.2(2) |
| C(12)-C(11)-C(16) | 121.51(19) | C(16)-C(11)-N(1) | 118.0(2) |
| C(11)-C(12)-C(17) | 122.40(19) | C(13)-C(12)-C(11) | 118.0(2) |
| C(13)-C(12)-C(17) | 119.5(2) | C(12)-C(13)-H(13) | 119.3 |
| C(14)-C(13)-C(12) | 121.5(2) | C(14)-C(13)-H(13) | 119.3 |
| C(13)-C(14)-H(14) | 120.2 | C(15)-C(14)-C(13) | 119.6(2) |
| C(15)-C(14)-H(14) | 120.2 | C(14)-C(15)-H(15) | 119.4 |

| | | | |
|---|---|---|---|
| C(14)-C(15)-C(16) | 121.3(2) | C(16)-C(15)-H(15) | 119.4 |
| C(11)-C(16)-C(23) | 120.69(19) | C(15)-C(16)-C(11) | 118.1(2) |
| C(15)-C(16)-C(23) | 121.3(2) | C(18)-C(17)-C(12) | 120.8(2) |
| C(22)-C(17)-C(12) | 120.4(2) | C(22)-C(17)-C(18) | 118.7(2) |
| C(17)-C(18)-H(18) | 119.8 | C(19)-C(18)-C(17) | 120.4(2) |
| C(19)-C(18)-H(18) | 119.8 | C(18)-C(19)-H(19) | 119.7 |
| C(18)-C(19)-C(20) | 120.6(2) | C(20)-C(19)-H(19) | 119.7 |
| C(19)-C(20)-H(20) | 120.2 | C(21)-C(20)-C(19) | 119.5(2) |
| C(21)-C(20)-H(20) | 120.2 | C(20)-C(21)-H(21) | 120.0 |
| C(20)-C(21)-C(22) | 120.1(2) | C(22)-C(21)-H(21) | 120.0 |
| C(17)-C(22)-C(21) | 120.7(2) | C(17)-C(22)-H(22) | 119.6 |
| C(21)-C(22)-H(22) | 119.6 | C(24)-C(23)-C(16) | 120.3(2) |
| C(28)-C(23)-C(16) | 121.6(2) | C(28)-C(23)-C(24) | 118.1(2) |
| C(23)-C(24)-H(24) | 119.6 | C(25)-C(24)-C(23) | 120.8(2) |
| C(25)-C(24)-H(24) | 119.6 | C(24)-C(25)-H(25) | 119.8 |
| C(24)-C(25)-C(26) | 120.4(2) | C(26)-C(25)-H(25) | 119.8 |
| C(25)-C(26)-H(26) | 120.3 | C(27)-C(26)-C(25) | 119.4(3) |
| C(27)-C(26)-H(26) | 120.3 | C(26)-C(27)-H(27) | 119.9 |
| C(28)-C(27)-C(26) | 120.3(2) | C(28)-C(27)-H(27) | 119.9 |
| C(23)-C(28)-H(28) | 119.5 | C(27)-C(28)-C(23) | 121.0(2) |
| C(27)-C(28)-H(28) | 119.5 | C(30)-C(29)-N(2) | 117.42(19) |
| C(30)-C(29)-C(34) | 120.89(19) | C(34)-C(29)-N(2) | 121.4(2) |
| C(29)-C(30)-C(35) | 121.92(19) | C(31)-C(30)-C(29) | 118.8(2) |
| C(31)-C(30)-C(35) | 119.2(2) | C(30)-C(31)-H(31) | 119.5 |
| C(32)-C(31)-C(30) | 120.9(2) | C(32)-C(31)-H(31) | 119.5 |
| C(31)-C(32)-H(32) | 120.1 | C(33)-C(32)-C(31) | 119.8(2) |
| C(33)-C(32)-H(32) | 120.1 | C(32)-C(33)-H(33) | 119.1 |
| C(32)-C(33)-C(34) | 121.7(2) | C(34)-C(33)-H(33) | 119.1 |
| C(29)-C(34)-C(41) | 123.3(2) | C(33)-C(34)-C(29) | 117.8(2) |
| C(33)-C(34)-C(41) | 118.8(2) | C(36)-C(35)-C(30) | 118.9(2) |
| C(40)-C(35)-C(30) | 122.52(19) | C(40)-C(35)-C(36) | 118.5(2) |
| C(35)-C(36)-H(36) | 119.6 | C(37)-C(36)-C(35) | 120.7(2) |
| C(37)-C(36)-H(36) | 119.6 | C(36)-C(37)-H(37) | 119.8 |
| C(36)-C(37)-C(38) | 120.3(2) | C(38)-C(37)-H(37) | 119.8 |
| C(37)-C(38)-H(38) | 120.2 | C(37)-C(38)-C(39) | 119.6(2) |
| C(39)-C(38)-H(38) | 120.2 | C(38)-C(39)-H(39) | 119.9 |

| | | | |
|---|---|---|---|
| C(40)-C(39)-C(38) | 120.2(2) | C(40)-C(39)-H(39) | 119.9 |
| C(35)-C(40)-H(40) | 119.7 | C(39)-C(40)-C(35) | 120.7(2) |
| C(39)-C(40)-H(40) | 119.7 | C(42)-C(41)-C(34) | 119.6(3) |
| C(46)-C(41)-C(34) | 121.6(3) | C(46)-C(41)-C(42) | 118.8(3) |
| C(41)-C(42)-H(42) | 119.6 | C(43)-C(42)-C(41) | 120.8(4) |
| C(43)-C(42)-H(42) | 119.6 | C(42)-C(43)-H(43) | 120.1 |
| C(44)-C(43)-C(42) | 119.7(4) | C(44)-C(43)-H(43) | 120.1 |
| C(43)-C(44)-H(44) | 119.9 | C(43)-C(44)-C(45) | 120.2(3) |
| C(45)-C(44)-H(44) | 119.9 | C(44)-C(45)-H(45) | 120.0 |
| C(44)-C(45)-C(46) | 120.0(4) | C(46)-C(45)-H(45) | 120.0 |
| C(41)-C(46)-C(45) | 120.4(3) | C(41)-C(46)-H(46) | 119.8 |
| C(45)-C(46)-H(46) | 119.8 | C(48)-C(47)-C(52) | 115.63(19) |
| C(48)-C(47)-B(1) | 122.29(19) | C(52)-C(47)-B(1) | 121.82(19) |
| C(47)-C(48)-H(48) | 119.0 | C(49)-C(48)-C(47) | 122.0(2) |
| C(49)-C(48)-H(48) | 119.0 | C(48)-C(49)-C(53) | 119.6(2) |
| C(50)-C(49)-C(48) | 121.4(2) | C(50)-C(49)-C(53) | 119.0(2) |
| C(49)-C(50)-H(50) | 121.2 | C(51)-C(50)-C(49) | 117.7(2) |
| C(51)-C(50)-H(50) | 121.2 | C(50)-C(51)-C(52) | 120.7(2) |
| C(50)-C(51)-C(54) | 120.7(2) | C(52)-C(51)-C(54) | 118.7(2) |
| C(47)-C(52)-H(52) | 118.7 | C(51)-C(52)-C(47) | 122.6(2) |
| C(51)-C(52)-H(52) | 118.7 | F(1A)-C(53)-F(2A) | 116.1(4) |
| F(1A)-C(53)-F(3A) | 106.5(4) | F(1A)-C(53)-C(49) | 114.5(2) |
| F(1B)-C(53)-C(49) | 105.7(5) | F(1C)-C(53)-C(49) | 115.8(5) |
| F(2A)-C(53)-F(3A) | 96.7(4) | F(2A)-C(53)-C(49) | 113.6(4) |
| F(2B)-C(53)-F(1B) | 85.3(10) | F(2B)-C(53)-C(49) | 110.0(7) |
| F(2C)-C(53)-F(1C) | 109.2(7) | F(2C)-C(53)-F(3C) | 106.4(8) |
| F(2C)-C(53)-C(49) | 118.4(5) | F(3A)-C(53)-C(49) | 107.0(3) |
| F(3B)-C(53)-F(1B) | 106.2(9) | F(3B)-C(53)-F(2B) | 121.8(10) |
| F(3B)-C(53)-C(49) | 120.1(7) | F(3C)-C(53)-F(1C) | 85.3(10) |
| F(3C)-C(53)-C(49) | 116.8(8) | F(4A)-C(54)-F(5A) | 108.6(4) |
| F(4A)-C(54)-F(6A) | 104.5(3) | F(4A)-C(54)-C(51) | 112.7(2) |
| F(4B)-C(54)-C(51) | 106.9(5) | F(5A)-C(54)-F(6A) | 106.3(3) |
| F(5A)-C(54)-C(51) | 113.4(3) | F(5B)-C(54)-F(4B) | 99.4(9) |
| F(5B)-C(54)-C(51) | 115.1(8) | F(6A)-C(54)-C(51) | 110.9(2) |
| F(6B)-C(54)-F(4B) | 105.6(6) | F(6B)-C(54)-F(5B) | 109.5(9) |
| F(6B)-C(54)-C(51) | 118.1(5) | C(56)-C(55)-B(1) | 122.93(19) |

| | | | |
|---|---|---|---|
| C(60)-C(55)-C(56) | 115.52(19) | C(60)-C(55)-B(1) | 121.44(19) |
| C(55)-C(56)-H(56) | 118.9 | C(57)-C(56)-C(55) | 122.2(2) |
| C(57)-C(56)-H(56) | 118.9 | C(56)-C(57)-C(61) | 118.6(2) |
| C(58)-C(57)-C(56) | 121.1(2) | C(58)-C(57)-C(61) | 120.3(2) |
| C(57)-C(58)-H(58) | 121.1 | C(57)-C(58)-C(59) | 117.8(2) |
| C(59)-C(58)-H(58) | 121.1 | C(58)-C(59)-C(62) | 118.8(2) |
| C(60)-C(59)-C(58) | 120.6(2) | C(60)-C(59)-C(62) | 120.5(2) |
| C(55)-C(60)-H(60) | 118.7 | C(59)-C(60)-C(55) | 122.6(2) |
| C(59)-C(60)-H(60) | 118.7 | F(7)-C(61)-F(8) | 106.21(19) |
| F(7)-C(61)-C(57) | 112.3(2) | F(8)-C(61)-C(57) | 113.0(2) |
| F(9)-C(61)-F(7) | 105.8(2) | F(9)-C(61)-F(8) | 106.3(2) |
| F(9)-C(61)-C(57) | 112.75(19) | F(10)-C(62)-F(11) | 105.7(2) |
| F(10)-C(62)-F(12) | 106.7(2) | F(10)-C(62)-C(59) | 113.6(2) |
| F(11)-C(62)-C(59) | 111.8(2) | F(12)-C(62)-F(11) | 105.5(2) |
| F(12)-C(62)-C(59) | 112.9(2) | C(64)-C(63)-C(68) | 115.3(2) |
| C(64)-C(63)-B(1) | 123.03(19) | C(68)-C(63)-B(1) | 121.54(19) |
| C(63)-C(64)-H(64) | 118.9 | C(65)-C(64)-C(63) | 122.3(2) |
| C(65)-C(64)-H(64) | 118.9 | C(64)-C(65)-C(70) | 118.9(2) |
| C(66)-C(65)-C(64) | 120.8(2) | C(66)-C(65)-C(70) | 120.2(2) |
| C(65)-C(66)-H(66) | 120.8 | C(67)-C(66)-C(65) | 118.4(2) |
| C(67)-C(66)-H(66) | 120.8 | C(66)-C(67)-C(68) | 120.6(2) |
| C(66)-C(67)-C(69) | 119.7(2) | C(68)-C(67)-C(69) | 119.5(2) |
| C(63)-C(68)-H(68) | 118.7 | C(67)-C(68)-C(63) | 122.6(2) |
| C(67)-C(68)-H(68) | 118.7 | F(13A)-C(69)-F(14A) | 106.6(3) |
| F(13A)-C(69)-F(15A) | 105.6(3) | F(13A)-C(69)-C(67) | 114.8(3) |
| F(13B)-C(69)-F(14B) | 99.1(7) | F(13B)-C(69)-C(67) | 112.2(5) |
| F(13C)-C(69)-C(67) | 106.5(5) | F(14A)-C(69)-F(15A) | 104.4(3) |
| F(14A)-C(69)-C(67) | 113.1(2) | F(14B)-C(69)-C(67) | 113.6(6) |
| F(14C)-C(69)-F(13C) | 108.6(7) | F(14C)-C(69)-F(15C) | 110.0(7) |
| F(14C)-C(69)-C(67) | 110.5(5) | F(15A)-C(69)-C(67) | 111.6(2) |
| F(15B)-C(69)-F(13B) | 111.3(8) | F(15B)-C(69)-F(14B) | 107.9(7) |
| F(15B)-C(69)-C(67) | 112.1(6) | F(15C)-C(69)-F(13C) | 109.3(7) |
| F(15C)-C(69)-C(67) | 111.8(6) | F(16)-C(70)-F(17) | 106.4(2) |
| F(16)-C(70)-C(65) | 112.9(3) | F(17)-C(70)-C(65) | 112.7(2) |
| F(18)-C(70)-F(16) | 107.0(3) | F(18)-C(70)-F(17) | 105.6(3) |
| F(18)-C(70)-C(65) | 111.7(2) | C(72B)-C(71)-C(76B) | 120.0 |

| | | | |
|---|---|---|---|
| C(72B)-C(71)-B(1) | 123.0(3) | C(76B)-C(71)-B(1) | 116.5(3) |
| C(72A)-C(71)-C(76A) | 114.5(3) | C(72A)-C(71)-B(1) | 124.8(2) |
| C(76A)-C(71)-B(1) | 120.6(3) | C(71)-C(72B)-H(72B) | 120.0 |
| C(73B)-C(72B)-C(71) | 120.0 | C(73B)-C(72B)-H(72B) | 120.0 |
| C(72B)-C(73B)-C(74B) | 120.0 | C(72B)-C(73B)-C(77B) | 119.2(8) |
| C(74B)-C(73B)-C(77B) | 120.7(8) | C(73B)-C(74B)-H(74B) | 120.0 |
| C(75B)-C(74B)-C(73B) | 120.0 | C(75B)-C(74B)-H(74B) | 120.0 |
| C(74B)-C(75B)-C(76B) | 120.0 | C(74B)-C(75B)-C(78) | 115.7(6) |
| C(76B)-C(75B)-C(78) | 124.2(6) | C(71)-C(76B)-H(76B) | 120.0 |
| C(75B)-C(76B)-C(71) | 120.0 | C(75B)-C(76B)-H(76B) | 120.0 |
| C(71)-C(72A)-H(72A) | 118.6 | C(73A)-C(72A)-C(71) | 122.9(3) |
| C(73A)-C(72A)-H(72A) | 118.6 | C(72A)-C(73A)-C(77A) | 118.7(3) |
| C(74A)-C(73A)-C(72A) | 120.9(3) | C(74A)-C(73A)-C(77A) | 120.4(3) |
| C(73A)-C(74A)-H(74A) | 121.1 | C(75A)-C(74A)-C(73A) | 117.8(3) |
| C(75A)-C(74A)-H(74A) | 121.1 | C(74A)-C(75A)-C(76A) | 121.2(3) |
| C(74A)-C(75A)-C(78) | 121.6(4) | C(76A)-C(75A)-C(78) | 117.2(5) |
| C(71)-C(76A)-H(76A) | 118.7 | C(75A)-C(76A)-C(71) | 122.7(3) |
| C(75A)-C(76A)-H(76A) | 118.7 | F(19A)-C(77A)-F(20A) | 105.9(3) |
| F(19A)-C(77A)-F(21A) | 107.3(3) | F(19A)-C(77A)-C(73A) | 114.1(4) |
| F(20A)-C(77A)-C(73A) | 111.1(3) | F(21A)-C(77A)-F(20A) | 105.4(4) |
| F(21A)-C(77A)-C(73A) | 112.5(3) | F(19B)-C(77B)-C(73B) | 109.2(14) |
| F(20B)-C(77B)-F(19B) | 116.3(14) | F(20B)-C(77B)-F(21B) | 101.9(15) |
| F(20B)-C(77B)-C(73B) | 113.2(13) | F(21B)-C(77B)-F(19B) | 102.7(12) |
| F(21B)-C(77B)-C(73B) | 112.9(12) | F(22A)-C(78)-C(75A) | 109.8(4) |
| F(22B)-C(78)-F(23B) | 105.0(7) | F(22B)-C(78)-F(24B) | 103.7(7) |
| F(22B)-C(78)-C(75B) | 113.7(10) | F(22C)-C(78)-F(24C) | 101.9(8) |
| F(23A)-C(78)-F(22A) | 106.9(3) | F(23A)-C(78)-C(75A) | 111.7(3) |
| F(23B)-C(78)-C(75B) | 115.0(8) | F(23C)-C(78)-F(22C) | 103.6(8) |
| F(23C)-C(78)-F(24C) | 109.4(7) | F(24A)-C(78)-F(22A) | 106.5(3) |
| F(24A)-C(78)-F(23A) | 108.7(4) | F(24A)-C(78)-C(75A) | 113.0(3) |
| F(24B)-C(78)-F(23B) | 105.1(7) | F(24B)-C(78)-C(75B) | 113.3(6) |
| C(47)-B(1)-C(55) | 103.77(16) | C(63)-B(1)-C(47) | 112.57(18) |
| C(63)-B(1)-C(55) | 111.93(17) | C(63)-B(1)-C(71) | 106.15(16) |
| C(71)-B(1)-C(47) | 109.90(17) | C(71)-B(1)-C(55) | 112.67(18) |
| F(25A)-C(79A)-C(84A) | 115.4(3) | F(25A)-C(79A)-C(80A) | 124.6(3) |
| C(84A)-C(79A)-C(80A) | 120.0 | F(26A)-C(84A)-C(79A) | 121.7(3) |

| | | | |
|---|---|---|---|
| F(26A)-C(84A)-C(83A) | 118.3(3) | C(83A)-C(84A)-C(79A) | 120.0 |
| C(84A)-C(83A)-H(83A) | 120.0 | C(84A)-C(83A)-C(82A) | 120.0 |
| C(82A)-C(83A)-H(83A) | 120.0 | C(83A)-C(82A)-H(82A) | 120.0 |
| C(81A)-C(82A)-C(83A) | 120.0 | C(81A)-C(82A)-H(82A) | 120.0 |
| C(82A)-C(81A)-H(81A) | 120.0 | C(82A)-C(81A)-C(80A) | 120.0 |
| C(80A)-C(81A)-H(81A) | 120.0 | C(79A)-C(80A)-H(80A) | 120.0 |
| C(81A)-C(80A)-C(79A) | 120.0 | C(81A)-C(80A)-H(80A) | 120.0 |
| F(26B)-C(84B)-C(83B) | 128.6(12) | F(26B)-C(84B)-C(79B) | 111.3(12) |
| C(83B)-C(84B)-C(79B) | 120.0 | C(84B)-C(83B)-H(83B) | 120.0 |
| C(82B)-C(83B)-C(84B) | 120.0 | C(82B)-C(83B)-H(83B) | 120.0 |
| C(83B)-C(82B)-H(82B) | 120.0 | C(83B)-C(82B)-C(81B) | 120.0 |
| C(81B)-C(82B)-H(82B) | 120.0 | C(82B)-C(81B)-H(81B) | 120.0 |
| C(82B)-C(81B)-C(80B) | 120.0 | C(80B)-C(81B)-H(81B) | 120.0 |
| C(81B)-C(80B)-H(80B) | 120.0 | C(79B)-C(80B)-C(81B) | 120.0 |
| C(79B)-C(80B)-H(80B) | 120.0 | F(25B)-C(79B)-C(84B) | 123.5(10) |
| F(25B)-C(79B)-C(80B) | 116.4(10) | C(80B)-C(79B)-C(84B) | 120.0 |


(1) (a) Pang, Y.; Leutzsch, M.; Nöthling, N.; Cornella, J. Catalytic activation of $N_2O$ at a low-valent bismuth redox platform. *J. Am. Chem. Soc.* **2020**, *142*, 19473-19479. (b) Chávez, I.; Alvarez-Carena, A.; Molins, E.; Roig, A.; Maniukiewicz, W.; Arancibia, A.; Arancibia, V.; Brand, H.; Manuel Manríquez, J. Selective oxidants for organometallic compounds containing a stabilising anion of highly reactive cations: $(3,5(CF_3)_2C_6H_3)_4B^-)Cp_2Fe^+$ and $(3,5(CF_3)_2C_6H_3)_4B^-)Cp^*{}_2Fe^+$. *J. Organomet. Chem.* **2000**, *601*, 126-132.

(2) Reijerse, E.; Lendzian, F.; Isaacson, R.; Lubitz, W. A tunable general purpose Q-band resonator for CW and pulse EPR/ENDOR experiments with large sample access and optical excitation. *J. Magn. Reson.* **2012**, *214*, 237-243.

(3) Hyde, J. S.; Pasenkiewicz-Gierula, M.; Jesmanowicz, A.; Antholine, W. E. Pseudo field modulation in EPR spectroscopy. *Appl Magn Reson* **1990**, *1*, 483.

(4) Stoll, S.; Schweiger, A. EasySpin, a comprehensive software package for spectral simulation and analysis in EPR. *J Magn Reson.* **2006**, *178*, 42-55.

(5) (a) Evans, D. F. 400. The determination of the paramagnetic susceptibility of substances in solution by nuclear magnetic resonance. *J. Chem. Soc.* **1959**, 2003-2005. (b) Schubert, E. M. Utilizing the Evans method with a superconducting NMR spectrometer in the undergraduate laboratory. *J. Chem. Educ.* **1992**, *69*, 62. (c) Piguet, C. Paramagnetic Susceptibility by NMR: The "Solvent Correction" Removed for Large Paramagnetic Molecules. *Journal of Chemical Education* **1997**, *74*, 815. (d) Ostfeld, D.; Cohen, I. A. A cautionary note on the use of the Evans method for magnetic moments. *J. Chem. Educ.* **1972**, *49*, 829.

(6) Bain, G. A.; Berry, J. F. Diamagnetic corrections and Pascal's constants. *J. Chem. Educ.* **2008**, *85*, 532-536.

(7) Morton, J. R.; Preston, K. F. Atomic parameters for paramagnetic resonance data. *J. Magn. Reson.* **1978**, *30*, 577-582.

(8) Schwamm, R. J.; Harmer, J. R.; Lein, M.; Fitchett, C. M.; Granville, S.; Coles, M. P. Isolation and characterization of a bismuth(II) radical. *Angew. Chem. Int. Ed.* **2015**, *54*, 10630-10633.

(9) Ganesamoorthy, C.; Helling, C.; Wölper, C.; Frank, W.; Bill, E.; Cutsail, G. E.; Schulz, S. From stable Sb- and Bi-centered radicals to a compound with a Ga=Sb double bond. *Nat. Commun.* **2018**, *9*, 87.
(10) Krüger, J.; Haak, J.; Wölper, C.; Cutsail, G. E.; Haberhauer, G.; Schulz, S. Single-electron oxidation of carbene-coordinated pnictinidenes–entry into heteroleptic radical cations and metalloid clusters. *Inorg. Chem.* **2022**, *61*, 5878-5884.
(11) (a) Pantazis, D. A.; Chen, X.-Y.; Landis, C. R.; Neese, F. All-Electron Scalar Relativistic Basis Sets for Third-Row Transition Metal Atoms. *J. Chem. Theory Comput.* **2008**, *4*, 908-919. (b) Lee, C.; Yang, W.; Parr, R. G. Development of the Colle-Salvetti correlation-energy formula into a functional of the electron density. *Phys. Rev. B* **1988**, *37*, 785-789. (c) Vosko, S. H.; Wilk, L.; Nusair, M. Accurate spin-dependent electron liquid correlation energies for local spin density calculations: a critical analysis. *Can. J. Phys.* **1980**, *58*, 1200-1211. (d) Weigend, F.; Ahlrichs, R. Balanced basis sets of split valence, triple zeta valence and quadruple zeta valence quality for H to Rn: Design and assessment of accuracy. *Phys. Chem. Chem. Phys.* **2005**, *7*, 3297-3305. (e) Grimme, S.; Antony, J.; Ehrlich, S.; Krieg, H. A consistent and accurate ab initio parametrization of density functional dispersion correction (DFT-D) for the 94 elements H-Pu. *J. Chem. Phys.* **2010**, *132*, 154104. (f) Grimme, S.; Ehrlich, S.; Goerigk, L. Effect of the damping function in dispersion corrected density functional theory. *J. Comp. Chem.* **2011**, *32*, 1456-1465. (g) Pantazis, D. A.; Neese, F. All-electron scalar relativistic basis sets for the 6p elements. *Theor Chem Acc* **2012**, *131*, 1292.
(12) Bill, E. JulX20 version 1.4, Max-Planck-Institut für Chemische Energiekonversion.
